# Missing genes in the annotation of prokaryotic genomes

**DOI:** 10.1186/1471-2105-11-131

**Published:** 2010-03-15

**Authors:** Andrew S Warren, Jeremy Archuleta, Wu-chun Feng, João Carlos Setubal

**Affiliations:** 1 Virginia Bioinformatics Institute, Virginia Tech, Blacksburg, VA, USA; 2 Department of Computer Science, Virginia Tech, Blacksburg, VA, USA

## Abstract

**Background:**

Protein-coding gene detection in prokaryotic genomes is considered a much simpler problem than in intron-containing eukaryotic genomes. However there have been reports that prokaryotic gene finder programs have problems with small genes (either over-predicting or under-predicting). Therefore the question arises as to whether current genome annotations have systematically missing, small genes.

**Results:**

We have developed a high-performance computing methodology to investigate this problem. In this methodology we compare all ORFs larger than or equal to 33 aa from all fully-sequenced prokaryotic replicons. Based on that comparison, and using conservative criteria requiring a minimum taxonomic diversity between conserved ORFs in different genomes, we have discovered 1,153 candidate genes that are missing from current genome annotations. These missing genes are similar only to each other and do not have any strong similarity to gene sequences in public databases, with the implication that these ORFs belong to missing gene families. We also uncovered 38,895 intergenic ORFs, readily identified as putative genes by similarity to currently annotated genes (we call these absent annotations). The vast majority of the missing genes found are small (less than 100 aa). A comparison of select examples with GeneMark, EasyGene and Glimmer predictions yields evidence that some of these genes are escaping detection by these programs.

**Conclusions:**

Prokaryotic gene finders and prokaryotic genome annotations require improvement for accurate prediction of small genes. The number of missing gene families found is likely a lower bound on the actual number, due to the conservative criteria used to determine whether an ORF corresponds to a real gene.

## Background

Genome annotation is a crucial step for the extraction of useful information from genomes. Yet, despite more than a decade of intensive efforts directed at improving annotation tools and at obtaining new experimental results, available annotations still suffer from a number of serious problems. The main problems regarding protein-coding genes, found in every single genome, include [1-3]: the presence of numerous bona-fide genes without any functional assignment (the so-called "hypothetical genes"); the presence of genes that are mis-annotated or with annotations that are too general to be of any use; and the possible existence of real genes that have gone undetected. In this work we address this last problem for the case of prokaryotes.

Gene prediction in prokaryotes typically involves evaluating the coding potential of genomic segments which are delimited by conserved nucleotide motifs. The most widely used gene finding programs build a gene model based on the characteristics of sequences which are likely to be real genes [4-6]. This model is then used to evaluate the likelihood that an individual segment codes for a gene. In using this method it is possible to miss genes with anomalous sequence composition. Another popular method for locating genes is to compare genomic segments with a database of gene sequences found in other organisms [7]. If the sequence is conserved (the similarity is statistically significant) across multiple genomes, the segment being evaluated is likely to be a coding gene [8] (this is the "similarity method"). Genes that do not fit a genomic pattern and do not have similar sequences in current annotation databases may be missed. If this problem occurs frequently in genome annotation projects, then many such genes may be missing from current prokaryotic annotation databases.

Finding missing or "hidden" genes in an organism will give us a more complete picture of the functional capabilities of that organism. If the organism is a pathogen, then we may be better able to control the disease it causes; if the organism is beneficial, we may be able to understand better its metabolism and hence improve its "efficiency". In any case finding all genes in a given organism improves our knowledge of the repertoire of protein-coding genes found in nature, which can lead to other discoveries.

One way to detect these missing genes is to use the similarity method to compare genomes against each other. If gene *a *in genome *A *and gene *b *in genome *B *have been missed and *a *is similar to *b*, then both may be found by comparing *A *to *B*. However, to find genes that have been systemically missed in a large annotation database such as GenBank requires a comparison of the entire prokaryotic database of genomes against itself. The computational cost of this task can be prohibitive. We have developed a methodology that has allowed us to tackle this problem. To our knowledge ours is the first large-scale attempt at identifying these missing genes in the whole of prokaryotic GenBank. We have obtained results that strongly indicate that indeed there are non-negligible numbers of genes that are being systemically missed.

## Methods

Our basic idea is to compare all Open Reading Frames (ORFs) greater than or equal to a minimum length from all fully-sequenced genomes using BLAST [9] based on a novel high-performance approach [10].

The basic genomic unit is the replicon (which can be a chromosome or a plasmid in prokaryotes). We obtained 1,297 prokaryotic replicon sequences belonging to 780 different genomes from the National Center for Biotechnology Information's RefSeq repository [11]. For each replicon we generated all maximal Open Reading Frames (ORFs) with size ≥ 99 bp by a linear scan of each replicon using start codons ATG, GTG, and TTG. These ORFs are said to be maximal because we choose the start codon that is furthest from the stop codon, without including another in-frame stop.

**Figure 1:**
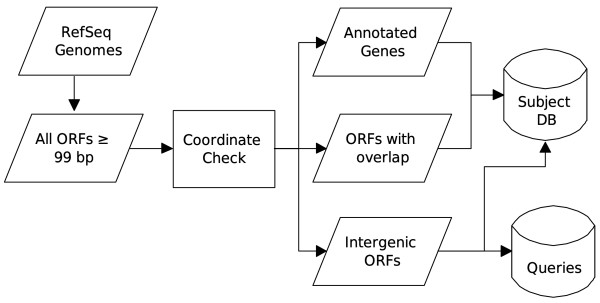
**Sequence search setup**. Process of creating subject DB and query sequences.

By comparing the coordinates of each ORF in the genomic sequence with its current annotation, we separate ORFs into three groups: (1) those that coincide with currently annotated genes; (2) those that overlap with an annotated gene or other annotated entity, e.g. RNA genes, pseudogenes, etc. (*entity-overlapping ORFs*); and (3) those that do not share genomic space with any annotated entity (*intergenic ORFs*). All ORFs were translated into amino acid sequences and used to construct a BLASTP sequence similarity search. ORFs from group 3 were used as queries and all three groups were used to create the subject database. The process for creating the queries and the subject database is shown in Figure 1.

When an ORF was found to overlap a segment of the genome occupied by another annotation, its sequence was scanned for another start codon closer to the 3' end that would remove the overlap but maintain an ORF length above the minimum 99 bp. This prevents ORFs from being classified as entity-overlapping because a false overlap was created as a result of using the most upstream start codon by default.

The BLASTP search was performed using mpiBLAST on Virginia Tech's System X supercomputer [12]. mpiBLAST parallelizes BLAST using database fragmentation, query segmentation [13], parallel input-output [14], and advanced scheduling [15]; further details are given in [10]. The BLAST search used approximately 10,000 CPU hours on System X with 15 groups of 9 CPUs running continuously between 48 and 96 hours.

The resulting alignments were then scanned for evidence of potential genes that are not represented in any of the current genome annotations (missing genes) and for gene calls that were absent in the original annotation. Alignments were screened using an e-value cutoff of 10^-5^, requiring alignment coverage for both the query and subject sequences of at least 80%, and setting the maximum number of alignments per query to 1000. The number of alignments was used to limit output file size; additional checks (described below) were carried out to ensure that this limit did not compromise results.

Using the alignment data from the BLASTP search our method labels each query as a *missing gene*, an *absent annotation*, *genomic artifact*, or as an *unclassified ORF*. Assignment of the *missing gene *label is a two-stage screening process. The first stage is an evaluation of the alignment scores and the taxonomy of subjects to which the query ORF aligns. The second stage clusters ORFs together based on a subset of the BLASTP alignments as detailed below.

### Stage 1

During *Stage 1 *a query ORF is labeled as an *absent annotation*, *genomic artifact*, or classified as a "potentially missing gene." If a query ORF (by definition an intergenic sequence) aligns significantly to another intergenic sequence, then it is considered "potentially missing". If a query ORF aligns to an annotated gene then it is classified as an absent annotation in the genome to which it belongs. If the query ORF aligns to an entity-overlapping ORF then it is classified as a genomic artifact. ORFs labeled as genomic artifact represent two possibilities. Their presence could indicate an annotation error in the replicon they align to (the ORF it aligns to is a real gene and the overlapping gene is a false prediction); or it is an alignment to a "shadow ORF" [16]. Either way, such cases were not the target of this study. Query ORFs that are not assigned to any of the above groups are labeled "unclassified." Additional file 1, Table S1 summarizes the criteria for classifying ORFs.

Because organisms that are close phylogenetically will have similar intergenic sequences due to lack of divergence, we require alignments that support a "potentially missing" classification to have subject and query sequences from two different taxonomic families, as given by organismal taxonomy in RefSeq records. In this case taxonomy is being used as a proxy for phylogenetic distance and the satisfaction of this requirement is the main evidence we use for distinguishing sequences that are likely to be real genes from sequences that represent some other conserved element. Our experience suggests that a requirement based on differing species would not be satisfactory, because there are prokaryotes classified as different species with very similar genomic sequences (for example, *Brucella *species [17]). Moreover the species and genera levels of classification have been shown to be highly variable in prokaryotes [18-21]. As such, we use the next highest taxonomic level, the family. As an additional check on sufficient phylogenetic separation, we use the MUM index [22] to score the results as described below.

The phylogenetic requirement is not used for labeling ORFs as "absent annotations" or "genomic artifact." This means that many more alignments are considered in classifying an ORF as "not missing" compared to "missing", which makes the final "missing gene" classification a much more conservative one. Any alignments to an annotated gene or to an entity-overlapping ORF by an ORF classified as potentially missing must have an average percent coverage value at least 20% less than the value for the top-scoring intergenic alignment, for that ORF be classified as a "missing gene."

### Stage 2

With absent annotations and genomic artifacts labeled in Stage 1, we proceed to Stage 2 with additional checks to confirm that the "potentially missing genes" are indeed missing. In Stage 2 the sequences classified as *potentially missing *are clustered into groups by using their BLASTP alignments as input. Only the alignments that meet the criteria in *Stage 1 *to qualify the ORF as *potentially missing *are used. We use a single-linkage approach to clustering. We say a pair of *potentially missing *genes are *connected *if there exists an alignment between them. We consider this relationship transitive so that if *x *is connected to *y *and *y *is connected to *z *then *x *is connected to *z*. Clusters are then formed by grouping connected *potentially missing *genes together. The minimum of 80% coverage rule and the kinds of alignments used ensure that few unrelated genes will be clustered together by this method. Finally, the members of those clusters which have at least two ORFs from two different taxonomic families are labeled *missing genes*.

In order to provide additional information on the correctness of the missing gene classification a series of sequence analyses were performed. After clustering, a random member was selected from each group as a representative, and its amino acid sequence was searched against the NCBI nr-aa database using a local BLASTP with an e-value cutoff of 10^-5 ^and using InterProScan [23]. For each missing gene we also used the Prodigal program [24], provided by Oak Ridge National Laboratory, to check for upstream ribosomal binding sites. In addition, for each group a replicon was selected from each taxonomic family and a genome distance metric was calculated between all possible pairings of representatives within that group. The average distance for each group was then computed. This genomic distance metric is the MUM index [22] and is based on the number of maximal unique and exact matches (MUMs) shared by two genomes. This MUM index, or MUMi, was shown to be highly correlated with average nucleotide identity [25] and generate phylogenetic trees compatible with those generated using multiple-locus sequence typing [22].

For select examples we also ran Glimmer v3.02 [5], the EasyGene prediction service [26], and GeneMarkS [27] on the replicon of origin, to determine whether the respective missing gene is detected by these gene prediction programs. While there are many gene prediction programs to choose from, we have selected Glimmer and GenMark as they are among the most widely used and EasyGene because of its ability to detect small genes. Within the GeneMark family of programs we have chosen GeneMarkS because of its provision for detecting anomalous genes. In addition, for these same examples we ran a megablastn against NCBI's nr-nt to ensure they align to intergenic regions of their respective genomes.

The RefSeq files for generating the initial three classes of ORFs and comparing them to annotations were obtained from NCBI on 07/01/2008. The missing genes were checked against a version of the nr-aa database obtained on 06/09/2009 and searched using InterproScan webservice the same date. Glimmer was run using its iterated procedure with a minimum length of 99 bp, and the default parameters: Maximum overlap = 50 and Score threshold ≥ 30. The EasyGene server was run with a default parameter of R-cutoff = 2. The GeneMarkS server was also run using default settings.

Note that the classification criteria above considers multiple pairwise alignments for each ORF. As such, some alignments might be indicative of a different classification than the one assigned (still conforming to the maximum e-value and minimum coverage rules). In order to gain insight into the variety of subject alignments for a given ORF we define a *uniqueness score α *for each missing gene and absent annotation classifications. The *α *score is a measure of the robustness of each classification, which uses information from alignments that indicate each result class. It is calculated based on the average percent identity *I *which is determined by averaging the percent identity values calculated with respect to the query and subject length. For example the percent identity with respect to the query would be the number of identities in the alignment divided by the length of the query multiplied by one hundred. The uniqueness score is calculated as *α *= *I*_1 _- *I*_2_, where *I*_1 _is always the highest *I *value from all alignments that support the classification (missing or absent) and *I*_2 _is the highest *I *value from all alignments that indicate a different classification (absent or artifact). If a query is classified as a "missing gene" and all of its alignments are to intergenic sequences then there is no *I*_2 _component and its *α *score will be equal to the highest *I*_1 _value. However, if there is an additional alignment to an entity-overlapping ORF or an annotated gene whose *I *= 60 then *α *= *I*_1 _- *I*_2 _= 80 - 60 = 20. Thus the *α *score is higher for classifications supported by alignments with high average percent identity and consistent evidence for its classification. For the missing gene classification the *α *score decreases if the query sequence aligns to other annotations (indicating an absent annotation) or to ORFs that overlap with other annotations (indicating a genomic artifact). When calculating the *α *score for absent annotations, only *I *values from alignments to entity-overlapping ORFs are used for the *I*_2 _component and so the *α *score decreases only if there are alignments indicative of the genomic artifact classification.

## Results and Discussion

**Figure 2:**
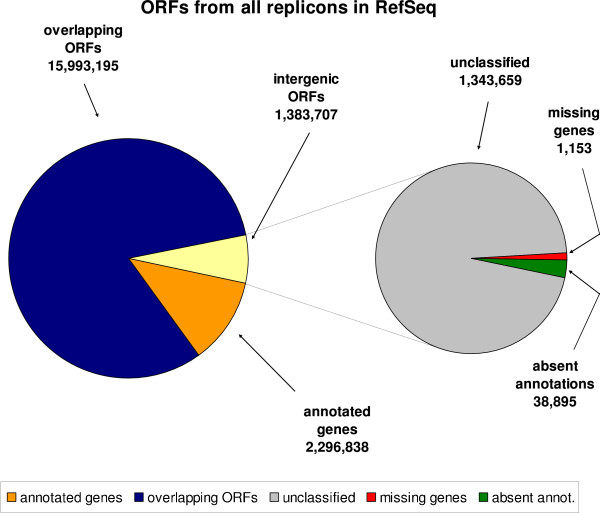
**ORF category breakdown**. All ORFs generated for prokaryotic replicons from RefSeq.

In the linear scan of 1,297 prokaryotic replicons from NCBI, 19,673,740 ORFs were generated (Figure 2). Of these 2,296,838 were found to be previously annotated as genes. The vast majority of the remaining ORFs (15,993,195) were entity-overlapping ORFs. In the generated ORF set 1,383,707 were found to occur in intergenic regions and were used as queries for BLAST. From these, our analysis has classified 1,153 ORFs as missing genes, 38,895 as absent annotations, 121,654 as genomic artifacts, and 1,222,005 remain as unclassified sequences.

**Figure 3:**
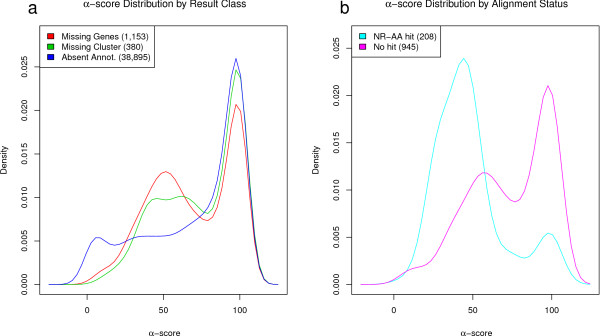
***α *score distribution**. **Panel a**: Distribution of *α *scores for missing genes, missing gene groups, and absent annotations. **Panel b**: Distribution of alpha scores for missing genes from groups that do and do not have a representative alignment to nr-aa. Density refers to kernel density [41,42]. Kernel density graphs were generated using the R sm package [42,43], where the bandwidth (smoothing parameter) is calculated as the mean of the normal optimal values for the different groups. Kernel density plots can be thought of as smooth histograms using a Gaussian function centered at each observation, instead of a box. This explains why the left and right tails extend beyond the defined bounds of the *α *function (0 and 100).

Figure 3, panel A, shows the distribution of *α *scores for absent annotations, missing genes, and the clusters of missing genes. The figure shows that many of the classifications are robust with respect to *α*. In particular, from the candidate group of missing genes, 477 have an *α *of ≥ 80. This means that each of the sequences in this missing genes subset is classified based on alignments that have an average percent identity of at least 80%. The 80% identity threshold was determined by Tian et al. [28] to be a reasonable value above which enzymatic function can be transferred with high fidelity. These sequences are highly conserved across a reasonable phylogenetic distance, and are therefore our highest-confidence gene predictions for missing genes. Additional file 2, Table S2 contains details for all missing gene predictions, sorted by decreasing *α *score.

The absent annotations with high *α*-scores may represent potential improvements to their respective annotations. Of the 38,895 absent annotations 20,413 have an *α *of ≥ 80 and 8,910 have *α*-scores of 100. The latter are intergenic in their replicon of origin, align only with annotated genes despite being searched against all possible ORFs from all RefSeq prokaryotic replicons, and have 100% identity with respect to the query and subject. One example of a currently absent annotation is an apparent catalase-peroxidase gene from the p0157 plasmid of *Escherichia coli *Sakai (a major food-borne infectious pathogen [29]), located in an intergenic region between bases 76845 and 76961 in the + strand. The protein sequence aligns with 100% identity to peroxidase sequences in UniProt [30] and has a strong scoring hit for a haem catalase-peroxidase signature in PFam [31]. Catalase-peroxidase is thought to provide protection to cells under oxidative stress [32].

**Figure 4:**
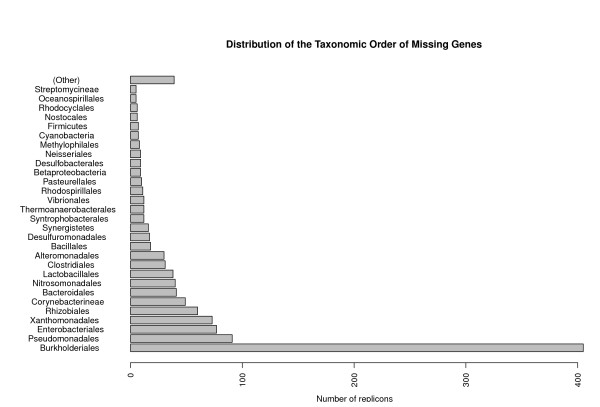
**Distribution of taxonomic orders**. The distribution of taxonomic orders among missing genes. This histogram contains more orders than Table 1, hence the category 'other' is not directly comparable.

The taxonomic breakdown of the missing genes (Table 1 and Figure 4) indicates that there are many more from the *Burkholderiales *order (35% of the total) than others. As seen in Table 2 the order *Burkholderiales *also has the highest number of intergenic ORFs, but the fraction of the total is much smaller (14%). The *Burkholderiales *also have the second highest number of replicons and the highest number of total nucleotide sequences compared to the other taxonomic orders (Table 2). This particular order also has a high average GC content (64%), which, as Skovgaard et al. [8] notes, increases the likelihood of long ORFs, in this case ≥ 99 bp, to occur by chance. Thus the problem of discriminating between short bona-fide genes and spurious ORFs is more severe in GC-rich genomes. Of the 405 missing gene predictions from *Burkholderiales*, 177 (44%) have *α*-scores < 80. It is likely that in our results the *Burkholderiales *is the order with most missing genes both because it is more represented in terms of total genome sequence and because it is an order (as a whole) for which the task of gene finding is more challenging.

**Table 1:** Taxonomic breakdown for Missing Genes.

Order		Family		Genus	
*Burkholderiales*	405	*Burkholderiaceae*	160	*Acidovorax*	83
*Pseudomonadales*	91	*Comamonadaceae*	149	*Cupriavidus*	82
*Enterobacteriales*	77	*Alcaligenaceae*	80	*Bordetella*	80
*Xanthomonadales*	73	*Enterobacteriaceae*	77	*Burkholderia*	71
*Rhizobiales*	60	*Xanthomonadaceae*	73	*Pseudomonas*	70
*Corynebacterineae*	49	*Pseudomonadaceae*	70	*Delftia*	54
*Bacteroidales*	41	*Corynebacteriaceae*	44	*Xanthomonas*	52
*Nitrosomonadales*	40	*Nitrosomonadaceae*	40	*Corynebacterium*	44
*Lactobacillales*	38	*Phyllobacteriaceae*	25	*Nitrosomonas*	40
(Other)	279	(Other)	435	(Other)	577

Most frequent Order, Class, and Genera for Missing Genes.

**Table 2:** Taxonomic breakdown for Intergenic ORFs. The top ten taxonomic Orders with respect to intergenic ORFs, number of replicons, number of bases, and avg. gc content.

Tax. Order	Num. ORFs	Frac. ORFs	Num. Replicons	Num. Mbp	Frac. Mbp	Avg. GC
*Burkholderiales*	198,068	0.14	105	246.44	0.10	0.64
*Rhizobiales*	148,195	0.11	99	196.98	0.08	0.60
*Enterobacteriales*	137,185	0.10	133	234.1	0.09	0.47
*Pseudomonadales*	63,995	0.05	42	123.62	0.05	0.49
*Corynebacterineae*	60,848	0.04	42	131.46	0.05	0.64
*Alteromonadales*	53,527	0.04	38	117.26	0.05	0.46
*Xanthomonadales*	52,402	0.04	22	51.03	0.02	0.59
*Bacillales*	51,263	0.04	48	112.49	0.04	0.38
*Clostridiales*	36,679	0.03	40	100.98	0.04	0.33
*Lactobacillales*	33,633	0.02	66	100.23	0.04	0.39
(Other)	547,912	0.40	662	1124.01	0.44	NA

Zhao et al. [33,34] have shown that the average GC frequency of the third nucleotide position of codons (GC3) in highly expressed genes, is known to average in the .80-.90 range for several species of Burkholderiales. However a correlation has also been shown [33] between gene length and codon usage bias in these same species where longer genes have higher GC3 values. As Zhao et al. suggest it is possible that selective pressure favors codons which promote greater translational accuracy for longer genes since the cost of producing the protein is proportional to its length. Under this assumption, shorter genes would have reduced selective pressure on codon bias. Indeed many of the missing genes found in *Burkholderiales *have a lower GC3 value than is found in the longer, currently annotated genes. A wide variation in codon usage bias was also found to occur among genes of *B. mallei *[33], *B. pseudomallei *[34], and several convincing examples of missing genes from this order, suggesting there is no universal rule for establishing gene identity based on this criteria in the *Burkholderiales*. The distribution of GC3 values for missing genes of the *Burkholderiales *is shown in Table 3.

**Table 3:** GC3 distribution for Burkholderiales missing genes.

0-0.1	0.1-0.2	0.2-0.3	0.3-0.4	0.4-0.5	0.5-0.6	0.6-0.7	0.7-0.8	0.8-0.9	0.9-1.0
0	0	0	2	23	129	195	73	7	0

The distribution of GC content for the third nucleotide position of codons for missing genes of the *Burkholderiales*.

The alignment-based clustering resulted in 380 groups for the 1,153 missing genes. Using a randomly selected member from each cluster a search using NCBI BLASTP against nr-aa and a search using InterProScan was performed. Of the 380 groups, 318 groups had representative sequences which do not align to anything known, 53 sequences aligned to sequences annotated with the term "hypothetical", 2 with "unknown", and 7 with some other annotation. The groups whose representative sequences have alignments against nr-aa represent two possibilities: (1) these sequences still qualified as missing genes despite their partial similarity to annotated sequences; and (2) the sequences were added to the nr-aa database (were "found") in the time between the initial data was obtained and the missing gene was searched against the nr-aa database. As seen in Figure 3 (panel B), the majority of these sequences have lower *α*-scores indicating that the partial similarity to annotated sequences was accounted for but the alignments to other intergenic ORFs was sufficient to maintain the missing gene classification. From the 380 representatives, 62 had some form of domain result when searched with InterProScan. Of those 51 had predicted signal-peptide domains and 21 had transmembrane domains. In a study of small protein coding genes in *E. coli *by Hemm et al. [35,36] many of the newly discovered, experimentally confirmed genes were found to have transmembrane domains, suggesting a potential lack of sensitivity in gene finding with respect to these types of proteins. Interestingly, 333 groups (87.6%) did not involve sequences from plasmids. Additional file 3, Table S3 has details for each missing gene group. The amino acid sequences for the missing genes are available in additional file 4, the representative nucleotide sequences are available in additional file 5, and a further breakdown of the predicted domains for each group is provided in additional file 6.

An average MUMi metric was computed for each missing gene group using representatives from each family in that group. The vast majority of missing gene groups had MUMi averages at the higher end of the MUMi scale indicating that a reasonable phylogenetic distance is represented within the majority of groups (Table 4). The average MUMi distance for each group is provided in additional file 3, Table S3. Of the 12 groups with an average MUMi distance below 0.60, 8 are created from replicons of the families *Bacteroidaceae *and *Porphyromonadaceae *of the order *Bacteroidales*, 3 from *Burkholderiaceae *and *Comamonadaceae *of the order *Burkholderiales*, and 1 from the families *Streptococcaceae *and *Leuconostoc *of the order *Lactobacillales*. All of these groups involve ORFs from plasmid sequences as a primary component in its missing gene classification which is the likely cause for low MUMi distances across family divisions. Though not all groups represent the exact same level of internal diversity, the idea here is to provide some assurance that the genomes involved are less likely to suffer from identical sequences due to recent common ancestry. No matter the qualifications used there is no exact threshold at which sequences conserved across genomes of a certain phylogenetic distance can be guaranteed to be protein coding genes. For any *in silico *method there are likely missing genes that will defy the criteria used. It is because of their anomalous nature that these genomic elements have gone undetected by the traditional annotation process.

**Table 4:** MUMi distribution for missing groups.

MUMi bins of 0.10									
0-0.1	0.1-0.2	0.2-0.3	0.3-0.4	0.4-0.5	0.5-0.6	0.6-0.7	0.7-0.8	0.8-0.9	0.9-1.0
1	8	1	0	2	0	3	4	78	283

The distribution of average MUMi values based on family level representatives for all missing gene groups. Most groups are found in the 0.9-1.0 range indicating a reasonable phylogenetic distance is represented within most groups.

For every missing gene, we used the Prodigal program [24] to predict ribosomal binding sites. We considered an RBS prediction to be useful if the upstream distance score and RBS motif score sum to a positive number and the RBS motif length was greater than or equal to 4 nucleotides. We found that 127 missing genes had an associated RBS, 70 groups had at least one sequence with an RBS, and 31 groups had an RBS associated with every gene sequence. Not all small protein coding genes will have a traditional upstream RBS motif. When studying small protein coding genes in *E. coli *Hemm et al. [35] experimentally confirmed the existence of small protein coding genes that have convincing homologues but no discernible RBS. The status of whether a missing gene has an associated RBS sequence is documented in additional file 2, Table S2.

There are 103 missing gene groups whose average *α *= 100. For these groups it is possible that ORFs occur inside non-coding elements that create highly conserved regions [37]. To test this we used MUSCLE [22] to construct multiple alignments for each group with additional 30 bp flanking regions upstream and downstream of each ORF. Of the 103 groups, 94 had perfect conservation in both the upstream and dowstream regions. Although these ORFs come from regions of ultra conservation many also contain predicted protein domains and represent an interesting phenomena among distantly related organisms. The status of ultra conservation for these groups is documented in additional file 3, Table S3 and in some of the examples below.

One example of this ultra conservation is Group 7 which is composed of 6 genes in 6 replicons and were conserved in 3 families of the order *Enterobacteriales *and 1 family of the order *Alteromonadales*. The representative amino acid sequence had no hits against the nr-aa database and the representative nucleotide sequence of 117 bp aligned to the intergenic regions of several *Escherichia coli *genome sequences, the genome sequence of *Shewanella putrefaciens *CN-32, and plasmid sequences from *Klebsiella pneumoniae*, *Serratia marcescens*, and *Cronobacter turicensis*. The InterProScan of the representative amino acid sequence indicated a signal-peptide domain from the SignalPHMM component. The sequence group has an average percent identity and an average *α*-score of 100. The coordinates for the representative sequence of this group, from *Shewanella putrefaciens *CN-32, were found to fall between gene calls made by EasyGene, Glimmer, and GeneMarkS.

Another example of this ultra-conservation comes from Group 306 which is composed of 2 genes from the families *Comamonadaceae *and *Burkholderiaceae *of the order Burkholderiales. The amino acid sequence from *Delftia acidovorans *SPH-1 has no hits when searched against the nr-aa database and the corresponding nucleotide sequence (123 bp) only aligned to intergenic regions of the *Ralstonia pickettii*, *Delftia acidovorans*, and *Ralstonia metallidurans *genomes. The amino acid sequence was found to have a nitrite reductase (large subunit) protein domain via InterProScan. The sequence group has an average *α*-score of 100. The coordinates for the representative sequence were found to fall between two gene calls made by EasyGene and GeneMark and partially overlap a call made by Glimmer.

One possible reason that short genes are missed in their respective genomes, aside from their length, is because they are foreign in origin and do not have the typical sequence characteristics of other protein coding genes within the organism. This appears to be the case for Group 20 which is composed of 4 genes from the 4 different orders *Pseudomonadales*, *Burkholderiales*, *Sphingomonadales*, and *Xanthomonadales*. The amino acid sequence from *Pseudomonas aeruginosa *PA7 has no hits against the nr-aa database. Though this group does not have a traditional upstream RBS motif or known protein domains, it is conserved across four different orders of bacteria and its representative nucleotide sequence (102 nt) aligns only to intergenic regions in the respective genomes of origin. This gene was found to be consistently flanked by a phage related integrase protein in each genome and for the copy in the *Burkholderiales *genome it has a GC content of 0.38 which is uncharacteristically low. All this suggests that it may be foreign in origin. The group has an average *alpha*-score of 90. The coordinates for the representative sequence of this group were found to fall between gene calls made by Glimmer, GeneMarkS, and EasyGene.

Another group of interest is Group 338 which has 2 genes from the families *Shewanellaceae *and *Pseudoalteromonadaceae *of the order *Alteromonadales*. The representative amino acid sequence from *Pseudoalteromonas atlantica *T6c did not have any hits when searched against the nr-aa database and the nucleotide sequence (150 nt) only aligned to intergenic regions of the two genomes. In addition an RBS motif was found upstream of both sequences using the Prodigal program [24]. A transmembrane region and signal-peptide domain were detected by InterProScan. The group has an average *α*-score of 59. The coordinates for the representative sequence of this group were predicted by Glimmer and GeneMarkS and fell between the predictions made by EasyGene.

**Figure 5:**
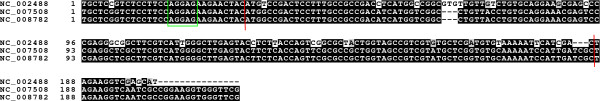
**Missing gene group multiple alignment**. A multiple alignment of missing gene Group 32. The green box shows the upstream RBS site "AGGAG". And the red lines mark the boundaries of the conserved ORF. The multiple alignment includes an additional 30 bp upstream and downstream of genomic DNA.

Group 32 is composed of 3 genes, 2 from the order *Xanthomonadales *and 1 from *Burkholderiales*. The representative amino acid sequence from *Xylella fastidiosa *9a5c did not have hits when searched against nr-aa and the nucleotide sequence (141 nt) only aligned to an intergenic region of the *Xylella *genome. As seen in the multiple alignment in Figure 5 the sequences from *Xanthomonas campestris *and *Acidovorax *sp. JS42 suggest a region of high conservation with an embedded ORF that occurs by chance. However, the sequence from *Xylella *does not maintain this high level of conservation in the downstream region or in the ORF sequence. In addition, an RBS motif was found upstream of all three sequences suggesting that this may be a real gene that happens to fall in a region of high conservation in two of the three organisms. The group has an average *α*-score of 88. The coordinates for the representative sequence of this group were predicted by Glimmer and GeneMarkS and fell between the predictions made by EasyGene.

Our final example (Group 11) is composed of 5 genes in 5 replicons and has no hits to the nr-aa database or InterProScan. Of these 3 replicons come from *Leucnostoc *(*Firmicutes*, *Lactobacillales*) and 2 *Streptococcus *(*Lactobacillales*, *Streptococcaceae*). The megablastn of the 140 nt sequence aligned only to the intergenic regions of their genomic sequences. The group has an average percent identity and an average *α*-score of 91. The coordinates for the representative sequence of this group, from *Leuconostoc citreum *KM20, were found to fall between two gene calls made by EasyGene and Glimmer and partially overlap a call made by GeneMarkS.

**Figure 6:**
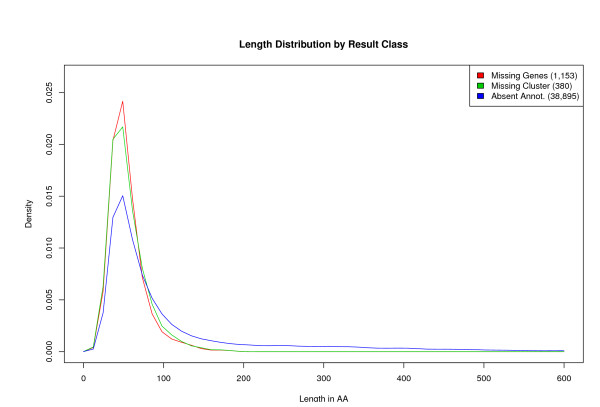
**Length distribution**. Distribution of sequence length for missing genes, missing gene groups, and absent annotations.

The vast majority of missing genes and absent annotations had sizes smaller than 100 amino acids (Figure 6). With respect to missing genes, it is likely that their shorter length played a role in why these sequences were not found or ignored in their respective genome annotations. While their shorter length and lack of similarity to experimentally verified genes raises the question whether these short ORFs are real protein-coding genes, the strong similarity, and in some cases complete identity, across different prokaryotic families should not be ignored.

## Conclusions

Our results clearly show that there are indeed likely protein-coding genes in prokaryotic genomes that have been systemically missed. Our results of about 380 such families in 780 genomes should be considered a lower bound on the actual number of missing or hidden gene families, because the criteria we have used to find them are rather conservative. There were 1,121,362 intergenic sequences that went "unclassified," and such ORFs are a clear target for finding additional genes.

The vast majority of the genes we found are small, under 100 aa (Figure 5). In addition, in the small sample we analyzed with Glimmer, EasyGene and GeneMark, we found that these gene finders did not detect some of these potentially missing genes. If traditional indicators of protein coding genes were present for these small proteins then many of them would already be present in the protein sequence database. As in previous studies [35], these results suggest that some small protein-coding genes remain a problem for prokaryotic gene finders, in spite of the progress made by EasyGene [4], Glimmer [5], GeneMarkS [27], and others. And when small genes are predicted by these programs, they run the risk of being ignored by annotation projects in order to avoid over-prediction.

In order to find the missing genes using all fully-sequenced prokaryotic genomes we have used an innovative high-performance methodology [10]. That methodology can be potentially used for other genome database-wide surveys of sequence data. The performance of both mpiBLAST [13] and ParaMEDIC [10] theoretically scales well if the number of processors that are used scales as the square of the number of replicon sequences.

Although our focus was on missing genes, the class *absent annotations *is also a useful result, and could be used to improve the annotations of genomes where they are located.

We would like to refine our criteria for filtering candidate missing genes. In this work we have used a rather simple filter based on the taxonomic family. Such a filter is overly conservative in genera that contain species with quite distinct genomic compositions, such as the *Pseudomonadaceae *[38]. Furthermore the NCBI taxonomy used in this study is not an authoritative source but is one of the few resources where this information is readily available for such a wide range of organisms. A more sophisticated and sensitive method of screening may be used, such as classifying sequences based on whether the Ka/Ks ratio indicates a lack of selective pressure [39], but it is not clear how well this will perform with short sequences. Protein coding genes smaller than our current 99 bp threshold are known to exist [35]. It may be possible to refine our method to allow for the detection of genes under its current limit.

The proof that our predicted missing genes are real genes needs to come from experimentation. Obtaining this proof should be achievable in the near future with the use of large-scale prokaryotic transcriptomics, made practical, and financially viable, by next-generation sequencing technologies [40].

## Authors' contributions

AW contributed to the writing of this work, is the main programmer for the project, and conceived the performance measurement and missing gene determination techniques. JA planned, constructed, and performed the mpiBLAST aspects and provided valuable information and feedback with respect to the pipeline and in the writing process. WF provided funding and guidance for this work and valuable insight into the High Performance computing aspects and gave valuable insight into the writing of the manuscript. JS conceived the initial project idea, provided funding and guidance for this work, and has contributed to the interpretation of data and writing of the manuscript. All authors have read and approved the final manuscript.

## Supplementary Material

Additional file 1 **Table S1**. Criteria for classifying ORFs. An ORF must meet all the requirements for a particular category to be classified in that category. Click here for file

Additional file 2 **Table S2**. Details for all missing genes. Includes NCBI Refseq ID for the replicon of origin, unique (per replicon) gene ID, Start bp coordinate, Stop bp coordinate, Length (AA), *α *score, Cluter ID, boolean whether the rep-sequence has a hit to nr-aa, boolean whether it has a hit to InterPro, Taxonomic Order, Family Genus, and whether the sequence has a predicted upstream RBS. Click here for file

Additional file 3 **Table S3**. Details for all missing gene groups. Includes cluster id, average *α*-score, average length, average percent identity, whether the representative sequence had a hit against nr-aa, whether the sequence had a domain result from interproscan, the e-value of the hit to nr-aa, the percent identity of hit to nr-aa, the number of replicons in the group, number of chromsomes, number of plasmids, average MUMi value between families in the group, and whether the multiple alignment of the group indicated a region of ultra-conservation. Click here for file

Additional file 4 **AA sequences file**. Amino acid sequences for the missing genes. Click here for file

Additional file 5 **NT sequences file**. Representative nucleotide sequences for each missing gene group. Click here for file

Additional file 6 **InterPro domain results**. InterProScan results for the representative amino acid sequence for each group. Click here for file
