# Supplementary material for: Missing genes in the annotation of prokaryotic genomes
Source: BMC Bioinformatics. 2010 Mar 15;11:131. doi: 10.1186/1471-2105-11-131 (PMC3098052; doi:10.1186/1471-2105-11-131)
Supplement: Additional file 1 — Table S1. Criteria for classifying ORFs. An ORF must meet all the requirements for a particular category to be classified in that category. [file 1471-2105-11-131-S1.PDF]

Table S1 Classification Criteria

Absent Annotations

Best e-value and % identity from annotated gene alignment
Alignment Subject from a different replicon
Alignment >=80% coverage for query and subject

Genomic Artifacts

Best e-value and % identity from align. to ORF that overlaps a real gene
Alignment Subject from a different replicon
Alignment >=80% coverage for query and subject

Potentially Missing

Best e-value and % identity from intergenic ORF alignment
Alignment Subject from a different taxonomic family (defined by NCBI)
Alignment >=80% coverage for query and subject
20% margin for average coverage
