# Supplementary material for: Missing genes in the annotation of prokaryotic genomes
Source: BMC Bioinformatics. 2010 Mar 15;11:131. doi: 10.1186/1471-2105-11-131 (PMC3098052; doi:10.1186/1471-2105-11-131)
Supplement: Additional file 2 — Table S2. Details for all missing genes. Includes NCBI Refseq ID for the replicon of origin, unique (per replicon) gene ID, Start bp coordinate, Stop bp coordinate, Length (AA), α score, Cluter ID, boolean whether the rep-sequence has a hit to nr-aa, boolean whether it has a hit to InterPro, Taxonomic Order, Family Genus, and whether the sequence has a predicted upstream RBS. [file 1471-2105-11-131-S2.PDF]

Replicon	Start	Stop	AA Length	Alpha-Score	Cluster ID	HasNR	HasPro	Order	Family	Genus	RBS
NC_008573	190900	191019	39	100	7	FALSE	TRUE	Alteromonadales	Shewanellaceae	Shewanella	FALSE
NC_009438	285981	286100	39	100	7	FALSE	TRUE	Alteromonadales	Shewanellaceae	Shewanella	FALSE
NC_009778	4214040	4214159	39	100	7	FALSE	TRUE	Enterobacteriales	Enterobacteriaceae	Cronobacter	FALSE
NC_009649	79985	79866	39	100	7	FALSE	TRUE	Enterobacteriales	Enterobacteriaceae	Klebsiella	FALSE
NC_009838	134371	134490	39	100	7	FALSE	TRUE	Enterobacteriales	Enterobacteriaceae	Escherichia	FALSE
NC_010468	3734643	3734524	39	100	7	FALSE	TRUE	Enterobacteriales	Enterobacteriaceae	Escherichia	FALSE
NC_006856	1475	1579	34	100	67	FALSE	FALSE	Enterobacteriales	Enterobacteriaceae	Salmonella	FALSE
NC_010410	3640820	3640924	34	100	67	FALSE	FALSE	Pseudomonadales	Moraxellaceae	Acinetobacter	FALSE
NC_010488	122800	122696	34	100	67	FALSE	FALSE	Enterobacteriales	Enterobacteriaceae	Escherichia	FALSE
NC_008344	679346	679233	37	100	60	FALSE	FALSE	Nitrosomonadales	Nitrosomonadaceae	Nitrosomonas	FALSE
NC_004741	2605885	2605772	37	100	60	FALSE	FALSE	Enterobacteriales	Enterobacteriaceae	Shigella	FALSE
NC_008344	2385914	2386027	37	100	60	FALSE	FALSE	Nitrosomonadales	Nitrosomonadaceae	Nitrosomonas	FALSE
NC_009085	792681	792794	37	100	60	FALSE	FALSE	Pseudomonadales	Moraxellaceae	Acinetobacter	FALSE
NC_008344	683849	683736	37	100	57	FALSE	FALSE	Nitrosomonadales	Nitrosomonadaceae	Nitrosomonas	FALSE
NC_004741	2610384	2610271	37	100	57	FALSE	FALSE	Enterobacteriales	Enterobacteriaceae	Shigella	FALSE
NC_008344	2381411	2381524	37	100	57	FALSE	FALSE	Nitrosomonadales	Nitrosomonadaceae	Nitrosomonas	FALSE
NC_009085	788181	788294	37	100	57	FALSE	FALSE	Pseudomonadales	Moraxellaceae	Acinetobacter	FALSE
NC_008344	683180	683073	35	100	55	FALSE	TRUE	Nitrosomonadales	Nitrosomonadaceae	Nitrosomonas	FALSE
NC_009085	788850	788957	35	100	55	FALSE	TRUE	Pseudomonadales	Moraxellaceae	Acinetobacter	FALSE
NC_008344	2382080	2382187	35	100	55	FALSE	TRUE	Nitrosomonadales	Nitrosomonadaceae	Nitrosomonas	FALSE
NC_004741	2609715	2609608	35	100	55	FALSE	TRUE	Enterobacteriales	Enterobacteriaceae	Shigella	FALSE
NC_009085	788837	788968	43	100	54	TRUE	FALSE	Pseudomonadales	Moraxellaceae	Acinetobacter	FALSE
NC_008344	2382067	2382198	43	100	54	TRUE	FALSE	Nitrosomonadales	Nitrosomonadaceae	Nitrosomonas	FALSE
NC_008344	683193	683062	43	100	54	TRUE	FALSE	Nitrosomonadales	Nitrosomonadaceae	Nitrosomonas	FALSE
NC_004741	2609728	2609597	43	100	54	TRUE	FALSE	Enterobacteriales	Enterobacteriaceae	Shigella	FALSE
NC_008344	2381864	2382070	68	100	53	FALSE	FALSE	Nitrosomonadales	Nitrosomonadaceae	Nitrosomonas	FALSE
NC_004741	2609931	2609725	68	100	53	FALSE	FALSE	Enterobacteriales	Enterobacteriaceae	Shigella	FALSE
NC_009085	788634	788840	68	100	53	FALSE	FALSE	Pseudomonadales	Moraxellaceae	Acinetobacter	FALSE
NC_008344	683396	683190	68	100	53	FALSE	FALSE	Nitrosomonadales	Nitrosomonadaceae	Nitrosomonas	FALSE
NC_004741	2609831	2609712	39	100	52	FALSE	FALSE	Enterobacteriales	Enterobacteriaceae	Shigella	FALSE
NC_008344	2381964	2382083	39	100	52	FALSE	FALSE	Nitrosomonadales	Nitrosomonadaceae	Nitrosomonas	FALSE
NC_008344	683296	683177	39	100	52	FALSE	FALSE	Nitrosomonadales	Nitrosomonadaceae	Nitrosomonas	FALSE
NC_009085	788734	788853	39	100	52	FALSE	FALSE	Pseudomonadales	Moraxellaceae	Acinetobacter	FALSE
NC_009085	789162	789028	44	100	51	FALSE	FALSE	Pseudomonadales	Moraxellaceae	Acinetobacter	FALSE
NC_008344	682868	683002	44	100	51	FALSE	FALSE	Nitrosomonadales	Nitrosomonadaceae	Nitrosomonas	FALSE
NC_004741	2609403	2609537	44	100	51	FALSE	FALSE	Enterobacteriales	Enterobacteriaceae	Shigella	FALSE
NC_008344	2382392	2382258	44	100	51	FALSE	FALSE	Nitrosomonadales	Nitrosomonadaceae	Nitrosomonas	FALSE
NC_008344	2382718	2382617	33	100	50	FALSE	FALSE	Nitrosomonadales	Nitrosomonadaceae	Nitrosomonas	FALSE
NC_004741	2609077	2609178	33	100	50	FALSE	FALSE	Enterobacteriales	Enterobacteriaceae	Shigella	FALSE
NC_009085	789488	789387	33	100	50	FALSE	FALSE	Pseudomonadales	Moraxellaceae	Acinetobacter	FALSE
NC_008344	682542	682643	33	100	50	FALSE	FALSE	Nitrosomonadales	Nitrosomonadaceae	Nitrosomonas	FALSE
NC_009085	796674	796799	41	100	49	TRUE	TRUE	Pseudomonadales	Moraxellaceae	Acinetobacter	FALSE
NC_009651	21571	21446	41	100	49	TRUE	TRUE	Enterobacteriales	Enterobacteriaceae	Klebsiella	FALSE
NC_004741	2601892	2601767	41	100	49	TRUE	TRUE	Enterobacteriales	Enterobacteriaceae	Shigella	FALSE
NC_010410	3654632	3654522	36	100	363	FALSE	TRUE	Pseudomonadales	Moraxellaceae	Acinetobacter	FALSE
NC_009651	33982	33872	36	100	363	FALSE	TRUE	Enterobacteriales	Enterobacteriaceae	Klebsiella	FALSE
NC_008260	749277	749390	37	100	346	FALSE	FALSE	Oceanospirillales	Alcanivoracaceae	Alcanivorax	FALSE
NC_008740	3565394	3565281	37	100	346	FALSE	FALSE	Alteromonadales	Alteromonadaceae	Marinobacter	FALSE
NC_008740	3564501	3564394	35	100	345	FALSE	TRUE	Alteromonadales	Alteromonadaceae	Marinobacter	FALSE
NC_008260	750170	750277	35	100	345	FALSE	TRUE	Oceanospirillales	Alcanivoracaceae	Alcanivorax	FALSE
NC_008260	750328	750200	42	100	343	FALSE	FALSE	Oceanospirillales	Alcanivoracaceae	Alcanivorax	FALSE
NC_008740	3564343	3564471	42	100	343	FALSE	FALSE	Alteromonadales	Alteromonadaceae	Marinobacter	FALSE
NC_008260	735681	735505	58	100	342	FALSE	FALSE	Oceanospirillales	Alcanivoracaceae	Alcanivorax	FALSE

NC_008740	3578990	3579166	58	100	342	FALSE	FALSE	Alteromonadales	Alteromonadaceae	Marinobacter	FALSE
NC_010170	4574236	4574481	81	100	337	FALSE	FALSE	Burkholderiales	Alcaligenaceae	Bordetella	FALSE
NC_010002	4534080	4533835	81	100	337	FALSE	FALSE	Burkholderiales	Comamonadaceae	Delftia	FALSE
NC_009719	3627372	3627482	36	100	333	FALSE	FALSE	Rhizobiales	Phyllobacteriaceae	Parvibaculum	FALSE
NC_007973	2521662	2521772	36	100	333	FALSE	FALSE	Burkholderiales	Burkholderiaceae	Cupriavidus	FALSE
NC_007973	3249393	3249500	35	100	330	FALSE	FALSE	Burkholderiales	Burkholderiaceae	Cupriavidus	FALSE
NC_010002	2942535	2942642	35	100	330	FALSE	FALSE	Burkholderiales	Comamonadaceae	Delftia	FALSE
NC_010002	2942594	2942857	87	100	329	FALSE	FALSE	Burkholderiales	Comamonadaceae	Delftia	FALSE
NC_007973	3249452	3249715	87	100	329	FALSE	FALSE	Burkholderiales	Burkholderiaceae	Cupriavidus	FALSE
NC_010002	2942700	2942599	33	100	328	FALSE	FALSE	Burkholderiales	Comamonadaceae	Delftia	FALSE
NC_007973	3249558	3249457	33	100	328	FALSE	FALSE	Burkholderiales	Burkholderiaceae	Cupriavidus	FALSE
NC_010002	2942889	2942776	37	100	327	FALSE	FALSE	Burkholderiales	Comamonadaceae	Delftia	FALSE
NC_007973	3249747	3249634	37	100	327	FALSE	FALSE	Burkholderiales	Burkholderiaceae	Cupriavidus	FALSE
NC_010002	2942911	2943030	39	100	326	FALSE	FALSE	Burkholderiales	Comamonadaceae	Delftia	FALSE
NC_007973	3249769	3249888	39	100	326	FALSE	FALSE	Burkholderiales	Burkholderiaceae	Cupriavidus	FALSE
NC_010170	4568328	4568200	42	100	324	FALSE	FALSE	Burkholderiales	Alcaligenaceae	Bordetella	FALSE
NC_010002	4539988	4540116	42	100	324	FALSE	FALSE	Burkholderiales	Comamonadaceae	Delftia	FALSE
NC_010002	4545196	4545312	38	100	321	FALSE	FALSE	Burkholderiales	Comamonadaceae	Delftia	FALSE
NC_010170	4563121	4563005	38	100	321	FALSE	FALSE	Burkholderiales	Alcaligenaceae	Bordetella	TRUE
NC_010002	4531690	4531556	44	100	320	FALSE	FALSE	Burkholderiales	Comamonadaceae	Delftia	FALSE
NC_010170	4576626	4576760	44	100	320	FALSE	FALSE	Burkholderiales	Alcaligenaceae	Bordetella	FALSE
NC_010170	4482019	4482195	58	100	318	FALSE	TRUE	Burkholderiales	Alcaligenaceae	Bordetella	FALSE
NC_010002	4612777	4612601	58	100	318	FALSE	TRUE	Burkholderiales	Comamonadaceae	Delftia	FALSE
NC_010002	2943049	2942846	67	100	315	FALSE	FALSE	Burkholderiales	Comamonadaceae	Delftia	FALSE
NC_007973	3249907	3249704	67	100	315	FALSE	FALSE	Burkholderiales	Burkholderiaceae	Cupriavidus	FALSE
NC_010002	3006221	3006120	33	100	313	FALSE	FALSE	Burkholderiales	Comamonadaceae	Delftia	FALSE
NC_007973	3291198	3291097	33	100	313	FALSE	FALSE	Burkholderiales	Burkholderiaceae	Cupriavidus	FALSE
NC_009719	3627389	3627529	46	100	312	FALSE	FALSE	Rhizobiales	Phyllobacteriaceae	Parvibaculum	FALSE
NC_007973	2521679	2521819	46	100	312	FALSE	FALSE	Burkholderiales	Burkholderiaceae	Cupriavidus	FALSE
NC_007973	3249675	3249815	46	100	309	FALSE	FALSE	Burkholderiales	Burkholderiaceae	Cupriavidus	FALSE
NC_010002	2942817	2942957	46	100	309	FALSE	FALSE	Burkholderiales	Comamonadaceae	Delftia	FALSE
NC_010002	3007724	3007617	35	100	307	FALSE	FALSE	Burkholderiales	Comamonadaceae	Delftia	FALSE
NC_007973	3292701	3292594	35	100	307	FALSE	FALSE	Burkholderiales	Burkholderiaceae	Cupriavidus	FALSE
NC_010002	3007690	3007565	41	100	306	FALSE	TRUE	Burkholderiales	Comamonadaceae	Delftia	FALSE
NC_007973	3292667	3292542	41	100	306	FALSE	TRUE	Burkholderiales	Burkholderiaceae	Cupriavidus	FALSE
NC_007973	3253611	3253447	54	100	303	FALSE	TRUE	Burkholderiales	Burkholderiaceae	Cupriavidus	FALSE
NC_010002	2946753	2946589	54	100	303	FALSE	TRUE	Burkholderiales	Comamonadaceae	Delftia	FALSE
NC_010002	2999961	3000080	39	100	298	FALSE	FALSE	Burkholderiales	Comamonadaceae	Delftia	FALSE
NC_007973	3284938	3285057	39	100	298	FALSE	FALSE	Burkholderiales	Burkholderiaceae	Cupriavidus	FALSE
NC_010002	3000087	3000191	34	100	297	FALSE	FALSE	Burkholderiales	Comamonadaceae	Delftia	FALSE
NC_007973	3285064	3285168	34	100	297	FALSE	FALSE	Burkholderiales	Burkholderiaceae	Cupriavidus	FALSE
NC_007973	3284008	3283904	34	100	293	FALSE	FALSE	Burkholderiales	Burkholderiaceae	Cupriavidus	FALSE
NC_010002	2999031	2998927	34	100	293	FALSE	FALSE	Burkholderiales	Comamonadaceae	Delftia	FALSE
NC_010002	2999489	2999599	36	100	290	FALSE	FALSE	Burkholderiales	Comamonadaceae	Delftia	FALSE
NC_007973	3284466	3284576	36	100	290	FALSE	FALSE	Burkholderiales	Burkholderiaceae	Cupriavidus	FALSE
NC_007973	2518156	2518040	38	100	288	FALSE	FALSE	Burkholderiales	Burkholderiaceae	Cupriavidus	FALSE
NC_009719	3623866	3623750	38	100	288	FALSE	FALSE	Rhizobiales	Phyllobacteriaceae	Parvibaculum	FALSE
NC_008782	1388815	1388916	33	100	287	FALSE	FALSE	Burkholderiales	Comamonadaceae	Acidovorax	FALSE
NC_007973	2509851	2509750	33	100	287	FALSE	FALSE	Burkholderiales	Burkholderiaceae	Cupriavidus	FALSE
NC_008782	1388852	1388956	34	100	286	FALSE	FALSE	Burkholderiales	Comamonadaceae	Acidovorax	FALSE
NC_007973	2509814	2509710	34	100	286	FALSE	FALSE	Burkholderiales	Burkholderiaceae	Cupriavidus	FALSE
NC_010002	2998845	2999042	65	100	282	FALSE	FALSE	Burkholderiales	Comamonadaceae	Delftia	FALSE
NC_007973	3283822	3284019	65	100	282	FALSE	FALSE	Burkholderiales	Burkholderiaceae	Cupriavidus	FALSE
NC_010002	3007538	3007645	35	100	281	FALSE	FALSE	Burkholderiales	Comamonadaceae	Delftia	FALSE

NC_007973	3292515	3292622	35	100	281	FALSE	FALSE	Burkholderiales	Burkholderiaceae	Cupriavidus	FALSE
NC_007973	2526869	2526738	43	100	280	FALSE	TRUE	Burkholderiales	Burkholderiaceae	Cupriavidus	FALSE
NC_009719	3632585	3632454	43	100	280	FALSE	TRUE	Rhizobiales	Phyllobacteriaceae	Parvibaculum	FALSE
NC_009719	3632473	3632574	33	100	279	FALSE	TRUE	Rhizobiales	Phyllobacteriaceae	Parvibaculum	FALSE
NC_007973	2526757	2526858	33	100	279	FALSE	TRUE	Burkholderiales	Burkholderiaceae	Cupriavidus	FALSE
NC_007973	2520963	2520697	88	100	278	FALSE	FALSE	Burkholderiales	Burkholderiaceae	Cupriavidus	FALSE
NC_009719	3626673	3626407	88	100	278	FALSE	FALSE	Rhizobiales	Phyllobacteriaceae	Parvibaculum	FALSE
NC_009719	3626568	3626702	44	100	277	FALSE	FALSE	Rhizobiales	Phyllobacteriaceae	Parvibaculum	FALSE
NC_007973	2520858	2520992	44	100	277	FALSE	FALSE	Burkholderiales	Burkholderiaceae	Cupriavidus	FALSE
NC_008757	15125	14895	76	100	274	FALSE	FALSE	Burkholderiales	Comamonadaceae	Polaromonas	FALSE
NC_007953	1243140	1242910	76	100	274	FALSE	FALSE	Burkholderiales	Burkholderiaceae	Burkholderia	FALSE
NC_007953	1242828	1243043	71	100	273	FALSE	FALSE	Burkholderiales	Burkholderiaceae	Burkholderia	FALSE
NC_008757	14813	15028	71	100	273	FALSE	FALSE	Burkholderiales	Comamonadaceae	Polaromonas	FALSE
NC_008757	14908	14750	52	100	272	FALSE	FALSE	Burkholderiales	Comamonadaceae	Polaromonas	FALSE
NC_007953	1242923	1242765	52	100	272	FALSE	FALSE	Burkholderiales	Burkholderiaceae	Burkholderia	FALSE
NC_007953	1243190	1243068	40	100	271	FALSE	FALSE	Burkholderiales	Burkholderiaceae	Burkholderia	FALSE
NC_008757	15175	15053	40	100	271	FALSE	FALSE	Burkholderiales	Comamonadaceae	Polaromonas	FALSE
NC_008757	14736	14873	45	100	268	FALSE	FALSE	Burkholderiales	Comamonadaceae	Polaromonas	FALSE
NC_007953	1242751	1242888	45	100	268	FALSE	FALSE	Burkholderiales	Burkholderiaceae	Burkholderia	FALSE
NC_010170	1591346	1591462	38	100	267	FALSE	FALSE	Burkholderiales	Alcaligenaceae	Bordetella	FALSE
NC_007951	3677948	3678064	38	100	267	FALSE	FALSE	Burkholderiales	Burkholderiaceae	Burkholderia	FALSE
NC_010170	1559905	1559786	39	100	265	FALSE	FALSE	Burkholderiales	Alcaligenaceae	Bordetella	FALSE
NC_007951	3614149	3614030	39	100	265	FALSE	FALSE	Burkholderiales	Burkholderiaceae	Burkholderia	FALSE
NC_007951	3650219	3650112	35	100	264	FALSE	TRUE	Burkholderiales	Burkholderiaceae	Burkholderia	FALSE
NC_010170	1581824	1581717	35	100	264	FALSE	TRUE	Burkholderiales	Alcaligenaceae	Bordetella	FALSE
NC_010170	1587878	1587979	33	100	263	FALSE	TRUE	Burkholderiales	Alcaligenaceae	Bordetella	FALSE
NC_007951	3656276	3656377	33	100	263	FALSE	TRUE	Burkholderiales	Burkholderiaceae	Burkholderia	FALSE
NC_010170	1493689	1493564	41	100	262	FALSE	FALSE	Burkholderiales	Alcaligenaceae	Bordetella	FALSE
NC_007951	3559569	3559444	41	100	262	FALSE	FALSE	Burkholderiales	Burkholderiaceae	Burkholderia	FALSE
NC_007951	3559641	3559799	52	100	261	FALSE	FALSE	Burkholderiales	Burkholderiaceae	Burkholderia	FALSE
NC_010170	1493761	1493919	52	100	261	FALSE	FALSE	Burkholderiales	Alcaligenaceae	Bordetella	FALSE
NC_010170	1580831	1580727	34	100	260	FALSE	FALSE	Burkholderiales	Alcaligenaceae	Bordetella	FALSE
NC_007951	3649226	3649122	34	100	260	FALSE	FALSE	Burkholderiales	Burkholderiaceae	Burkholderia	FALSE
NC_007951	3559718	3559858	46	100	259	FALSE	FALSE	Burkholderiales	Burkholderiaceae	Burkholderia	FALSE
NC_010170	1493838	1493978	46	100	259	FALSE	FALSE	Burkholderiales	Alcaligenaceae	Bordetella	FALSE
NC_007951	3560182	3559883	99	100	257	FALSE	TRUE	Burkholderiales	Burkholderiaceae	Burkholderia	FALSE
NC_010170	1494302	1494003	99	100	257	FALSE	TRUE	Burkholderiales	Alcaligenaceae	Bordetella	FALSE
NC_010170	1494143	1494319	58	100	255	FALSE	FALSE	Burkholderiales	Alcaligenaceae	Bordetella	FALSE
NC_007951	3560023	3560199	58	100	255	FALSE	FALSE	Burkholderiales	Burkholderiaceae	Burkholderia	FALSE
NC_007951	3656095	3656286	63	100	254	TRUE	TRUE	Burkholderiales	Burkholderiaceae	Burkholderia	FALSE
NC_010170	1587697	1587888	63	100	254	TRUE	TRUE	Burkholderiales	Alcaligenaceae	Bordetella	FALSE
NC_009085	784605	784781	58	100	25	TRUE	TRUE	Pseudomonadales	Moraxellaceae	Acinetobacter	FALSE
NC_009651	18183	18007	58	100	25	TRUE	TRUE	Enterobacteriales	Enterobacteriaceae	Klebsiella	FALSE
NC_004741	2613960	2613784	58	100	25	TRUE	TRUE	Enterobacteriales	Enterobacteriaceae	Shigella	FALSE
NC_008344	2377835	2378011	58	100	25	TRUE	TRUE	Nitrosomonadales	Nitrosomonadaceae	Nitrosomonas	FALSE
NC_008344	687425	687249	58	100	25	TRUE	TRUE	Nitrosomonadales	Nitrosomonadaceae	Nitrosomonas	FALSE
NC_007951	3566015	3566179	54	100	248	FALSE	FALSE	Burkholderiales	Burkholderiaceae	Burkholderia	FALSE
NC_010170	1500135	1500299	54	100	248	FALSE	FALSE	Burkholderiales	Alcaligenaceae	Bordetella	FALSE
NC_007951	3566100	3565993	35	100	246	FALSE	FALSE	Burkholderiales	Burkholderiaceae	Burkholderia	FALSE
NC_010170	1500220	1500113	35	100	246	FALSE	FALSE	Burkholderiales	Alcaligenaceae	Bordetella	FALSE
NC_010170	1504549	1504680	43	100	243	FALSE	FALSE	Burkholderiales	Alcaligenaceae	Bordetella	FALSE
NC_007951	3570429	3570560	43	100	243	FALSE	FALSE	Burkholderiales	Burkholderiaceae	Burkholderia	FALSE
NC_010170	1588227	1588105	40	100	242	FALSE	FALSE	Burkholderiales	Alcaligenaceae	Bordetella	FALSE
NC_007951	3656625	3656503	40	100	242	FALSE	FALSE	Burkholderiales	Burkholderiaceae	Burkholderia	FALSE

NC_007951	3656540	3656662	40	100	241	FALSE	FALSE	Burkholderiales	Burkholderiaceae	Burkholderia	TRUE
NC_010170	1588142	1588264	40	100	241	FALSE	FALSE	Burkholderiales	Alcaligenaceae	Bordetella	TRUE
NC_007951	3559973	3559836	45	100	240	FALSE	TRUE	Burkholderiales	Burkholderiaceae	Burkholderia	FALSE
NC_010170	1494093	1493956	45	100	240	FALSE	TRUE	Burkholderiales	Alcaligenaceae	Bordetella	FALSE
NC_008344	2377855	2377968	37	100	24	FALSE	TRUE	Nitrosomonadales	Nitrosomonadaceae	Nitrosomonas	FALSE
NC_009085	784625	784738	37	100	24	FALSE	TRUE	Pseudomonadales	Moraxellaceae	Acinetobacter	FALSE
NC_008344	687405	687292	37	100	24	FALSE	TRUE	Nitrosomonadales	Nitrosomonadaceae	Nitrosomonas	FALSE
NC_009651	18163	18050	37	100	24	FALSE	TRUE	Enterobacteriales	Enterobacteriaceae	Klebsiella	FALSE
NC_004741	2613940	2613827	37	100	24	FALSE	TRUE	Enterobacteriales	Enterobacteriaceae	Shigella	FALSE
NC_010473	1370285	1370184	33	100	225	FALSE	FALSE	Enterobacteriales	Enterobacteriaceae	Escherichia	FALSE
NC_007626	2159285	2159184	33	100	225	FALSE	FALSE	Rhodospirillales	Rhodospirillaceae	Magnetospirillum	FALSE
NC_007508	2670244	2670363	39	100	219	FALSE	FALSE	Xanthomonadales	Xanthomonadaceae	Xanthomonas	FALSE
NC_008782	1437237	1437118	39	100	219	FALSE	FALSE	Burkholderiales	Comamonadaceae	Acidovorax	FALSE
NC_007508	2679919	2679794	41	100	218	FALSE	FALSE	Xanthomonadales	Xanthomonadaceae	Xanthomonas	FALSE
NC_010501	2608810	2608935	41	100	218	FALSE	FALSE	Pseudomonadales	Pseudomonadaceae	Pseudomonas	FALSE
NC_008782	1433695	1433808	37	100	215	FALSE	FALSE	Burkholderiales	Comamonadaceae	Acidovorax	FALSE
NC_007508	2649245	2649358	37	100	215	FALSE	FALSE	Xanthomonadales	Xanthomonadaceae	Xanthomonas	FALSE
NC_007508	2646765	2646890	41	100	213	FALSE	TRUE	Xanthomonadales	Xanthomonadaceae	Xanthomonas	FALSE
NC_008782	1431215	1431340	41	100	213	FALSE	TRUE	Burkholderiales	Comamonadaceae	Acidovorax	FALSE
NC_008782	1435721	1435837	38	100	212	FALSE	FALSE	Burkholderiales	Comamonadaceae	Acidovorax	FALSE
NC_007508	2651271	2651387	38	100	212	FALSE	FALSE	Xanthomonadales	Xanthomonadaceae	Xanthomonas	FALSE
NC_008752	3228439	3228549	36	100	211	FALSE	FALSE	Burkholderiales	Comamonadaceae	Acidovorax	FALSE
NC_008752	2975305	2975195	36	100	211	FALSE	FALSE	Burkholderiales	Comamonadaceae	Acidovorax	FALSE
NC_007508	2480343	2480453	36	100	211	FALSE	FALSE	Xanthomonadales	Xanthomonadaceae	Xanthomonas	FALSE
NC_007508	2740662	2740462	66	100	210	FALSE	FALSE	Xanthomonadales	Xanthomonadaceae	Xanthomonas	FALSE
NC_008782	1516132	1515932	66	100	210	FALSE	FALSE	Burkholderiales	Comamonadaceae	Acidovorax	FALSE
NC_007508	2684675	2684779	34	100	208	FALSE	FALSE	Xanthomonadales	Xanthomonadaceae	Xanthomonas	FALSE
NC_008782	1453741	1453845	34	100	208	FALSE	FALSE	Burkholderiales	Comamonadaceae	Acidovorax	FALSE
NC_007508	2713273	2713109	54	100	205	FALSE	FALSE	Xanthomonadales	Xanthomonadaceae	Xanthomonas	FALSE
NC_008782	1482342	1482178	54	100	205	FALSE	FALSE	Burkholderiales	Comamonadaceae	Acidovorax	FALSE
NC_008782	1482344	1482186	52	100	204	FALSE	FALSE	Burkholderiales	Comamonadaceae	Acidovorax	FALSE
NC_007508	2713275	2713117	52	100	204	FALSE	FALSE	Xanthomonadales	Xanthomonadaceae	Xanthomonas	FALSE
NC_008782	1438968	1438831	45	100	201	FALSE	FALSE	Burkholderiales	Comamonadaceae	Acidovorax	FALSE
NC_007508	2668513	2668650	45	100	201	FALSE	FALSE	Xanthomonadales	Xanthomonadaceae	Xanthomonas	FALSE
NC_007508	2740599	2740730	43	100	198	FALSE	FALSE	Xanthomonadales	Xanthomonadaceae	Xanthomonas	FALSE
NC_008782	1516069	1516200	43	100	198	FALSE	FALSE	Burkholderiales	Comamonadaceae	Acidovorax	FALSE
NC_010170	4538722	4538847	41	100	182	FALSE	FALSE	Burkholderiales	Alcaligenaceae	Bordetella	TRUE
NC_010002	4557677	4557552	41	100	182	FALSE	FALSE	Burkholderiales	Comamonadaceae	Delftia	TRUE
NC_009706	2720601	2720702	33	100	181	FALSE	TRUE	Clostridiales	Clostridiaceae	Clostridium	FALSE
NC_010320	1289592	1289491	33	100	181	FALSE	TRUE	Thermoanaerobacterales	Thermoanaerobacteraceae	Thermoanaerobacter	FALSE
NC_010002	4612728	4612588	46	100	180	FALSE	TRUE	Burkholderiales	Comamonadaceae	Delftia	FALSE
NC_010170	4482068	4482208	46	100	180	FALSE	TRUE	Burkholderiales	Alcaligenaceae	Bordetella	TRUE
NC_010170	4482180	4482043	45	100	174	FALSE	FALSE	Burkholderiales	Alcaligenaceae	Bordetella	TRUE
NC_010002	4612616	4612753	45	100	174	FALSE	FALSE	Burkholderiales	Comamonadaceae	Delftia	TRUE
NC_010002	4579130	4579318	62	100	171	FALSE	FALSE	Burkholderiales	Comamonadaceae	Delftia	FALSE
NC_010170	4517268	4517080	62	100	171	FALSE	FALSE	Burkholderiales	Alcaligenaceae	Bordetella	FALSE
NC_010170	4480011	4479895	38	100	169	FALSE	FALSE	Burkholderiales	Alcaligenaceae	Bordetella	FALSE
NC_010002	4614785	4614901	38	100	169	FALSE	FALSE	Burkholderiales	Comamonadaceae	Delftia	FALSE
NC_010002	4548459	4548584	41	100	166	FALSE	FALSE	Burkholderiales	Comamonadaceae	Delftia	FALSE
NC_010170	4559858	4559733	41	100	166	FALSE	FALSE	Burkholderiales	Alcaligenaceae	Bordetella	FALSE
NC_009227	207636	207773	45	100	157	FALSE	FALSE	Burkholderiales	Burkholderiaceae	Burkholderia	FALSE
NC_009779	10442	10305	45	100	157	FALSE	FALSE	Enterobacteriales	Enterobacteriaceae	Cronobacter	FALSE
NC_004757	2745941	2745840	33	100	156	FALSE	FALSE	Nitrosomonadales	Nitrosomonadaceae	Nitrosomonas	FALSE
NC_010087	356637	356536	33	100	156	FALSE	FALSE	Burkholderiales	Burkholderiaceae	Burkholderia	FALSE

NC_004757	2740408	2740551	47	100	154	FALSE	FALSE	Nitrosomonadales	Nitrosomonadaceae	Nitrosomonas	FALSE
NC_007947	1227423	1227280	47	100	154	FALSE	FALSE	Methylophilales	Methylophilaceae	Methylobacillus	FALSE
NC_004703	20139	20005	44	100	139	FALSE	FALSE	Bacteroidales	Bacteroidaceae	Bacteroides	FALSE
NC_009615	855180	855046	44	100	139	FALSE	FALSE	Bacteroidales	Porphyromonadaceae	Parabacteroides	FALSE
NC_004703	20103	20216	37	100	138	FALSE	FALSE	Bacteroidales	Bacteroidaceae	Bacteroides	FALSE
NC_009615	855144	855257	37	100	138	FALSE	FALSE	Bacteroidales	Porphyromonadaceae	Parabacteroides	FALSE
NC_004703	20191	20039	50	100	137	FALSE	FALSE	Bacteroidales	Bacteroidaceae	Bacteroides	FALSE
NC_009615	855232	855080	50	100	137	FALSE	FALSE	Bacteroidales	Porphyromonadaceae	Parabacteroides	FALSE
NC_004703	18997	19101	34	100	136	FALSE	TRUE	Bacteroidales	Bacteroidaceae	Bacteroides	FALSE
NC_009615	854038	854142	34	100	136	FALSE	TRUE	Bacteroidales	Porphyromonadaceae	Parabacteroides	TRUE
NC_004703	30864	31007	47	100	133	FALSE	FALSE	Bacteroidales	Bacteroidaceae	Bacteroides	FALSE
NC_009615	865766	865909	47	100	133	FALSE	FALSE	Bacteroidales	Porphyromonadaceae	Parabacteroides	FALSE
NC_008782	2368736	2368599	45	100	117	FALSE	FALSE	Burkholderiales	Comamonadaceae	Acidovorax	FALSE
NC_008825	2510375	2510512	45	100	117	FALSE	FALSE	Burkholderiales	Methylibium	Methylibium	FALSE
NC_010170	1106592	1106714	40	100	115	FALSE	FALSE	Burkholderiales	Alcaligenaceae	Bordetella	FALSE
NC_008782	1505419	1505297	40	100	115	FALSE	FALSE	Burkholderiales	Comamonadaceae	Acidovorax	FALSE
NC_008782	1378363	1378241	40	100	115	FALSE	FALSE	Burkholderiales	Comamonadaceae	Acidovorax	FALSE
NC_008023	1103315	1103482	55	100	114	FALSE	FALSE	Lactobacillales	Streptococcaceae	Streptococcus	FALSE
NC_008790	41148	40981	55	100	114	FALSE	FALSE	Campylobacterales	Campylobacteraceae	Campylobacter	FALSE
NC_008790	43432	43545	37	100	113	FALSE	FALSE	Campylobacterales	Campylobacteraceae	Campylobacter	FALSE
NC_008023	1101031	1100918	37	100	113	FALSE	FALSE	Lactobacillales	Streptococcaceae	Streptococcus	FALSE
NC_008752	2976616	2976768	50	99.02	202	FALSE	FALSE	Burkholderiales	Comamonadaceae	Acidovorax	FALSE
NC_008752	3227128	3226976	50	99.02	202	FALSE	FALSE	Burkholderiales	Comamonadaceae	Acidovorax	FALSE
NC_007508	2479032	2478877	51	99.02	202	FALSE	FALSE	Xanthomonadales	Xanthomonadaceae	Xanthomonas	FALSE
NC_004757	2745823	2746110	95	98.95	155	FALSE	FALSE	Nitrosomonadales	Nitrosomonadaceae	Nitrosomonas	FALSE
NC_010087	356519	356806	95	98.95	155	FALSE	FALSE	Burkholderiales	Burkholderiaceae	Burkholderia	FALSE
NC_007973	2508965	2508720	81	98.77	302	FALSE	FALSE	Burkholderiales	Burkholderiaceae	Cupriavidus	FALSE
NC_008782	1408064	1408309	81	98.77	302	FALSE	FALSE	Burkholderiales	Comamonadaceae	Acidovorax	FALSE
NC_010582	171391	171492	33	93.94	3	FALSE	FALSE	Lactobacillales	Streptococcaceae	Streptococcus	FALSE
NC_009443	894195	894094	33	100	3	FALSE	FALSE	Lactobacillales	Streptococcaceae	Streptococcus	FALSE
NC_009442	894488	894387	33	100	3	FALSE	FALSE	Lactobacillales	Streptococcaceae	Streptococcus	FALSE
NC_002758	442113	442012	33	100	3	FALSE	FALSE	Firmicutes	Bacillales	Staphylococcus	FALSE
NC_010380	1308273	1308172	33	93.94	3	FALSE	FALSE	Lactobacillales	Streptococcaceae	Streptococcus	FALSE
NC_009782	442116	442015	33	100	3	FALSE	FALSE	Firmicutes	Bacillales	Staphylococcus	FALSE
NC_004116	929703	929602	33	100	3	FALSE	FALSE	Lactobacillales	Streptococcaceae	Streptococcus	FALSE
NC_006449	783890	784054	54	98.15	63	FALSE	FALSE	Lactobacillales	Streptococcaceae	Streptococcus	FALSE
NC_006448	777475	777639	54	98.15	63	FALSE	FALSE	Lactobacillales	Streptococcaceae	Streptococcus	FALSE
NC_010080	1876801	1876637	54	98.15	63	FALSE	FALSE	Lactobacillales	Lactobacillaceae	Lactobacillus	FALSE
NC_008782	2365781	2365939	52	98.08	102	FALSE	FALSE	Burkholderiales	Comamonadaceae	Acidovorax	FALSE
NC_008825	2516661	2516503	52	98.08	102	FALSE	FALSE	Burkholderiales	Methylibium	Methylibium	FALSE
NC_007508	2740779	2740931	50	98	199	FALSE	FALSE	Xanthomonadales	Xanthomonadaceae	Xanthomonas	FALSE
NC_008782	1516249	1516401	50	98	199	FALSE	FALSE	Burkholderiales	Comamonadaceae	Acidovorax	FALSE
NC_008825	2510436	2510284	50	98	116	FALSE	FALSE	Burkholderiales	Methylibium	Methylibium	FALSE
NC_008782	2368675	2368827	50	98	116	FALSE	FALSE	Burkholderiales	Comamonadaceae	Acidovorax	FALSE
NC_008463	366330	366461	43	97.67	34	FALSE	FALSE	Pseudomonadales	Pseudomonadaceae	Pseudomonas	FALSE
NC_002516	352185	352316	43	97.67	34	FALSE	FALSE	Pseudomonadales	Pseudomonadaceae	Pseudomonas	FALSE
NC_008782	2234748	2234617	43	97.67	34	FALSE	FALSE	Burkholderiales	Comamonadaceae	Acidovorax	FALSE
NC_008782	2234553	2234684	43	97.67	33	FALSE	FALSE	Burkholderiales	Comamonadaceae	Acidovorax	FALSE
NC_008463	366525	366394	43	97.67	33	FALSE	FALSE	Pseudomonadales	Pseudomonadaceae	Pseudomonas	FALSE
NC_002516	352380	352249	43	97.67	33	FALSE	FALSE	Pseudomonadales	Pseudomonadaceae	Pseudomonas	FALSE
NC_009615	872929	873060	43	97.67	127	FALSE	FALSE	Bacteroidales	Porphyromonadaceae	Parabacteroides	FALSE
NC_004663	5236169	5236038	43	97.67	127	FALSE	FALSE	Bacteroidales	Bacteroidaceae	Bacteroides	FALSE
NC_008782	2365837	2365968	43	97.67	112	FALSE	FALSE	Burkholderiales	Comamonadaceae	Acidovorax	FALSE
NC_008825	2516605	2516474	43	97.67	112	FALSE	FALSE	Burkholderiales	Methylibium	Methylibium	FALSE

NC_008825	2573780	2573890	36	100	64	FALSE	FALSE	Burkholderiales	Methylibium	Methylibium	FALSE
NC_006513	1494066	1493965	33	92.93	64	FALSE	FALSE	Rhodocyclales	Rhodocyclaceae	Azoarcus	FALSE
NC_008782	2077237	2077347	36	100	64	FALSE	FALSE	Burkholderiales	Comamonadaceae	Acidovorax	FALSE
NC_007337	58083	58196	37	97.3	70	FALSE	FALSE	Burkholderiales	Burkholderiaceae	Cupriavidus	FALSE
NC_008766	23983	23870	37	97.3	70	FALSE	FALSE	Burkholderiales	Comamonadaceae	Acidovorax	FALSE
NC_008385	141	28	37	97.3	70	FALSE	FALSE	Burkholderiales	Burkholderiaceae	Burkholderia	FALSE
NC_008766	47880	47987	35	97.14	190	FALSE	FALSE	Burkholderiales	Comamonadaceae	Acidovorax	FALSE
NC_007337	5656	5549	35	97.14	190	FALSE	FALSE	Burkholderiales	Burkholderiaceae	Cupriavidus	FALSE
NC_010320	1317578	1317477	33	96.97	340	FALSE	TRUE	Thermoanaerobacterales	Thermoanaerobacteraceae	Thermoanaerobacter	FALSE
NC_010003	470498	470397	33	96.97	340	FALSE	TRUE	Thermotogales	Thermotogaceae	Petrotoga	FALSE
NC_004703	31603	31403	66	96.97	134	FALSE	FALSE	Bacteroidales	Bacteroidaceae	Bacteroides	FALSE
NC_009615	866505	866305	66	96.97	134	FALSE	FALSE	Bacteroidales	Porphyromonadaceae	Parabacteroides	FALSE
NC_008782	1377871	1377996	41	100	4	FALSE	FALSE	Burkholderiales	Comamonadaceae	Acidovorax	TRUE
NC_007508	2729566	2729691	41	100	4	FALSE	FALSE	Xanthomonadales	Xanthomonadaceae	Xanthomonas	TRUE
NC_007951	3570900	3570766	44	100	4	FALSE	FALSE	Burkholderiales	Burkholderiaceae	Burkholderia	TRUE
NC_010170	1505020	1504886	44	100	4	FALSE	FALSE	Burkholderiales	Alcaligenaceae	Bordetella	TRUE
NC_008782	1504927	1505052	41	100	4	FALSE	FALSE	Burkholderiales	Comamonadaceae	Acidovorax	TRUE
NC_002488	1694046	1694171	41	97.56	4	FALSE	FALSE	Xanthomonadales	Xanthomonadaceae	Xylella	TRUE
NC_009138	1993709	1993581	42	81.09	4	FALSE	FALSE	Burkholderiales	Oxalobacteraceae	Herminiimonas	FALSE
NC_004757	2738762	2738947	61	96.72	152	FALSE	FALSE	Nitrosomonadales	Nitrosomonadaceae	Nitrosomonas	FALSE
NC_007947	1229069	1228884	61	96.72	152	FALSE	FALSE	Methylophilales	Methylophilaceae	Methylobacillus	FALSE
NC_007508	2484792	2484613	59	96.61	217	FALSE	FALSE	Xanthomonadales	Xanthomonadaceae	Xanthomonas	FALSE
NC_008463	1316473	1316294	59	96.61	217	FALSE	FALSE	Pseudomonadales	Pseudomonadaceae	Pseudomonas	FALSE
NC_010170	1102192	1102371	59	96.61	104	FALSE	FALSE	Burkholderiales	Alcaligenaceae	Bordetella	FALSE
NC_008782	1509810	1509631	59	96.61	104	FALSE	FALSE	Burkholderiales	Comamonadaceae	Acidovorax	FALSE
NC_008782	1382754	1382575	59	96.61	104	FALSE	FALSE	Burkholderiales	Comamonadaceae	Acidovorax	FALSE
NC_010002	2998997	2998848	49	100	82	FALSE	FALSE	Burkholderiales	Comamonadaceae	Delftia	FALSE
NC_007973	3283974	3283825	49	100	82	FALSE	FALSE	Burkholderiales	Burkholderiaceae	Cupriavidus	FALSE
NC_008782	3107643	3107494	49	89.8	82	FALSE	FALSE	Burkholderiales	Comamonadaceae	Acidovorax	FALSE
NC_004757	2745797	2745967	56	96.43	150	FALSE	FALSE	Nitrosomonadales	Nitrosomonadaceae	Nitrosomonas	FALSE
NC_010087	356493	356663	56	96.43	150	FALSE	FALSE	Burkholderiales	Burkholderiaceae	Burkholderia	FALSE
NC_007947	1225292	1225474	60	88.33	61	FALSE	FALSE	Methylophilales	Methylophilaceae	Methylobacillus	FALSE
NC_010087	356813	356631	60	100	61	FALSE	FALSE	Burkholderiales	Burkholderiaceae	Burkholderia	FALSE
NC_004757	2746117	2745935	60	100	61	FALSE	FALSE	Nitrosomonadales	Nitrosomonadaceae	Nitrosomonas	FALSE
NC_010002	2998867	2999010	47	100	78	TRUE	FALSE	Burkholderiales	Comamonadaceae	Delftia	FALSE
NC_008782	3107513	3107656	47	87.23	78	TRUE	FALSE	Burkholderiales	Comamonadaceae	Acidovorax	FALSE
NC_007973	3283844	3283987	47	100	78	TRUE	FALSE	Burkholderiales	Burkholderiaceae	Cupriavidus	FALSE
NC_007973	1371985	1371869	38	86.84	291	FALSE	FALSE	Burkholderiales	Burkholderiaceae	Cupriavidus	FALSE
NC_010002	3000699	3000815	38	100	291	FALSE	FALSE	Burkholderiales	Comamonadaceae	Delftia	FALSE
NC_007973	3285676	3285792	38	100	291	FALSE	FALSE	Burkholderiales	Burkholderiaceae	Cupriavidus	FALSE
NC_009615	3946257	3946382	41	97.56	125	TRUE	FALSE	Bacteroidales	Porphyromonadaceae	Parabacteroides	FALSE
NC_009615	2629587	2629712	41	90.24	125	TRUE	FALSE	Bacteroidales	Porphyromonadaceae	Parabacteroides	FALSE
NC_004663	1423467	1423592	41	97.56	125	TRUE	FALSE	Bacteroidales	Bacteroidaceae	Bacteroides	FALSE
NC_008505	33942	34064	40	95	361	FALSE	TRUE	Lactobacillales	Streptococcaceae	Lactococcus	FALSE
NC_010469	10283	10161	40	95	361	FALSE	TRUE	Firmicutes	Lactobacillales	Leuconostoc	FALSE
NC_008740	3579120	3579010	36	95	344	FALSE	FALSE	Alteromonadales	Alteromonadaceae	Marinobacter	FALSE
NC_008260	735551	735673	40	95	344	FALSE	FALSE	Oceanospirillales	Alcanivoracaceae	Alcanivorax	FALSE
NC_007973	2509502	2509960	152	94.74	294	FALSE	FALSE	Burkholderiales	Burkholderiaceae	Cupriavidus	FALSE
NC_008782	1389116	1388706	136	94.74	294	FALSE	FALSE	Burkholderiales	Comamonadaceae	Acidovorax	FALSE
NC_010084	2518133	2518020	37	94.59	220	FALSE	FALSE	Burkholderiales	Burkholderiaceae	Burkholderia	FALSE
NC_007519	3378569	3378456	37	94.59	220	FALSE	FALSE	Desulfovibrionales	Desulfovibrionaceae	Desulfovibrio	FALSE
NC_004663	1417550	1417651	33	96.97	130	FALSE	FALSE	Bacteroidales	Bacteroidaceae	Bacteroides	FALSE
NC_009615	2623606	2623707	33	96.97	130	FALSE	FALSE	Bacteroidales	Porphyromonadaceae	Parabacteroides	FALSE
NC_009615	3940317	3940418	33	87.88	130	FALSE	FALSE	Bacteroidales	Porphyromonadaceae	Parabacteroides	FALSE

NC_010170	1102391	1102164	75	96	79	FALSE	FALSE	Burkholderiales	Alcaligenaceae	Bordetella	FALSE
NC_008782	1509611	1509838	75	96	79	FALSE	FALSE	Burkholderiales	Comamonadaceae	Acidovorax	FALSE
NC_007973	2516114	2515887	75	86.67	79	FALSE	FALSE	Burkholderiales	Burkholderiaceae	Cupriavidus	FALSE
NC_008782	1382555	1382782	75	96	79	FALSE	FALSE	Burkholderiales	Comamonadaceae	Acidovorax	FALSE
NC_004663	1419962	1420153	63	92.4	128	TRUE	TRUE	Bacteroidales	Bacteroidaceae	Bacteroides	FALSE
NC_009615	2626292	2626495	67	92.4	128	TRUE	TRUE	Bacteroidales	Porphyromonadaceae	Parabacteroides	FALSE
NC_009615	3942961	3943164	67	92.4	128	TRUE	TRUE	Bacteroidales	Porphyromonadaceae	Parabacteroides	FALSE
NC_008463	2705884	2705753	43	92.24	76	FALSE	FALSE	Pseudomonadales	Pseudomonadaceae	Pseudomonas	FALSE
NC_007973	1376736	1376867	43	93.02	76	FALSE	FALSE	Burkholderiales	Burkholderiaceae	Cupriavidus	FALSE
NC_007973	1605707	1605847	46	92.24	76	FALSE	FALSE	Burkholderiales	Burkholderiaceae	Cupriavidus	FALSE
NC_010170	2267855	2267983	42	91.78	76	FALSE	FALSE	Burkholderiales	Alcaligenaceae	Bordetella	FALSE
NC_010320	1317472	1317591	39	92.31	341	FALSE	FALSE	Thermoanaerobacterales	Thermoanaerobacteraceae	Thermoanaerobacter	FALSE
NC_010003	470392	470511	39	92.31	341	FALSE	FALSE	Thermotogales	Thermotogaceae	Petrotoga	FALSE
NC_007947	1229154	1228999	51	92.16	147	FALSE	FALSE	Methylophilales	Methylophilaceae	Methylobacillus	FALSE
NC_004757	2738677	2738832	51	92.16	147	FALSE	FALSE	Nitrosomonadales	Nitrosomonadaceae	Nitrosomonas	FALSE
NC_007973	1367423	1367533	36	97.22	84	FALSE	FALSE	Burkholderiales	Burkholderiaceae	Cupriavidus	FALSE
NC_008786	4113476	4113345	43	81.65	84	FALSE	FALSE	Burkholderiales	Comamonadaceae	Verminephrobacter	FALSE
NC_008463	2714048	2713938	36	97.22	84	FALSE	FALSE	Pseudomonadales	Pseudomonadaceae	Pseudomonas	FALSE
NC_010002	488793	488912	39	79.49	86	FALSE	FALSE	Burkholderiales	Comamonadaceae	Delftia	FALSE
NC_008782	3105162	3105281	39	87.18	86	FALSE	FALSE	Burkholderiales	Comamonadaceae	Acidovorax	FALSE
NC_010002	2991145	2991264	39	100	86	FALSE	FALSE	Burkholderiales	Comamonadaceae	Delftia	FALSE
NC_007973	3281508	3281627	39	100	86	FALSE	FALSE	Burkholderiales	Burkholderiaceae	Cupriavidus	FALSE
NC_009656	124233	124364	43	91.78	378	FALSE	FALSE	Pseudomonadales	Pseudomonadaceae	Pseudomonas	FALSE
NC_009659	3145237	3145365	42	91.31	378	FALSE	FALSE	Burkholderiales	Oxalobacteraceae	Janthinobacterium	FALSE
NC_009138	2031231	2031344	37	91	269	FALSE	FALSE	Burkholderiales	Oxalobacteraceae	Herminiimonas	FALSE
NC_009719	3664251	3664364	37	91.89	269	FALSE	FALSE	Rhizobiales	Phyllobacteriaceae	Parvibaculum	FALSE
NC_010080	1876475	1876609	44	84.09	10	FALSE	FALSE	Lactobacillales	Lactobacillaceae	Lactobacillus	FALSE
NC_006448	777801	777667	44	93.18	10	FALSE	FALSE	Lactobacillales	Streptococcaceae	Streptococcus	FALSE
NC_006449	784216	784082	44	93.18	10	FALSE	FALSE	Lactobacillales	Streptococcaceae	Streptococcus	FALSE
NC_008054	1127864	1127730	44	93.18	10	FALSE	FALSE	Lactobacillales	Lactobacillaceae	Lactobacillus	FALSE
NC_008529	1147194	1147060	44	93.18	10	FALSE	FALSE	Lactobacillales	Lactobacillaceae	Lactobacillus	FALSE
NC_006448	1474679	1474861	60	92.57	11	FALSE	FALSE	Lactobacillales	Streptococcaceae	Streptococcus	FALSE
NC_006449	1482428	1482610	60	92.57	11	FALSE	FALSE	Lactobacillales	Streptococcaceae	Streptococcus	FALSE
NC_010467	16507	16325	60	88	11	FALSE	FALSE	Lactobacillales	Leuconostocaceae	Leuconostoc	FALSE
NC_010469	2589	2404	61	90.25	11	FALSE	FALSE	Lactobacillales	Leuconostocaceae	Leuconostoc	FALSE
NC_008496	30440	30625	61	92.57	11	FALSE	FALSE	Lactobacillales	Leuconostocaceae	Leuconostoc	FALSE
NC_007973	1603430	1603534	34	91.18	304	FALSE	TRUE	Burkholderiales	Burkholderiaceae	Cupriavidus	FALSE
NC_010170	2265576	2265680	34	91.18	304	FALSE	TRUE	Burkholderiales	Alcaligenaceae	Bordetella	FALSE
NC_010170	2265578	2265700	40	95	80	FALSE	FALSE	Burkholderiales	Alcaligenaceae	Bordetella	FALSE
NC_010002	491239	491111	42	82.98	80	FALSE	FALSE	Burkholderiales	Comamonadaceae	Delftia	FALSE
NC_007973	1603432	1603554	40	95	80	FALSE	FALSE	Burkholderiales	Burkholderiaceae	Cupriavidus	FALSE
NC_007947	1216506	1216640	44	90.91	237	FALSE	FALSE	Methylophilales	Methylophilaceae	Methylobacillus	FALSE
NC_010084	2498817	2498683	44	90.91	237	FALSE	FALSE	Burkholderiales	Burkholderiaceae	Burkholderia	FALSE
NC_009656	3884843	3884739	34	97.06	20	FALSE	FALSE	Pseudomonadales	Pseudomonadaceae	Pseudomonas	FALSE
NC_008786	4117962	4117858	34	79.41	20	FALSE	FALSE	Burkholderiales	Comamonadaceae	Verminephrobacter	FALSE
NC_006526	1984114	1984218	34	88.24	20	FALSE	FALSE	Sphingomonadales	Sphingomonadaceae	Zymomonas	FALSE
NC_003919	2606470	2606574	34	97.06	20	FALSE	FALSE	Xanthomonadales	Xanthomonadaceae	Xanthomonas	FALSE
NC_010170	4517089	4517217	42	100	87	FALSE	FALSE	Burkholderiales	Alcaligenaceae	Bordetella	FALSE
NC_010002	4579309	4579181	42	100	87	FALSE	FALSE	Burkholderiales	Comamonadaceae	Delftia	FALSE
NC_009138	2037452	2037324	42	71	87	FALSE	FALSE	Burkholderiales	Oxalobacteraceae	Herminiimonas	FALSE
NC_010087	356779	356576	67	82.09	145	FALSE	FALSE	Burkholderiales	Burkholderiaceae	Burkholderia	FALSE
NC_004757	2746083	2745880	67	98.51	145	FALSE	FALSE	Nitrosomonadales	Nitrosomonadaceae	Nitrosomonas	FALSE
NC_008463	1316884	1317093	69	89.86	207	FALSE	FALSE	Pseudomonadales	Pseudomonadaceae	Pseudomonas	FALSE
NC_007508	2485203	2485412	69	89.86	207	FALSE	FALSE	Xanthomonadales	Xanthomonadaceae	Xanthomonas	FALSE

NC_007973	3284554	3284414	46	100	77	FALSE	FALSE	Burkholderiales	Burkholderiaceae	Cupriavidus	FALSE
NC_008782	3108222	3108082	46	69.57	77	FALSE	FALSE	Burkholderiales	Comamonadaceae	Acidovorax	FALSE
NC_010002	2999577	2999437	46	100	77	FALSE	FALSE	Burkholderiales	Comamonadaceae	Delftia	FALSE
NC_008782	2312565	2312684	39	89.74	66	FALSE	FALSE	Burkholderiales	Comamonadaceae	Acidovorax	FALSE
NC_008825	2561066	2560947	39	89.74	66	FALSE	FALSE	Burkholderiales	Methylibium	Methylibium	FALSE
NC_006513	1513649	1513768	39	89.74	66	FALSE	FALSE	Rhodocyclales	Rhodocyclaceae	Azoarcus	FALSE
NC_002488	1704737	1704582	51	67.33	31	FALSE	FALSE	Xanthomonadales	Xanthomonadaceae	Xylella	FALSE
NC_008782	1515876	1515724	50	100	31	FALSE	FALSE	Burkholderiales	Comamonadaceae	Acidovorax	FALSE
NC_007508	2740406	2740254	50	100	31	FALSE	FALSE	Xanthomonadales	Xanthomonadaceae	Xanthomonas	FALSE
NC_009348	1862221	1862102	39	89.74	46	FALSE	FALSE	Aeromonadales	Aeromonadaceae	Aeromonas	FALSE
NC_008570	1984688	1984569	39	87.18	46	FALSE	FALSE	Aeromonadales	Aeromonadaceae	Aeromonas	FALSE
NC_004347	1042039	1041920	39	89.74	46	FALSE	FALSE	Alteromonadales	Shewanellaceae	Shewanella	FALSE
NC_010170	1587892	1588191	99	88.89	250	FALSE	TRUE	Burkholderiales	Alcaligenaceae	Bordetella	FALSE
NC_007951	3656290	3656589	99	88.89	250	FALSE	TRUE	Burkholderiales	Burkholderiaceae	Burkholderia	FALSE
NC_008782	1382867	1382667	66	86.36	105	FALSE	FALSE	Burkholderiales	Comamonadaceae	Acidovorax	FALSE
NC_010170	1102079	1102279	66	93.94	105	FALSE	FALSE	Burkholderiales	Alcaligenaceae	Bordetella	FALSE
NC_008782	1509923	1509723	66	86.36	105	FALSE	FALSE	Burkholderiales	Comamonadaceae	Acidovorax	FALSE
NC_010080	1876587	1876471	38	81.58	88	FALSE	TRUE	Lactobacillales	Lactobacillaceae	Lactobacillus	FALSE
NC_008054	1127752	1127868	38	92.11	88	FALSE	TRUE	Lactobacillales	Lactobacillaceae	Lactobacillus	FALSE
NC_008529	1147082	1147198	38	92.11	88	FALSE	TRUE	Lactobacillales	Lactobacillaceae	Lactobacillus	FALSE
NC_007973	1376916	1376797	39	87.18	75	FALSE	FALSE	Burkholderiales	Burkholderiaceae	Cupriavidus	FALSE
NC_008463	2705704	2705823	39	89.74	75	FALSE	FALSE	Pseudomonadales	Pseudomonadaceae	Pseudomonas	FALSE
NC_010170	2268032	2267913	39	89.74	75	FALSE	FALSE	Burkholderiales	Alcaligenaceae	Bordetella	FALSE
NC_007973	1605887	1605768	39	87.18	75	FALSE	FALSE	Burkholderiales	Burkholderiaceae	Cupriavidus	FALSE
NC_007337	9554	9396	52	88.46	191	FALSE	FALSE	Burkholderiales	Burkholderiaceae	Cupriavidus	FALSE
NC_008766	43989	44147	52	88.46	191	FALSE	FALSE	Burkholderiales	Comamonadaceae	Acidovorax	FALSE
NC_002488	1704586	1704729	47	64.81	32	FALSE	FALSE	Xanthomonadales	Xanthomonadaceae	Xylella	TRUE
NC_007508	2740258	2740416	52	100	32	FALSE	FALSE	Xanthomonadales	Xanthomonadaceae	Xanthomonas	TRUE
NC_008782	1515728	1515886	52	100	32	FALSE	FALSE	Burkholderiales	Comamonadaceae	Acidovorax	TRUE
NC_002947	741575	741730	51	88.24	101	FALSE	FALSE	Pseudomonadales	Pseudomonadaceae	Pseudomonas	FALSE
NC_002947	3595762	3595607	51	88.24	101	FALSE	FALSE	Pseudomonadales	Pseudomonadaceae	Pseudomonas	FALSE
NC_008782	2355771	2355616	51	88.24	101	FALSE	FALSE	Burkholderiales	Comamonadaceae	Acidovorax	FALSE
NC_002947	1819911	1820066	51	88.24	101	FALSE	FALSE	Pseudomonadales	Pseudomonadaceae	Pseudomonas	FALSE
NC_002947	1429638	1429793	51	88.24	101	FALSE	FALSE	Pseudomonadales	Pseudomonadaceae	Pseudomonas	FALSE
NC_002947	2069301	2069456	51	88.24	101	FALSE	FALSE	Pseudomonadales	Pseudomonadaceae	Pseudomonas	FALSE
NC_002947	1425778	1425933	51	88.24	101	FALSE	FALSE	Pseudomonadales	Pseudomonadaceae	Pseudomonas	FALSE
NC_002947	4393335	4393180	51	88.24	101	FALSE	FALSE	Pseudomonadales	Pseudomonadaceae	Pseudomonas	FALSE
NC_002947	4348956	4348801	51	88.24	101	FALSE	FALSE	Pseudomonadales	Pseudomonadaceae	Pseudomonas	FALSE
NC_009720	913964	914068	34	88	107	FALSE	FALSE	Rhizobiales	Xanthobacteraceae	Xanthobacter	FALSE
NC_009668	464457	464353	34	88.24	107	FALSE	FALSE	Rhizobiales	Brucellaceae	Ochrobactrum	FALSE
NC_009614	1003715	1003602	37	79.47	311	FALSE	FALSE	Bacteroidales	Bacteroidaceae	Bacteroides	FALSE
NC_009614	1988529	1988639	36	91.67	311	FALSE	FALSE	Bacteroidales	Bacteroidaceae	Bacteroides	FALSE
NC_009615	4738733	4738623	36	91.67	311	FALSE	FALSE	Bacteroidales	Porphyromonadaceae	Parabacteroides	FALSE
NC_002947	1820084	1819914	56	91.07	39	FALSE	FALSE	Pseudomonadales	Pseudomonadaceae	Pseudomonas	FALSE
NC_002947	2069474	2069304	56	91.07	39	FALSE	FALSE	Pseudomonadales	Pseudomonadaceae	Pseudomonas	FALSE
NC_002947	741748	741578	56	91.07	39	FALSE	FALSE	Pseudomonadales	Pseudomonadaceae	Pseudomonas	FALSE
NC_002947	4393162	4393332	56	91.07	39	FALSE	FALSE	Pseudomonadales	Pseudomonadaceae	Pseudomonas	FALSE
NC_002947	3595589	3595759	56	91.07	39	FALSE	FALSE	Pseudomonadales	Pseudomonadaceae	Pseudomonas	FALSE
NC_010322	1828957	1829127	56	71.43	39	FALSE	FALSE	Pseudomonadales	Pseudomonadaceae	Pseudomonas	FALSE
NC_008782	2355598	2355768	56	71.43	39	FALSE	FALSE	Burkholderiales	Comamonadaceae	Acidovorax	FALSE
NC_002947	4348783	4348953	56	91.07	39	FALSE	FALSE	Pseudomonadales	Pseudomonadaceae	Pseudomonas	FALSE
NC_002947	1429811	1429641	56	91.07	39	FALSE	FALSE	Pseudomonadales	Pseudomonadaceae	Pseudomonas	FALSE
NC_002947	1425951	1425781	56	91.07	39	FALSE	FALSE	Pseudomonadales	Pseudomonadaceae	Pseudomonas	FALSE
NC_010170	1102431	1102015	138	86.23	111	FALSE	FALSE	Burkholderiales	Alcaligenaceae	Bordetella	TRUE

NC_008782	1509571	1509987	138	86.23	111	FALSE	FALSE	Burkholderiales	Comamonadaceae	Acidovorax	TRUE
NC_008782	1382515	1382931	138	86.23	111	FALSE	FALSE	Burkholderiales	Comamonadaceae	Acidovorax	TRUE
NC_006513	1470113	1470247	44	77.74	65	FALSE	FALSE	Rhodocyclales	Rhodocyclaceae	Azoarcus	FALSE
NC_008782	2362381	2362506	41	90.24	65	FALSE	FALSE	Burkholderiales	Comamonadaceae	Acidovorax	FALSE
NC_008825	2530439	2530314	41	90.24	65	FALSE	FALSE	Burkholderiales	Methylibium	Methylibium	FALSE
NC_007951	3656348	3656088	86	86.05	256	FALSE	FALSE	Burkholderiales	Burkholderiaceae	Burkholderia	FALSE
NC_010170	1587950	1587690	86	86.05	256	FALSE	FALSE	Burkholderiales	Alcaligenaceae	Bordetella	FALSE
NC_009085	796917	796690	75	85.12	159	FALSE	FALSE	Pseudomonadales	Moraxellaceae	Acinetobacter	FALSE
NC_009345	2510	2764	84	85.81	159	FALSE	FALSE	Enterobacteriales	Enterobacteriaceae	Shigella	FALSE
NC_004663	1417621	1417514	35	91.26	131	FALSE	FALSE	Bacteroidales	Bacteroidaceae	Bacteroides	FALSE
NC_009615	2623677	2623576	33	91.26	131	FALSE	FALSE	Bacteroidales	Porphyromonadaceae	Parabacteroides	FALSE
NC_009615	3940388	3940287	33	73.59	131	FALSE	FALSE	Bacteroidales	Porphyromonadaceae	Parabacteroides	FALSE
NC_010087	338991	339095	34	85.29	353	FALSE	FALSE	Burkholderiales	Burkholderiaceae	Burkholderia	FALSE
NC_009138	2049015	2049119	34	85	353	FALSE	FALSE	Burkholderiales	Oxalobacteraceae	Herminiimonas	FALSE
NC_008782	2699747	2699905	52	68.03	71	FALSE	FALSE	Burkholderiales	Comamonadaceae	Acidovorax	FALSE
NC_007508	2485392	2485219	57	92.98	71	FALSE	FALSE	Xanthomonadales	Xanthomonadaceae	Xanthomonas	FALSE
NC_008463	1317073	1316900	57	92.98	71	FALSE	FALSE	Pseudomonadales	Pseudomonadaceae	Pseudomonas	FALSE
NC_009092	274346	274447	33	84	158	FALSE	TRUE	Alteromonadales	Shewanellaceae	Shewanella	FALSE
NC_005139	149811	149912	33	84.85	158	FALSE	TRUE	Vibrionales	Vibrionaceae	Vibrio	FALSE
NC_007973	1603524	1603420	34	87.06	81	FALSE	FALSE	Burkholderiales	Burkholderiaceae	Cupriavidus	FALSE
NC_010002	491147	491251	34	78.9	81	FALSE	FALSE	Burkholderiales	Comamonadaceae	Delftia	FALSE
NC_010170	2265688	2265566	40	87.06	81	FALSE	FALSE	Burkholderiales	Alcaligenaceae	Bordetella	FALSE
NC_010410	3654452	3654703	83	84	366	FALSE	TRUE	Pseudomonadales	Moraxellaceae	Acinetobacter	FALSE
NC_009651	33802	34053	83	84.34	366	FALSE	TRUE	Enterobacteriales	Enterobacteriaceae	Klebsiella	FALSE
NC_007086	1231149	1231018	43	93.02	18	FALSE	FALSE	Xanthomonadales	Xanthomonadaceae	Xanthomonas	FALSE
NC_004578	4813797	4813666	43	93.02	18	FALSE	FALSE	Pseudomonadales	Pseudomonadaceae	Pseudomonas	FALSE
NC_003902	3728371	3728502	43	93.02	18	FALSE	FALSE	Xanthomonadales	Xanthomonadaceae	Xanthomonas	FALSE
NC_006513	1384010	1383876	44	57.48	18	FALSE	FALSE	Rhodocyclales	Rhodocyclaceae	Azoarcus	FALSE
NC_010376	1004300	1004419	39	84.03	336	FALSE	FALSE	Clostridiales	Clostridiales Family XI. Incertae Sedis	Finegoldia	FALSE
NC_008024	1664936	1664829	35	84.03	336	FALSE	FALSE	Lactobacillales	Streptococcaceae	Streptococcus	FALSE
NC_009656	3880711	3880836	41	82.93	175	FALSE	FALSE	Pseudomonadales	Pseudomonadaceae	Pseudomonas	FALSE
NC_006526	1987161	1987036	41	82.93	175	FALSE	FALSE	Sphingomonadales	Sphingomonadaceae	Zymomonas	FALSE
NC_002947	4393439	4393329	36	84.95	38	FALSE	FALSE	Pseudomonadales	Pseudomonadaceae	Pseudomonas	FALSE
NC_002947	3595887	3595756	43	80.45	38	FALSE	FALSE	Pseudomonadales	Pseudomonadaceae	Pseudomonas	TRUE
NC_002947	741453	741581	42	81.34	38	FALSE	FALSE	Pseudomonadales	Pseudomonadaceae	Pseudomonas	FALSE
NC_002947	4349060	4348950	36	84.95	38	FALSE	FALSE	Pseudomonadales	Pseudomonadaceae	Pseudomonas	FALSE
NC_002947	1819786	1819917	43	77.94	38	FALSE	FALSE	Pseudomonadales	Pseudomonadaceae	Pseudomonas	FALSE
NC_004578	4249659	4249546	37	86.49	38	FALSE	FALSE	Pseudomonadales	Pseudomonadaceae	Pseudomonas	FALSE
NC_002947	2069197	2069307	36	84.95	38	FALSE	FALSE	Pseudomonadales	Pseudomonadaceae	Pseudomonas	FALSE
NC_008782	2355878	2355765	37	81.34	38	FALSE	FALSE	Burkholderiales	Comamonadaceae	Acidovorax	FALSE
NC_002947	1429534	1429644	36	82.21	38	FALSE	FALSE	Pseudomonadales	Pseudomonadaceae	Pseudomonas	FALSE
NC_010170	1587693	1587920	75	82.67	253	FALSE	FALSE	Burkholderiales	Alcaligenaceae	Bordetella	FALSE
NC_007951	3656091	3656318	75	82.67	253	FALSE	FALSE	Burkholderiales	Burkholderiaceae	Burkholderia	FALSE
NC_007973	3285230	3284970	86	82.56	299	FALSE	FALSE	Burkholderiales	Burkholderiaceae	Cupriavidus	FALSE
NC_010002	3000253	2999993	86	82.56	299	FALSE	FALSE	Burkholderiales	Comamonadaceae	Delftia	FALSE
NC_009228	76450	76578	42	80.95	19	FALSE	FALSE	Burkholderiales	Burkholderiaceae	Burkholderia	FALSE
NC_006834	2911833	2911705	42	80.95	19	FALSE	FALSE	Xanthomonadales	Xanthomonadaceae	Xanthomonas	FALSE
NC_003919	2839804	2839676	42	80.95	19	FALSE	FALSE	Xanthomonadales	Xanthomonadaceae	Xanthomonas	FALSE
NC_007705	2889973	2889845	42	80.95	19	FALSE	FALSE	Xanthomonadales	Xanthomonadaceae	Xanthomonas	FALSE
NC_004703	22103	21963	46	80.43	140	FALSE	TRUE	Bacteroidales	Bacteroidaceae	Bacteroides	FALSE
NC_009615	857144	857004	46	80.43	140	FALSE	TRUE	Bacteroidales	Porphyromonadaceae	Parabacteroides	FALSE
NC_004578	3629304	3629101	67	79.55	124	FALSE	FALSE	Pseudomonadales	Pseudomonadaceae	Pseudomonas	FALSE
NC_010943	1508366	1508539	57	79.55	124	FALSE	FALSE	Xanthomonadales	Xanthomonadaceae	Stenotrophomonas	FALSE
NC_007947	1227305	1227084	73	79.45	153	FALSE	FALSE	Methylophilales	Methylophilaceae	Methylobacillus	FALSE

NC_004757	2740526	2740747	73	79.45	153	FALSE	FALSE	Nitrosomonadales	Nitrosomonadaceae	Nitrosomonas	FALSE
NC_009614	1001791	1001892	33	79.27	308	TRUE	FALSE	Bacteroidales	Bacteroidaceae	Bacteroides	FALSE
NC_009615	4736803	4736919	38	79.27	308	TRUE	FALSE	Bacteroidales	Porphyromonadaceae	Parabacteroides	FALSE
NC_009437	956530	956372	52	78.85	118	FALSE	FALSE	Thermoanaerobacterales	Thermoanaerobacterales	Caldicellulosiruptor	FALSE
NC_003869	2625406	2625564	52	78.85	118	FALSE	FALSE	Thermoanaerobacterales	Thermoanaerobacteraceae	Thermoanaerobacter	FALSE
NC_010337	2869102	2868947	51	78.56	164	FALSE	FALSE	Clostridiales	Heliobacteriaceae	Heliobacterium	FALSE
NC_009437	522102	522242	46	78.56	164	FALSE	FALSE	Thermoanaerobacterales	Thermoanaerobacterales	Caldicellulosiruptor	FALSE
NC_009719	3626032	3626199	55	78.18	322	FALSE	FALSE	Rhizobiales	Phyllobacteriaceae	Parvibaculum	FALSE
NC_007973	2520322	2520489	55	78.18	322	FALSE	FALSE	Burkholderiales	Burkholderiaceae	Cupriavidus	FALSE
NC_010002	2986504	2986611	35	77.14	295	FALSE	TRUE	Burkholderiales	Comamonadaceae	Delftia	FALSE
NC_007973	3276886	3276993	35	77.14	295	FALSE	TRUE	Burkholderiales	Burkholderiaceae	Cupriavidus	FALSE
NC_009434	1830069	1830176	35	76.79	222	FALSE	FALSE	Pseudomonadales	Pseudomonadaceae	Pseudomonas	FALSE
NC_010627	380956	381081	41	76.79	222	FALSE	FALSE	Burkholderiales	Burkholderiaceae	Burkholderia	FALSE
NC_007973	1603335	1603466	43	76.74	305	FALSE	FALSE	Burkholderiales	Burkholderiaceae	Cupriavidus	FALSE
NC_010002	491336	491205	43	76.74	305	FALSE	FALSE	Burkholderiales	Comamonadaceae	Delftia	FALSE
NC_005241	23836	23976	46	78.26	62	FALSE	FALSE	Burkholderiales	Burkholderiaceae	Cupriavidus	FALSE
NC_010627	381023	381172	49	71.65	62	FALSE	FALSE	Burkholderiales	Burkholderiaceae	Burkholderia	FALSE
NC_009434	1830118	1830258	46	78.26	62	FALSE	FALSE	Pseudomonadales	Pseudomonadaceae	Pseudomonas	TRUE
NC_008782	3076332	3076207	41	85.37	73	FALSE	FALSE	Burkholderiales	Comamonadaceae	Acidovorax	FALSE
NC_010528	2165885	2165769	38	81.13	73	FALSE	FALSE	Burkholderiales	Burkholderiaceae	Cupriavidus	FALSE
NC_007651	926304	926429	41	60.98	73	FALSE	FALSE	Burkholderiales	Burkholderiaceae	Burkholderia	FALSE
NC_008463	2708165	2708040	41	82.93	360	FALSE	FALSE	Pseudomonadales	Pseudomonadaceae	Pseudomonas	FALSE
NC_008752	745152	745277	41	68.29	360	FALSE	FALSE	Burkholderiales	Comamonadaceae	Acidovorax	FALSE
NC_010939	631257	631373	38	75.34	59	FALSE	FALSE	Pasteurellales	Pasteurellaceae	Actinobacillus	FALSE
NC_010278	564246	564362	38	75.34	59	FALSE	FALSE	Pasteurellales	Pasteurellaceae	Actinobacillus	FALSE
NC_009053	599798	599914	38	75.34	59	FALSE	FALSE	Pasteurellales	Pasteurellaceae	Actinobacillus	FALSE
NC_003888	4626376	4626224	50	76	110	FALSE	FALSE	Streptomycineae	Streptomycetaceae	Streptomyces	FALSE
NC_003155	7182867	7183025	52	74.54	110	FALSE	FALSE	Streptomycineae	Streptomycetaceae	Streptomyces	FALSE
NC_006582	46283	46170	37	75.06	178	FALSE	FALSE	Bacillales	Bacillaceae	Bacillus	FALSE
NC_009641	1956773	1956880	35	75.06	178	FALSE	FALSE	Firmicutes	Bacillales	Staphylococcus	FALSE
NC_010002	3007395	3007541	48	75	300	FALSE	FALSE	Burkholderiales	Comamonadaceae	Delftia	FALSE
NC_007973	3292372	3292518	48	75	300	FALSE	FALSE	Burkholderiales	Burkholderiaceae	Cupriavidus	FALSE
NC_009615	861893	861762	43	74.42	135	TRUE	FALSE	Bacteroidales	Porphyromonadaceae	Parabacteroides	FALSE
NC_004703	26876	26745	43	74.42	135	TRUE	FALSE	Bacteroidales	Bacteroidaceae	Bacteroides	FALSE
NC_010170	1493873	1494109	78	73.67	258	TRUE	FALSE	Burkholderiales	Alcaligenaceae	Bordetella	FALSE
NC_007951	3559753	3559989	78	73.67	258	TRUE	FALSE	Burkholderiales	Burkholderiaceae	Burkholderia	FALSE
NC_009052	2808072	2808209	45	73.33	170	FALSE	FALSE	Alteromonadales	Shewanellaceae	Shewanella	FALSE
NC_009052	2137904	2137767	45	73.33	170	FALSE	FALSE	Alteromonadales	Shewanellaceae	Shewanella	FALSE
NC_006371	186570	186433	45	73.33	170	FALSE	FALSE	Vibrionales	Vibrionaceae	Photobacterium	FALSE
NC_007953	1242919	1242785	44	72.73	270	FALSE	FALSE	Burkholderiales	Burkholderiaceae	Burkholderia	FALSE
NC_008757	14904	14770	44	72.73	270	FALSE	FALSE	Burkholderiales	Comamonadaceae	Polaromonas	FALSE
NC_004459	2555623	2555504	39	72.73	123	TRUE	FALSE	Vibrionales	Vibrionaceae	Vibrio	FALSE
NC_009997	2710227	2710126	33	72.73	123	TRUE	FALSE	Alteromonadales	Shewanellaceae	Shewanella	FALSE
NC_008463	2690721	2690593	42	72.3	301	FALSE	TRUE	Pseudomonadales	Pseudomonadaceae	Pseudomonas	FALSE
NC_007973	1669883	1670008	41	72.3	301	FALSE	TRUE	Burkholderiales	Burkholderiaceae	Cupriavidus	FALSE
NC_007973	1367151	1367020	43	72.09	317	FALSE	FALSE	Burkholderiales	Burkholderiaceae	Cupriavidus	TRUE
NC_008786	4113818	4113949	43	72.09	317	FALSE	FALSE	Burkholderiales	Comamonadaceae	Verminephrobacter	FALSE
NC_007973	2509945	2509793	50	72	285	FALSE	FALSE	Burkholderiales	Burkholderiaceae	Cupriavidus	FALSE
NC_008782	1388721	1388873	50	72	285	FALSE	FALSE	Burkholderiales	Comamonadaceae	Acidovorax	FALSE
NC_009436	2904548	2904435	37	71.66	163	FALSE	FALSE	Enterobacteriales	Enterobacteriaceae	Enterobacter	TRUE
NC_010943	559060	558959	33	71.66	163	FALSE	FALSE	Xanthomonadales	Xanthomonadaceae	Stenotrophomonas	FALSE
NC_008782	1516357	1516169	62	71.25	200	TRUE	FALSE	Burkholderiales	Comamonadaceae	Acidovorax	FALSE
NC_007508	2740887	2740699	62	71.25	200	TRUE	FALSE	Xanthomonadales	Xanthomonadaceae	Xanthomonas	FALSE
NC_006526	1987006	1987119	37	71.25	176	FALSE	FALSE	Sphingomonadales	Sphingomonadaceae	Zymomonas	FALSE

NC_009656	3880863	3880753	36	71.25	176	FALSE	FALSE	Pseudomonadales	Pseudomonadaceae	Pseudomonas	FALSE
NC_003888	4626360	4626205	51	72.55	43	FALSE	FALSE	Streptomycineae	Streptomycetaceae	Streptomyces	FALSE
NC_009142	4965150	4965305	51	66.67	43	FALSE	FALSE	Pseudonocardineae	Pseudonocardiaceae	Saccharopolyspora	FALSE
NC_003155	7182883	7183038	51	74.51	43	FALSE	FALSE	Streptomycineae	Streptomycetaceae	Streptomyces	FALSE
NC_007951	3566270	3566082	62	71.24	247	FALSE	FALSE	Burkholderiales	Burkholderiaceae	Burkholderia	FALSE
NC_010170	1500390	1500202	62	71.24	247	FALSE	FALSE	Burkholderiales	Alcaligenaceae	Bordetella	FALSE
NC_010170	1588178	1587999	59	71.19	251	FALSE	FALSE	Burkholderiales	Alcaligenaceae	Bordetella	FALSE
NC_007951	3656576	3656397	59	71.19	251	FALSE	FALSE	Burkholderiales	Burkholderiaceae	Burkholderia	FALSE
NC_007973	2526666	2526824	52	71.15	283	FALSE	TRUE	Burkholderiales	Burkholderiaceae	Cupriavidus	FALSE
NC_009719	3632382	3632540	52	71.15	283	FALSE	TRUE	Rhizobiales	Phyllobacteriaceae	Parvibaculum	FALSE
NC_007508	2478793	2478930	45	71.11	203	FALSE	FALSE	Xanthomonadales	Xanthomonadaceae	Xanthomonas	FALSE
NC_008752	3226892	3227029	45	71.11	203	FALSE	FALSE	Burkholderiales	Comamonadaceae	Acidovorax	FALSE
NC_008752	2976852	2976715	45	71.11	203	FALSE	FALSE	Burkholderiales	Comamonadaceae	Acidovorax	FALSE
NC_008463	2708101	2708238	45	80	89	FALSE	FALSE	Pseudomonadales	Pseudomonadaceae	Pseudomonas	FALSE
NC_008390	2180104	2179952	50	52.78	89	FALSE	FALSE	Burkholderiales	Burkholderiaceae	Burkholderia	FALSE
NC_008752	745216	745079	45	80	89	FALSE	FALSE	Burkholderiales	Comamonadaceae	Acidovorax	FALSE
NC_007951	3559737	3559603	44	70.45	266	FALSE	FALSE	Burkholderiales	Burkholderiaceae	Burkholderia	FALSE
NC_010170	1493857	1493723	44	70.45	266	FALSE	FALSE	Burkholderiales	Alcaligenaceae	Bordetella	FALSE
NC_009142	1233192	1233079	37	70.27	37	FALSE	FALSE	Pseudonocardineae	Pseudonocardiaceae	Saccharopolyspora	FALSE
NC_002944	3519853	3519966	37	67.57	37	FALSE	FALSE	Corynebacterineae	Mycobacteriaceae	Mycobacterium	FALSE
NC_010397	3513406	3513519	37	70.27	37	FALSE	FALSE	Corynebacterineae	Mycobacteriaceae	Mycobacterium	FALSE
NC_008344	679468	679250	72	69.44	350	FALSE	FALSE	Nitrosomonadales	Nitrosomonadaceae	Nitrosomonas	FALSE
NC_009085	792559	792777	72	69	350	FALSE	FALSE	Pseudomonadales	Moraxellaceae	Acinetobacter	FALSE
NC_008344	2385792	2386010	72	69.44	350	FALSE	FALSE	Nitrosomonadales	Nitrosomonadaceae	Nitrosomonas	FALSE
NC_007973	2526852	2526733	39	69.23	284	FALSE	FALSE	Burkholderiales	Burkholderiaceae	Cupriavidus	FALSE
NC_009719	3632568	3632449	39	69.23	284	FALSE	FALSE	Rhizobiales	Phyllobacteriaceae	Parvibaculum	FALSE
NC_010002	4544494	4544327	55	69.09	323	FALSE	FALSE	Burkholderiales	Comamonadaceae	Delftia	FALSE
NC_010170	4563822	4563989	55	69.09	323	FALSE	FALSE	Burkholderiales	Alcaligenaceae	Bordetella	FALSE
NC_002488	1638759	1638881	40	90	8	FALSE	FALSE	Xanthomonadales	Xanthomonadaceae	Xylella	FALSE
NC_007508	2631762	2631884	40	59.03	8	FALSE	FALSE	Xanthomonadales	Xanthomonadaceae	Xanthomonas	FALSE
NC_007951	3682213	3682091	40	64.03	8	FALSE	FALSE	Burkholderiales	Burkholderiaceae	Burkholderia	FALSE
NC_009138	2066099	2065977	40	90	8	FALSE	FALSE	Burkholderiales	Oxalobacteraceae	Herminiimonas	FALSE
NC_010170	1595611	1595489	40	64.03	8	FALSE	FALSE	Burkholderiales	Alcaligenaceae	Bordetella	FALSE
NC_010170	1339445	1339323	40	43.53	8	FALSE	FALSE	Burkholderiales	Alcaligenaceae	Bordetella	FALSE
NC_007641	12371	12264	35	68.61	22	FALSE	FALSE	Rhodospirillales	Rhodospirillaceae	Rhodospirillum	FALSE
NC_010104	351794	351678	38	68.61	22	FALSE	FALSE	Rhizobiales	Brucellaceae	Brucella	FALSE
NC_010167	353060	352938	40	66.96	22	FALSE	FALSE	Rhizobiales	Brucellaceae	Brucella	FALSE
NC_004311	351865	351749	38	68.61	22	FALSE	FALSE	Rhizobiales	Brucellaceae	Brucella	FALSE
NC_010814	305196	305053	47	68.09	373	FALSE	FALSE	Desulfuromonadales	Geobacteraceae	Geobacter	FALSE
NC_008609	266532	266389	47	68.09	373	FALSE	FALSE	Desulfuromonadales	Pelobacteraceae	Pelobacter	FALSE
NC_007705	3245083	3244928	51	67.9	179	FALSE	FALSE	Xanthomonadales	Xanthomonadaceae	Xanthomonas	FALSE
NC_006834	3245432	3245277	51	67.9	179	FALSE	FALSE	Xanthomonadales	Xanthomonadaceae	Xanthomonas	FALSE
NC_003276	382898	383005	35	67.62	44	FALSE	TRUE	Nostocales	Nostocaceae	Nostoc	FALSE
NC_010296	594835	594945	36	67.62	44	FALSE	TRUE	Cyanobacteria	Chroococcales	Microcystis	FALSE
NC_007410	363912	363805	35	67.62	44	FALSE	TRUE	Nostocales	Nostocaceae	Anabaena	FALSE
NC_003919	2675251	2675397	48	67.38	121	FALSE	FALSE	Xanthomonadales	Xanthomonadaceae	Xanthomonas	FALSE
NC_008782	2710897	2711040	47	67.38	121	FALSE	FALSE	Burkholderiales	Comamonadaceae	Acidovorax	FALSE
NC_006511	2889731	2889847	38	66.87	172	FALSE	FALSE	Enterobacteriales	Enterobacteriaceae	Salmonella	FALSE
NC_009439	4138037	4138141	34	66.87	172	FALSE	FALSE	Pseudomonadales	Pseudomonadaceae	Pseudomonas	FALSE
NC_009784	1883103	1882990	37	67.57	74	FALSE	FALSE	Vibrionales	Vibrionaceae	Vibrio	FALSE
NC_009783	3010341	3010228	37	67	74	FALSE	FALSE	Vibrionales	Vibrionaceae	Vibrio	FALSE
NC_009784	1339892	1339779	37	67.57	74	FALSE	FALSE	Vibrionales	Vibrionaceae	Vibrio	FALSE
NC_009901	3790553	3790666	37	64	74	FALSE	FALSE	Alteromonadales	Shewanellaceae	Shewanella	FALSE
NC_009784	1975602	1975489	37	67.57	74	FALSE	FALSE	Vibrionales	Vibrionaceae	Vibrio	FALSE

NC_009719	3615749	3615525	74	66.33	196	FALSE	FALSE	Rhizobiales	Phyllobacteriaceae	Parvibaculum	TRUE
NC_009138	1982976	1982734	80	65.76	196	FALSE	FALSE	Burkholderiales	Oxalobacteraceae	Herminiimonas	FALSE
NC_009659	3087561	3087722	53	54	151	FALSE	FALSE	Burkholderiales	Oxalobacteraceae	Janthinobacterium	FALSE
NC_004757	2779620	2779459	53	77.36	151	FALSE	FALSE	Nitrosomonadales	Nitrosomonadaceae	Nitrosomonas	FALSE
NC_008782	1505412	1505074	112	65.65	108	FALSE	TRUE	Burkholderiales	Comamonadaceae	Acidovorax	FALSE
NC_010170	1106599	1106937	112	65.65	108	FALSE	TRUE	Burkholderiales	Alcaligenaceae	Bordetella	FALSE
NC_008752	739876	739679	65	66.15	40	FALSE	FALSE	Burkholderiales	Comamonadaceae	Acidovorax	FALSE
NC_010084	2532123	2531926	65	66.15	40	FALSE	FALSE	Burkholderiales	Burkholderiaceae	Burkholderia	FALSE
NC_003295	2819894	2820094	66	63.52	40	FALSE	FALSE	Burkholderiales	Burkholderiaceae	Ralstonia	FALSE
NC_010688	2405468	2405283	61	59.94	5	FALSE	FALSE	Xanthomonadales	Xanthomonadaceae	Xanthomonas	FALSE
NC_010170	1339290	1339439	49	24.63	5	FALSE	FALSE	Burkholderiales	Alcaligenaceae	Bordetella	FALSE
NC_007508	2631917	2631732	61	96.72	5	FALSE	FALSE	Xanthomonadales	Xanthomonadaceae	Xanthomonas	FALSE
NC_007951	3682058	3682243	61	96.72	5	FALSE	FALSE	Burkholderiales	Burkholderiaceae	Burkholderia	FALSE
NC_007508	2593698	2593513	61	58.36	5	FALSE	FALSE	Xanthomonadales	Xanthomonadaceae	Xanthomonas	FALSE
NC_009719	3711916	3712116	66	59.94	5	FALSE	FALSE	Rhizobiales	Phyllobacteriaceae	Parvibaculum	FALSE
NC_003902	2496667	2496852	61	59.94	5	FALSE	FALSE	Xanthomonadales	Xanthomonadaceae	Xanthomonas	FALSE
NC_009085	788587	788372	71	64.54	56	FALSE	TRUE	Pseudomonadales	Moraxellaceae	Acinetobacter	FALSE
NC_004741	2609978	2610193	71	64.79	56	FALSE	TRUE	Enterobacteriales	Enterobacteriaceae	Shigella	FALSE
NC_008344	683443	683658	71	64.79	56	FALSE	TRUE	Nitrosomonadales	Nitrosomonadaceae	Nitrosomonas	FALSE
NC_008344	2381817	2381602	71	64.79	56	FALSE	TRUE	Nitrosomonadales	Nitrosomonadaceae	Nitrosomonas	FALSE
NC_007644	1558919	1558698	73	64.37	227	FALSE	FALSE	Thermoanaerobacterales	Thermoanaerobacteraceae	Moorella	FALSE
NC_009454	1231458	1231709	83	64.37	227	FALSE	FALSE	Clostridiales	Peptococcaceae	Pelotomaculum	FALSE
NC_007406	3387945	3388100	51	64.08	192	FALSE	FALSE	Rhizobiales	Bradyrhizobiaceae	Nitrobacter	TRUE
NC_009720	4395951	4395793	52	64.08	192	FALSE	FALSE	Rhizobiales	Xanthobacteraceae	Xanthobacter	FALSE
NC_007973	1362064	1361921	47	72.34	15	FALSE	FALSE	Burkholderiales	Burkholderiaceae	Cupriavidus	FALSE
NC_009656	3880995	3881144	49	60.44	15	FALSE	FALSE	Pseudomonadales	Pseudomonadaceae	Pseudomonas	TRUE
NC_008786	4114496	4114630	44	59.41	15	FALSE	FALSE	Burkholderiales	Comamonadaceae	Verminephrobacter	FALSE
NC_010084	2531051	2530917	44	61.61	15	FALSE	FALSE	Burkholderiales	Burkholderiaceae	Burkholderia	FALSE
NC_008782	2805266	2805123	47	65.96	15	FALSE	FALSE	Burkholderiales	Comamonadaceae	Acidovorax	TRUE
NC_002488	1691840	1691664	58	74.14	17	FALSE	FALSE	Xanthomonadales	Xanthomonadaceae	Xylella	FALSE
NC_010170	4555876	4556055	59	58.12	17	FALSE	FALSE	Burkholderiales	Alcaligenaceae	Bordetella	FALSE
NC_004113	1684262	1684038	74	47.67	17	FALSE	FALSE	Cyanobacteria	Chroococcales	Thermosynechococcus	TRUE
NC_004113	246006	246230	74	47.67	17	FALSE	FALSE	Cyanobacteria	Chroococcales	Thermosynechococcus	TRUE
NC_010170	1122635	1122456	59	58.12	17	FALSE	FALSE	Burkholderiales	Alcaligenaceae	Bordetella	FALSE
NC_010170	4551208	4551369	53	74.02	17	FALSE	FALSE	Burkholderiales	Alcaligenaceae	Bordetella	FALSE
NC_010170	1126284	1126108	58	75.86	17	FALSE	FALSE	Burkholderiales	Alcaligenaceae	Bordetella	FALSE
NC_008782	1494325	1494501	58	75.86	17	FALSE	FALSE	Burkholderiales	Comamonadaceae	Acidovorax	FALSE
NC_009663	244601	244762	53	52.34	223	TRUE	FALSE	Proteobacteria	Epsilonproteobacteria	Sulfurovum	TRUE
NC_007575	2066169	2066333	54	74.77	223	TRUE	FALSE	Campylobacterales	Helicobacteraceae	Sulfurimonas	TRUE
NC_007947	1228857	1229021	54	62.96	146	FALSE	FALSE	Methylophilales	Methylophilaceae	Methylobacillus	TRUE
NC_004757	2738974	2738810	54	62.96	146	FALSE	FALSE	Nitrosomonadales	Nitrosomonadaceae	Nitrosomonas	TRUE
NC_009434	3453655	3453837	60	62.73	97	FALSE	FALSE	Pseudomonadales	Pseudomonadaceae	Pseudomonas	FALSE
NC_002927	982015	982191	58	62.73	97	FALSE	FALSE	Burkholderiales	Alcaligenaceae	Bordetella	FALSE
NC_009439	3844849	3844706	47	62.61	356	FALSE	FALSE	Pseudomonadales	Pseudomonadaceae	Pseudomonas	FALSE
NC_008390	2215617	2215447	56	62.61	356	FALSE	FALSE	Burkholderiales	Burkholderiaceae	Burkholderia	TRUE
NC_008782	1431313	1431200	37	62.16	214	FALSE	FALSE	Burkholderiales	Comamonadaceae	Acidovorax	FALSE
NC_007508	2646863	2646750	37	62.16	214	FALSE	FALSE	Xanthomonadales	Xanthomonadaceae	Xanthomonas	FALSE
NC_007508	2740634	2740810	58	62.07	197	FALSE	FALSE	Xanthomonadales	Xanthomonadaceae	Xanthomonas	FALSE
NC_008782	1516104	1516280	58	62.07	197	FALSE	FALSE	Burkholderiales	Comamonadaceae	Acidovorax	FALSE
NC_002570	2908808	2908942	44	62.08	95	FALSE	FALSE	Bacillales	Bacillaceae	Bacillus	TRUE
NC_009633	2545320	2545189	43	61.8	95	FALSE	FALSE	Clostridiales	Clostridiaceae	Alkaliphilus	TRUE
NC_010170	4563529	4563762	77	61.57	335	FALSE	FALSE	Burkholderiales	Alcaligenaceae	Bordetella	FALSE
NC_010002	4544787	4544554	77	61.57	335	FALSE	FALSE	Burkholderiales	Comamonadaceae	Delftia	FALSE
NC_007947	1228898	1229194	98	60.42	148	TRUE	FALSE	Methylophilales	Methylophilaceae	Methylobacillus	FALSE

NC_004757	2738933	2738637	98	61.92	148	TRUE	FALSE	Nitrosomonadales	Nitrosomonadaceae	Nitrosomonas	FALSE
NC_010170	2264531	2264322	69	49.13	331	FALSE	FALSE	Burkholderiales	Alcaligenaceae	Bordetella	FALSE
NC_007973	1602375	1602166	69	72.46	331	FALSE	FALSE	Burkholderiales	Burkholderiaceae	Cupriavidus	FALSE
NC_004310	1354476	1354634	52	62.82	21	FALSE	FALSE	Rhizobiales	Brucellaceae	Brucella	FALSE
NC_009720	1621660	1621842	60	53.09	21	FALSE	FALSE	Rhizobiales	Xanthobacteraceae	Xanthobacter	FALSE
NC_003317	633702	633544	52	64.4	21	FALSE	FALSE	Rhizobiales	Brucellaceae	Brucella	FALSE
NC_006932	1372577	1372735	52	62.82	21	FALSE	FALSE	Rhizobiales	Brucellaceae	Brucella	FALSE
NC_009659	3083833	3083576	85	53.76	351	FALSE	FALSE	Burkholderiales	Oxalobacteraceae	Janthinobacterium	TRUE
NC_008344	2525013	2524786	75	67.76	351	FALSE	FALSE	Nitrosomonadales	Nitrosomonadaceae	Nitrosomonas	TRUE
NC_010581	3995246	3995022	74	60.72	358	FALSE	FALSE	Rhizobiales	Beijerinckiaceae	Beijerinckia	TRUE
NC_008435	2345777	2345992	71	60.72	358	FALSE	FALSE	Rhizobiales	Bradyrhizobiaceae	Rhodopseudomonas	TRUE
NC_009651	34001	33846	51	60.78	221	TRUE	FALSE	Enterobacteriales	Enterobacteriaceae	Klebsiella	FALSE
NC_010410	3654651	3654496	51	60	221	TRUE	FALSE	Pseudomonadales	Moraxellaceae	Acinetobacter	FALSE
NC_010998	232555	232421	44	60.39	232	TRUE	TRUE	Rhizobiales	Rhizobiaceae	Rhizobium	FALSE
NC_007765	301841	301695	48	59.8	232	TRUE	TRUE	Rhizobiales	Rhizobiaceae	Rhizobium	FALSE
NC_002939	1526255	1526115	46	59.95	99	FALSE	FALSE	Desulfuromonadales	Geobacteraceae	Geobacter	FALSE
NC_008609	2524802	2524957	51	59.95	99	FALSE	FALSE	Desulfuromonadales	Pelobacteraceae	Pelobacter	FALSE
NC_009719	3626441	3626668	75	59.59	276	FALSE	TRUE	Rhizobiales	Phyllobacteriaceae	Parvibaculum	FALSE
NC_007973	2520731	2520958	75	59.59	276	FALSE	TRUE	Burkholderiales	Burkholderiaceae	Cupriavidus	FALSE
NC_009434	1829735	1829866	43	59.56	160	TRUE	FALSE	Pseudomonadales	Pseudomonadaceae	Pseudomonas	FALSE
NC_010627	380641	380766	41	59.56	160	TRUE	FALSE	Burkholderiales	Burkholderiaceae	Burkholderia	FALSE
NC_010397	4107708	4107595	37	59.46	347	FALSE	FALSE	Corynebacterineae	Mycobacteriaceae	Mycobacterium	FALSE
NC_008268	2194424	2194311	37	59.46	347	FALSE	FALSE	Corynebacterineae	Nocardiaceae	Rhodococcus	FALSE
NC_010170	1096044	1096247	67	63.71	72	FALSE	FALSE	Burkholderiales	Alcaligenaceae	Bordetella	FALSE
NC_009719	3615581	3615787	68	57.12	72	FALSE	FALSE	Rhizobiales	Phyllobacteriaceae	Parvibaculum	FALSE
NC_009138	1982760	1982990	76	56.94	72	FALSE	FALSE	Burkholderiales	Oxalobacteraceae	Herminiimonas	FALSE
NC_008782	1505387	1505049	112	58.58	106	FALSE	FALSE	Burkholderiales	Comamonadaceae	Acidovorax	FALSE
NC_010170	1106624	1106962	112	59.49	106	FALSE	FALSE	Burkholderiales	Alcaligenaceae	Bordetella	FALSE
NC_010688	2849715	2849578	45	71.11	26	FALSE	FALSE	Xanthomonadales	Xanthomonadaceae	Xanthomonas	FALSE
NC_007086	2443792	2443929	45	71.11	26	FALSE	FALSE	Xanthomonadales	Xanthomonadaceae	Xanthomonas	FALSE
NC_009719	3664392	3664255	45	45.98	26	FALSE	FALSE	Rhizobiales	Phyllobacteriaceae	Parvibaculum	FALSE
NC_009138	2031372	2031235	45	47.86	26	FALSE	FALSE	Burkholderiales	Oxalobacteraceae	Herminiimonas	FALSE
NC_008345	1965876	1966028	50	56	338	FALSE	TRUE	Alteromonadales	Shewanellaceae	Shewanella	TRUE
NC_008228	2288687	2288839	50	62	338	FALSE	TRUE	Alteromonadales	Pseudoalteromonadaceae	Pseudoalteromonas	TRUE
NC_008782	2311857	2311976	39	58.59	370	FALSE	FALSE	Burkholderiales	Comamonadaceae	Acidovorax	FALSE
NC_008825	2561771	2561652	39	58.59	370	FALSE	FALSE	Burkholderiales	Methylibium	Methylibium	FALSE
NC_009012	3382076	3382312	78	58.24	177	FALSE	FALSE	Clostridiales	Clostridiaceae	Clostridium	TRUE
NC_006582	1306023	1306265	80	58.24	177	FALSE	FALSE	Bacillales	Bacillaceae	Bacillus	TRUE
NC_007973	2520901	2520569	110	58.18	325	FALSE	FALSE	Burkholderiales	Burkholderiaceae	Cupriavidus	FALSE
NC_009719	3626611	3626279	110	58.18	325	FALSE	FALSE	Rhizobiales	Phyllobacteriaceae	Parvibaculum	FALSE
NC_007086	2477023	2476859	54	61.11	68	FALSE	FALSE	Xanthomonadales	Xanthomonadaceae	Xanthomonas	FALSE
NC_007508	2621271	2621107	54	55.56	68	FALSE	FALSE	Xanthomonadales	Xanthomonadaceae	Xanthomonas	FALSE
NC_010688	2808756	2808920	54	57.41	68	FALSE	FALSE	Xanthomonadales	Xanthomonadaceae	Xanthomonas	FALSE
NC_007274	6313	5999	104	57.48	122	FALSE	TRUE	Pseudomonadales	Pseudomonadaceae	Pseudomonas	TRUE
NC_007274	63083	62769	104	57.48	122	FALSE	TRUE	Pseudomonadales	Pseudomonadaceae	Pseudomonas	TRUE
NC_003919	4143032	4143328	98	57.48	122	FALSE	TRUE	Xanthomonadales	Xanthomonadaceae	Xanthomonas	FALSE
NC_008752	747659	747477	60	56.84	93	FALSE	FALSE	Burkholderiales	Comamonadaceae	Acidovorax	FALSE
NC_003295	2808918	2809100	60	56.97	93	FALSE	FALSE	Burkholderiales	Burkholderiaceae	Ralstonia	FALSE
NC_009615	206181	206381	66	56.78	129	TRUE	FALSE	Bacteroidales	Porphyromonadaceae	Parabacteroides	TRUE
NC_004663	2072871	2073056	61	56.78	129	TRUE	FALSE	Bacteroidales	Bacteroidaceae	Bacteroides	FALSE
NC_008609	1280696	1280899	67	56.29	372	FALSE	FALSE	Desulfuromonadales	Pelobacteraceae	Pelobacter	FALSE
NC_010815	13562	13738	58	56.29	372	FALSE	FALSE	Desulfuromonadales	Geobacteraceae	Geobacter	FALSE
NC_007973	1366877	1367092	71	34.06	316	FALSE	FALSE	Burkholderiales	Burkholderiaceae	Cupriavidus	FALSE
NC_008786	4114062	4113877	61	77.72	316	FALSE	FALSE	Burkholderiales	Comamonadaceae	Verminephrobacter	FALSE

NC_007955	1752904	1753032	42	55.8	275	FALSE	FALSE	Methanosarcinales	Methanosarcinaceae	Methanococcoides	FALSE
NC_009464	2425919	2426065	48	55.8	275	FALSE	FALSE	Archaea	Euryarchaeota	environmental	FALSE
NC_004663	72995	73144	49	55.61	126	FALSE	FALSE	Bacteroidales	Bacteroidaceae	Bacteroides	FALSE
NC_010729	626182	626364	60	55.61	126	FALSE	FALSE	Bacteroidales	Porphyromonadaceae	Porphyromonas	TRUE
NC_010545	888335	888186	49	55.51	229	FALSE	FALSE	Corynebacterineae	Corynebacteriaceae	Corynebacterium	FALSE
NC_009656	3872666	3872517	49	55.51	229	FALSE	FALSE	Pseudomonadales	Pseudomonadaceae	Pseudomonas	FALSE
NC_010170	1502235	1502384	49	55.37	239	FALSE	FALSE	Burkholderiales	Alcaligenaceae	Bordetella	FALSE
NC_007951	3568115	3568264	49	55.37	239	FALSE	FALSE	Burkholderiales	Burkholderiaceae	Burkholderia	FALSE
NC_009719	3626025	3626288	87	60.41	85	FALSE	FALSE	Rhizobiales	Phyllobacteriaceae	Parvibaculum	FALSE
NC_009138	1993243	1993464	73	44.37	85	FALSE	FALSE	Burkholderiales	Oxalobacteraceae	Herminiimonas	FALSE
NC_007973	2520315	2520578	87	60.41	85	FALSE	FALSE	Burkholderiales	Burkholderiaceae	Cupriavidus	FALSE
NC_009438	4641856	4642053	65	54.97	377	TRUE	TRUE	Alteromonadales	Shewanellaceae	Shewanella	TRUE
NC_008740	625883	625683	66	54.97	377	TRUE	TRUE	Alteromonadales	Alteromonadaceae	Marinobacter	TRUE
NC_010002	4544310	4544660	116	54.78	319	FALSE	FALSE	Burkholderiales	Comamonadaceae	Delftia	FALSE
NC_010170	4564006	4563656	116	54.78	319	FALSE	FALSE	Burkholderiales	Alcaligenaceae	Bordetella	FALSE
NC_004741	2605818	2606021	67	52.23	58	TRUE	FALSE	Enterobacteriales	Enterobacteriaceae	Shigella	TRUE
NC_008344	679279	679482	67	52.23	58	TRUE	FALSE	Nitrosomonadales	Nitrosomonadaceae	Nitrosomonas	TRUE
NC_009085	792748	792545	67	57.06	58	TRUE	FALSE	Pseudomonadales	Moraxellaceae	Acinetobacter	TRUE
NC_008344	2385981	2385778	67	52.23	58	TRUE	FALSE	Nitrosomonadales	Nitrosomonadaceae	Nitrosomonas	TRUE
NC_007164	774254	774102	50	53.08	0	FALSE	FALSE	Corynebacterineae	Corynebacteriaceae	Corynebacterium	FALSE
NC_008609	2527086	2526925	53	48.75	0	FALSE	FALSE	Desulfuromonadales	Pelobacteraceae	Pelobacter	FALSE
NC_008609	2525518	2525339	59	56.67	0	FALSE	FALSE	Desulfuromonadales	Pelobacteraceae	Pelobacter	FALSE
NC_008609	2526888	2526742	48	60.42	0	FALSE	FALSE	Desulfuromonadales	Pelobacteraceae	Pelobacter	FALSE
NC_008554	3491402	3491548	48	56.25	0	FALSE	FALSE	Syntrophobacterales	Syntrophobacteraceae	Syntrophobacter	FALSE
NC_008554	3490426	3490572	48	52.08	0	FALSE	FALSE	Syntrophobacterales	Syntrophobacteraceae	Syntrophobacter	FALSE
NC_007164	776200	776054	48	58.33	0	FALSE	FALSE	Corynebacterineae	Corynebacteriaceae	Corynebacterium	FALSE
NC_007164	776261	776115	48	58.33	0	FALSE	FALSE	Corynebacterineae	Corynebacteriaceae	Corynebacterium	FALSE
NC_007164	774187	774041	48	58.33	0	FALSE	FALSE	Corynebacterineae	Corynebacteriaceae	Corynebacterium	FALSE
NC_007164	774553	774407	48	48.36	0	FALSE	FALSE	Corynebacterineae	Corynebacteriaceae	Corynebacterium	FALSE
NC_009778	2778195	2778049	48	45.04	0	FALSE	FALSE	Enterobacteriales	Enterobacteriaceae	Cronobacter	FALSE
NC_003197	3077386	3077207	59	47.23	0	FALSE	FALSE	Enterobacteriales	Enterobacteriaceae	Salmonella	FALSE
NC_007164	774919	774773	48	52.08	0	FALSE	FALSE	Corynebacterineae	Corynebacteriaceae	Corynebacterium	FALSE
NC_007164	775102	774956	48	56.25	0	FALSE	FALSE	Corynebacterineae	Corynebacteriaceae	Corynebacterium	FALSE
NC_007164	775651	775505	48	56.25	0	FALSE	FALSE	Corynebacterineae	Corynebacteriaceae	Corynebacterium	FALSE
NC_007164	775956	775810	48	58.33	0	FALSE	FALSE	Corynebacterineae	Corynebacteriaceae	Corynebacterium	FALSE
NC_010125	2254079	2253933	48	58.33	0	FALSE	FALSE	Rhodospirillales	Acetobacteraceae	Gluconacetobacter	FALSE
NC_003197	3094490	3094344	48	54.17	0	FALSE	FALSE	Enterobacteriales	Enterobacteriaceae	Salmonella	FALSE
NC_009439	4138929	4138783	48	54.17	0	FALSE	FALSE	Pseudomonadales	Pseudomonadaceae	Pseudomonas	FALSE
NC_009439	4139008	4138844	54	54.21	0	FALSE	FALSE	Pseudomonadales	Pseudomonadaceae	Pseudomonas	FALSE
NC_010545	2291571	2291425	48	58.33	0	FALSE	FALSE	Corynebacterineae	Corynebacteriaceae	Corynebacterium	FALSE
NC_007164	776017	775871	48	51.81	0	FALSE	FALSE	Corynebacterineae	Corynebacteriaceae	Corynebacterium	FALSE
NC_007164	776752	776603	49	50.6	0	FALSE	FALSE	Corynebacterineae	Corynebacteriaceae	Corynebacterium	FALSE
NC_007164	776932	776786	48	58.33	0	FALSE	FALSE	Corynebacterineae	Corynebacteriaceae	Corynebacterium	FALSE
NC_007164	776993	776847	48	60.42	0	FALSE	FALSE	Corynebacterineae	Corynebacteriaceae	Corynebacterium	FALSE
NC_010545	2289558	2289412	48	56.25	0	FALSE	FALSE	Corynebacterineae	Corynebacteriaceae	Corynebacterium	FALSE
NC_008709	1949280	1949426	48	43.45	0	FALSE	FALSE	Alteromonadales	Psychromonadaceae	Psychromonas	FALSE
NC_009483	995209	995376	55	46.82	0	FALSE	FALSE	Desulfuromonadales	Geobacteraceae	Geobacter	FALSE
NC_008554	3490914	3491060	48	52.08	0	FALSE	FALSE	Syntrophobacterales	Syntrophobacteraceae	Syntrophobacter	FALSE
NC_010545	2290595	2290449	48	51.16	0	FALSE	FALSE	Corynebacterineae	Corynebacteriaceae	Corynebacterium	FALSE
NC_010545	2290778	2290632	48	54.17	0	FALSE	FALSE	Corynebacterineae	Corynebacteriaceae	Corynebacterium	FALSE
NC_008554	3491497	3491670	57	49.89	0	FALSE	FALSE	Syntrophobacterales	Syntrophobacteraceae	Syntrophobacter	FALSE
NC_009943	3591862	3592023	53	49.63	0	FALSE	FALSE	Desulfobacterales	Desulfobacteraceae	Desulfococcus	FALSE
NC_009943	3591938	3592084	48	50.72	0	FALSE	FALSE	Desulfobacterales	Desulfobacteraceae	Desulfococcus	FALSE
NC_006905	3049698	3049552	48	52.08	0	FALSE	FALSE	Enterobacteriales	Enterobacteriaceae	Salmonella	FALSE

NC_010545	2291815	2291669	48	54.17	0	FALSE	FALSE	Corynebacterineae	Corynebacteriaceae	Corynebacterium	FALSE
NC_010545	2292182	2292036	48	60.42	0	FALSE	FALSE	Corynebacterineae	Corynebacteriaceae	Corynebacterium	FALSE
NC_009468	35330	35506	58	51.72	0	FALSE	FALSE	Rhodospirillales	Acetobacteraceae	Acidiphilium	FALSE
NC_009778	2778500	2778354	48	56	0	FALSE	FALSE	Enterobacteriales	Enterobacteriaceae	Cronobacter	FALSE
NC_009468	37312	37458	48	53.13	0	FALSE	FALSE	Rhodospirillales	Acetobacteraceae	Acidiphilium	FALSE
NC_009778	2807381	2807223	52	50	0	FALSE	FALSE	Enterobacteriales	Enterobacteriaceae	Cronobacter	FALSE
NC_010545	2290229	2290083	48	54.17	0	FALSE	FALSE	Corynebacterineae	Corynebacteriaceae	Corynebacterium	FALSE
NC_003197	3096260	3096114	48	50	0	FALSE	FALSE	Enterobacteriales	Enterobacteriaceae	Salmonella	FALSE
NC_002939	1523599	1523766	55	53.22	0	FALSE	FALSE	Desulfuromonadales	Geobacteraceae	Geobacter	TRUE
NC_009439	4138745	4138599	48	56.25	0	FALSE	FALSE	Pseudomonadales	Pseudomonadaceae	Pseudomonas	FALSE
NC_002939	1524883	1525047	54	52	0	FALSE	FALSE	Desulfuromonadales	Geobacteraceae	Geobacter	FALSE
NC_007164	776078	775932	48	52.08	0	FALSE	FALSE	Corynebacterineae	Corynebacteriaceae	Corynebacterium	FALSE
NC_007164	776139	775993	48	56.25	0	FALSE	FALSE	Corynebacterineae	Corynebacteriaceae	Corynebacterium	FALSE
NC_009439	4139603	4139457	48	56.62	0	FALSE	FALSE	Pseudomonadales	Pseudomonadaceae	Pseudomonas	FALSE
NC_009439	4139847	4139701	48	56.25	0	FALSE	FALSE	Pseudomonadales	Pseudomonadaceae	Pseudomonas	FALSE
NC_009943	3590273	3590437	54	53.13	0	FALSE	FALSE	Desulfobacterales	Desulfobacteraceae	Desulfococcus	FALSE
NC_007164	775364	775200	54	51.16	0	FALSE	FALSE	Corynebacterineae	Corynebacteriaceae	Corynebacterium	FALSE
NC_003197	3078092	3077940	50	57.17	0	FALSE	FALSE	Enterobacteriales	Enterobacteriaceae	Salmonella	FALSE
NC_007164	775590	775444	48	56.25	0	FALSE	FALSE	Corynebacterineae	Corynebacteriaceae	Corynebacterium	FALSE
NC_010545	2290839	2290693	48	56.25	0	FALSE	FALSE	Corynebacterineae	Corynebacteriaceae	Corynebacterium	FALSE
NC_009943	3590657	3590803	48	44.87	0	FALSE	FALSE	Desulfobacterales	Desulfobacteraceae	Desulfococcus	FALSE
NC_007164	773699	773553	48	50	0	FALSE	FALSE	Corynebacterineae	Corynebacteriaceae	Corynebacterium	FALSE
NC_007164	774126	773980	48	54.17	0	FALSE	FALSE	Corynebacterineae	Corynebacteriaceae	Corynebacterium	FALSE
NC_010545	2289863	2289717	48	56.25	0	FALSE	FALSE	Corynebacterineae	Corynebacteriaceae	Corynebacterium	FALSE
NC_010468	1045281	1045448	55	48.59	0	FALSE	FALSE	Enterobacteriales	Enterobacteriaceae	Escherichia	FALSE
NC_010545	2291449	2291303	48	54.17	0	FALSE	FALSE	Corynebacterineae	Corynebacteriaceae	Corynebacterium	FALSE
NC_010545	2289497	2289351	48	52.23	0	FALSE	FALSE	Corynebacterineae	Corynebacteriaceae	Corynebacterium	FALSE
NC_009439	4138379	4138233	48	58.33	0	FALSE	FALSE	Pseudomonadales	Pseudomonadaceae	Pseudomonas	FALSE
NC_010545	2289954	2289778	58	51.4	0	FALSE	FALSE	Corynebacterineae	Corynebacteriaceae	Corynebacterium	FALSE
NC_009468	35421	35567	48	56.25	0	FALSE	FALSE	Rhodospirillales	Acetobacteraceae	Acidiphilium	FALSE
NC_009468	36336	36482	48	60.42	0	FALSE	FALSE	Rhodospirillales	Acetobacteraceae	Acidiphilium	FALSE
NC_009943	3591755	3591901	48	46.05	0	FALSE	FALSE	Desulfobacterales	Desulfobacteraceae	Desulfococcus	FALSE
NC_009439	4138583	4138416	55	51.02	0	FALSE	FALSE	Pseudomonadales	Pseudomonadaceae	Pseudomonas	FALSE
NC_003197	3095650	3095504	48	60.42	0	FALSE	FALSE	Enterobacteriales	Enterobacteriaceae	Salmonella	FALSE
NC_007164	773662	773492	56	50.3	0	FALSE	FALSE	Corynebacterineae	Corynebacteriaceae	Corynebacterium	TRUE
NC_007498	1137085	1137258	57	51.81	0	FALSE	FALSE	Desulfuromonadales	Pelobacteraceae	Pelobacter	FALSE
NC_009778	2778561	2778415	48	46.82	0	FALSE	FALSE	Enterobacteriales	Enterobacteriaceae	Cronobacter	FALSE
NC_009439	4138440	4138294	48	62.5	0	FALSE	FALSE	Pseudomonadales	Pseudomonadaceae	Pseudomonas	FALSE
NC_009943	3591694	3591840	48	49.5	0	FALSE	FALSE	Desulfobacterales	Desulfobacteraceae	Desulfococcus	FALSE
NC_008554	3492721	3492891	56	48.36	0	FALSE	FALSE	Syntrophobacterales	Syntrophobacteraceae	Syntrophobacter	FALSE
NC_010545	2291144	2290998	48	56.25	0	FALSE	FALSE	Corynebacterineae	Corynebacteriaceae	Corynebacterium	FALSE
NC_007164	773601	773431	56	54.17	0	FALSE	FALSE	Corynebacterineae	Corynebacteriaceae	Corynebacterium	FALSE
NC_010545	2289823	2289656	55	52.67	0	FALSE	FALSE	Corynebacterineae	Corynebacteriaceae	Corynebacterium	FALSE
NC_009468	36086	36238	50	49.88	0	FALSE	FALSE	Rhodospirillales	Acetobacteraceae	Acidiphilium	FALSE
NC_009439	4149552	4149406	48	52.9	0	FALSE	FALSE	Pseudomonadales	Pseudomonadaceae	Pseudomonas	FALSE
NC_007164	773854	773675	59	51.01	0	FALSE	FALSE	Corynebacterineae	Corynebacteriaceae	Corynebacterium	FALSE
NC_007164	775407	775261	48	58.33	0	FALSE	FALSE	Corynebacterineae	Corynebacteriaceae	Corynebacterium	FALSE
NC_007498	1140765	1140917	50	48.41	0	FALSE	FALSE	Desulfuromonadales	Pelobacteraceae	Pelobacter	FALSE
NC_007164	775498	775322	58	55.97	0	FALSE	FALSE	Corynebacterineae	Corynebacteriaceae	Corynebacterium	FALSE
NC_010545	2289765	2289595	56	52.23	0	FALSE	FALSE	Corynebacterineae	Corynebacteriaceae	Corynebacterium	FALSE
NC_010545	2291049	2290876	57	43.12	0	FALSE	FALSE	Corynebacterineae	Corynebacteriaceae	Corynebacterium	FALSE
NC_009439	4150040	4149894	48	60.42	0	FALSE	FALSE	Pseudomonadales	Pseudomonadaceae	Pseudomonas	FALSE
NC_008782	2077265	2077465	66	52.71	369	FALSE	FALSE	Burkholderiales	Comamonadaceae	Acidovorax	TRUE
NC_008825	2573808	2574008	66	53.91	369	FALSE	FALSE	Burkholderiales	Methylibium	Methylibium	TRUE

NC_008346	1444925	1444671	84	52.38	235	TRUE	FALSE	Synergistetes	Syntrophomonadaceae	Syntrophomonas	FALSE
NC_008346	1176868	1177122	84	52.38	235	TRUE	FALSE	Synergistetes	Syntrophomonadaceae	Syntrophomonas	FALSE
NC_008346	2256099	2255845	84	52.38	235	TRUE	FALSE	Synergistetes	Syntrophomonadaceae	Syntrophomonas	FALSE
NC_007907	5170972	5170718	84	52.38	235	TRUE	FALSE	Clostridiales	Peptococcaceae	Desulfitobacterium	FALSE
NC_005877	1454188	1454352	54	51.85	96	FALSE	FALSE	Thermoplasmatales	Picrophilaceae	Picrophilus	TRUE
NC_002689	1552513	1552677	54	51.85	96	FALSE	FALSE	Thermoplasmatales	Thermoplasmataceae	Thermoplasma	FALSE
NC_008343	2038875	2038663	70	51.43	349	FALSE	FALSE	Rhodospirillales	Acetobacteraceae	Granulibacter	FALSE
NC_009719	2650288	2650076	70	51.43	349	FALSE	FALSE	Rhizobiales	Phyllobacteriaceae	Parvibaculum	FALSE
NC_002939	1530393	1530539	48	56.25	35	FALSE	FALSE	Desulfuromonadales	Geobacteraceae	Geobacter	FALSE
NC_009943	3591450	3591596	48	54.17	35	FALSE	FALSE	Desulfobacterales	Desulfobacteraceae	Desulfococcus	FALSE
NC_008554	3492834	3493013	59	45.76	35	FALSE	FALSE	Syntrophobacterales	Syntrophobacteraceae	Syntrophobacter	FALSE
NC_008554	3490182	3490328	48	54.17	35	FALSE	FALSE	Syntrophobacterales	Syntrophobacteraceae	Syntrophobacter	FALSE
NC_009943	3591112	3591291	59	51.01	35	FALSE	FALSE	Desulfobacterales	Desulfobacteraceae	Desulfococcus	FALSE
NC_008554	3489542	3489718	58	45.69	35	FALSE	FALSE	Syntrophobacterales	Syntrophobacteraceae	Syntrophobacter	FALSE
NC_008639	1620648	1620851	67	51.13	374	TRUE	FALSE	Chlorobiales	Chlorobiaceae	Pelodictyon	TRUE
NC_010162	7592284	7592484	66	51.13	374	TRUE	FALSE	Sorangiineae	Polyangiaceae	Sorangium	TRUE
NC_010002	4544548	4544667	39	54.74	83	FALSE	FALSE	Burkholderiales	Comamonadaceae	Delftia	FALSE
NC_009138	1993425	1993568	47	42.43	83	FALSE	FALSE	Burkholderiales	Oxalobacteraceae	Herminiimonas	FALSE
NC_010170	4563768	4563649	39	54.74	83	FALSE	FALSE	Burkholderiales	Alcaligenaceae	Bordetella	FALSE
NC_010170	1096081	1096242	53	50.37	206	FALSE	FALSE	Burkholderiales	Alcaligenaceae	Bordetella	FALSE
NC_009719	3615591	3615782	63	50.37	206	FALSE	FALSE	Rhizobiales	Phyllobacteriaceae	Parvibaculum	FALSE
NC_007907	2558813	2558998	61	50.45	236	TRUE	FALSE	Clostridiales	Peptococcaceae	Desulfitobacterium	TRUE
NC_009922	1742514	1742338	58	49.75	236	TRUE	FALSE	Clostridiales	Clostridiaceae	Alkaliphilus	TRUE
NC_010002	4547622	4547497	41	49.71	162	FALSE	FALSE	Burkholderiales	Comamonadaceae	Delftia	TRUE
NC_010170	4560695	4560820	41	49.71	162	FALSE	FALSE	Burkholderiales	Alcaligenaceae	Bordetella	TRUE
NC_008563	4391777	4391989	70	49.65	23	TRUE	FALSE	Enterobacteriales	Enterobacteriaceae	Escherichia	TRUE
NC_007946	4359763	4359975	70	49.65	23	TRUE	FALSE	Enterobacteriales	Enterobacteriaceae	Escherichia	TRUE
NC_004347	388903	389118	71	49.65	23	TRUE	FALSE	Alteromonadales	Shewanellaceae	Shewanella	TRUE
NC_004431	4602429	4602641	70	49.65	23	TRUE	FALSE	Enterobacteriales	Enterobacteriaceae	Escherichia	TRUE
NC_009446	179489	179298	63	49.21	187	TRUE	FALSE	Cardiobacteriales	Cardiobacteriaceae	Dichelobacter	FALSE
NC_009997	3982520	3982711	63	49.21	187	TRUE	FALSE	Alteromonadales	Shewanellaceae	Shewanella	FALSE
NC_009712	2498614	2498817	67	54.14	144	FALSE	TRUE	Methanomicrobia	Methanomicrobiales	Candidatus Methanoregula	FALSE
NC_009051	226422	226222	66	43.61	144	FALSE	TRUE	Methanomicrobiales	Methanomicrobiaceae	Methanoculleus	TRUE
NC_009439	3776307	3776182	41	48.83	357	FALSE	FALSE	Pseudomonadales	Pseudomonadaceae	Pseudomonas	TRUE
NC_008390	2160690	2160538	50	48.83	357	FALSE	FALSE	Burkholderiales	Burkholderiaceae	Burkholderia	FALSE
NC_007973	3284591	3284409	60	48.78	292	FALSE	FALSE	Burkholderiales	Burkholderiaceae	Cupriavidus	FALSE
NC_010002	2999614	2999432	60	48.78	292	FALSE	FALSE	Burkholderiales	Comamonadaceae	Delftia	FALSE
NC_009012	203045	203269	74	48.72	143	TRUE	FALSE	Clostridiales	Clostridiaceae	Clostridium	FALSE
NC_009437	1730637	1730873	78	48.72	143	TRUE	FALSE	Thermoanaerobacterales	Thermoanaerobacterales	Caldicellulosiruptor	TRUE
NC_007951	3605838	3605695	47	48.67	249	FALSE	FALSE	Burkholderiales	Burkholderiaceae	Burkholderia	FALSE
NC_010170	1551594	1551451	47	48.67	249	FALSE	FALSE	Burkholderiales	Alcaligenaceae	Bordetella	FALSE
NC_010002	3007435	3007590	51	48.52	296	FALSE	FALSE	Burkholderiales	Comamonadaceae	Delftia	FALSE
NC_007973	3292412	3292567	51	48.52	296	FALSE	FALSE	Burkholderiales	Burkholderiaceae	Cupriavidus	FALSE
NC_009615	2623649	2623473	58	48.06	132	TRUE	FALSE	Bacteroidales	Porphyromonadaceae	Parabacteroides	FALSE
NC_004663	1417593	1417417	58	48.06	132	TRUE	FALSE	Bacteroidales	Bacteroidaceae	Bacteroides	FALSE
NC_007951	3570479	3570769	96	47.98	244	TRUE	FALSE	Burkholderiales	Burkholderiaceae	Burkholderia	FALSE
NC_010170	1504599	1504889	96	47.98	244	TRUE	FALSE	Burkholderiales	Alcaligenaceae	Bordetella	FALSE
NC_000911	1346038	1345775	87	45.01	90	TRUE	TRUE	Cyanobacteria	Chroococcales	Synechocystis	TRUE
NC_007413	5682050	5681802	82	50.93	90	TRUE	TRUE	Nostocales	Nostocaceae	Anabaena	FALSE
NC_010086	618896	619306	136	45.99	69	TRUE	FALSE	Burkholderiales	Burkholderiaceae	Burkholderia	FALSE
NC_007298	3139684	3140100	138	48.18	69	TRUE	FALSE	Rhodocyclales	Rhodocyclaceae	Dechloromonas	TRUE
NC_009456	870532	870122	136	48.18	69	TRUE	FALSE	Vibrionales	Vibrionaceae	Vibrio	FALSE
NC_010170	1106844	1106948	34	48.16	16	TRUE	FALSE	Burkholderiales	Alcaligenaceae	Bordetella	FALSE
NC_009138	1993468	1993584	38	46.44	16	TRUE	FALSE	Burkholderiales	Oxalobacteraceae	Herminiimonas	FALSE

NC_009719	3626292	3626393	33	46.78	16	TRUE	FALSE	Rhizobiales	Phyllobacteriaceae	Parvibaculum	FALSE
NC_007973	2520582	2520683	33	46.78	16	TRUE	FALSE	Burkholderiales	Burkholderiaceae	Cupriavidus	FALSE
NC_008782	1505167	1505063	34	48.16	16	TRUE	FALSE	Burkholderiales	Comamonadaceae	Acidovorax	FALSE
NC_010943	1352656	1352408	82	46.9	92	FALSE	FALSE	Xanthomonadales	Xanthomonadaceae	Stenotrophomonas	FALSE
NC_008752	745058	745288	76	46.9	92	FALSE	FALSE	Burkholderiales	Comamonadaceae	Acidovorax	FALSE
NC_010999	263078	262971	35	38.94	161	TRUE	FALSE	Lactobacillales	Lactobacillaceae	Lactobacillus	FALSE
NC_010999	2640196	2640303	35	38.94	161	TRUE	FALSE	Lactobacillales	Lactobacillaceae	Lactobacillus	FALSE
NC_010999	1987052	1987159	35	38.94	161	TRUE	FALSE	Lactobacillales	Lactobacillaceae	Lactobacillus	FALSE
NC_005957	291218	291111	35	85.71	161	TRUE	FALSE	Bacillales	Bacillaceae	Bacillus	FALSE
NC_010999	912275	912168	35	38.94	161	TRUE	FALSE	Lactobacillales	Lactobacillaceae	Lactobacillus	FALSE
NC_010999	890597	890490	35	38.94	161	TRUE	FALSE	Lactobacillales	Lactobacillaceae	Lactobacillus	FALSE
NC_009778	2778155	2777916	79	49.71	36	FALSE	FALSE	Enterobacteriales	Enterobacteriaceae	Cronobacter	FALSE
NC_009439	4139236	4139027	69	41.48	36	FALSE	FALSE	Pseudomonadales	Pseudomonadaceae	Pseudomonas	FALSE
NC_002939	1527219	1527452	77	48.13	36	FALSE	FALSE	Desulfuromonadales	Geobacteraceae	Geobacter	FALSE
NC_008782	1515741	1516085	114	46.27	209	FALSE	FALSE	Burkholderiales	Comamonadaceae	Acidovorax	FALSE
NC_007508	2740271	2740615	114	46.27	209	FALSE	FALSE	Xanthomonadales	Xanthomonadaceae	Xanthomonas	FALSE
NC_010628	7062754	7062599	51	42.02	195	TRUE	FALSE	Nostocales	Nostocaceae	Nostoc	TRUE
NC_007413	532156	532004	50	49.51	195	TRUE	FALSE	Nostocales	Nostocaceae	Anabaena	TRUE
NC_010688	2804632	2804510	40	31.21	12	FALSE	FALSE	Xanthomonadales	Xanthomonadaceae	Xanthomonas	FALSE
NC_010170	4568155	4568280	41	48.61	12	FALSE	FALSE	Burkholderiales	Alcaligenaceae	Bordetella	TRUE
NC_007508	2625395	2625517	40	69.15	12	FALSE	FALSE	Xanthomonadales	Xanthomonadaceae	Xanthomonas	FALSE
NC_007086	2481147	2481269	40	31.21	12	FALSE	FALSE	Xanthomonadales	Xanthomonadaceae	Xanthomonas	FALSE
NC_010002	4540161	4540036	41	48.61	12	FALSE	FALSE	Burkholderiales	Comamonadaceae	Delftia	FALSE
NC_009076	3806087	3806314	75	48.03	371	TRUE	FALSE	Burkholderiales	Burkholderiaceae	Burkholderia	FALSE
NC_010162	9212772	9212512	86	42.43	371	TRUE	FALSE	Sorangiineae	Polyangiaceae	Sorangium	TRUE
NC_010170	1502468	1502286	60	45	245	FALSE	FALSE	Burkholderiales	Alcaligenaceae	Bordetella	FALSE
NC_007951	3568348	3568166	60	45	245	FALSE	FALSE	Burkholderiales	Burkholderiaceae	Burkholderia	FALSE
NC_009256	2737436	2737624	62	44.72	91	TRUE	TRUE	Burkholderiales	Burkholderiaceae	Burkholderia	TRUE
NC_008752	1921941	1922144	67	45.03	91	TRUE	TRUE	Burkholderiales	Comamonadaceae	Acidovorax	TRUE
NC_010170	1502358	1502239	39	44.12	238	FALSE	FALSE	Burkholderiales	Alcaligenaceae	Bordetella	FALSE
NC_007951	3568238	3568119	39	44.12	238	FALSE	FALSE	Burkholderiales	Burkholderiaceae	Burkholderia	FALSE
NC_007973	2520526	2520675	49	44.73	14	FALSE	FALSE	Burkholderiales	Burkholderiaceae	Cupriavidus	FALSE
NC_010170	1504717	1504866	49	43.31	14	FALSE	FALSE	Burkholderiales	Alcaligenaceae	Bordetella	FALSE
NC_009719	3626236	3626385	49	44.73	14	FALSE	FALSE	Rhizobiales	Phyllobacteriaceae	Parvibaculum	FALSE
NC_009138	1993412	1993561	49	42.14	14	FALSE	FALSE	Burkholderiales	Oxalobacteraceae	Herminiimonas	FALSE
NC_007951	3570597	3570746	49	43.31	14	FALSE	FALSE	Burkholderiales	Burkholderiaceae	Burkholderia	FALSE
NC_007907	46804	47046	80	43.48	233	TRUE	FALSE	Clostridiales	Peptococcaceae	Desulfitobacterium	TRUE
NC_009706	3926247	3926492	81	43.48	233	TRUE	FALSE	Clostridiales	Clostridiaceae	Clostridium	FALSE
NC_010170	1511639	1511514	41	43.46	252	FALSE	FALSE	Burkholderiales	Alcaligenaceae	Bordetella	TRUE
NC_007951	3572740	3572615	41	43.46	252	FALSE	FALSE	Burkholderiales	Burkholderiaceae	Burkholderia	TRUE
NC_010120	1595822	1595971	49	47.15	29	TRUE	FALSE	Neisseriales	Neisseriaceae	Neisseria	FALSE
NC_010120	56837	56688	49	47.15	29	TRUE	FALSE	Neisseriales	Neisseriaceae	Neisseria	FALSE
NC_010943	4786646	4786774	42	39.45	29	TRUE	FALSE	Xanthomonadales	Xanthomonadaceae	Stenotrophomonas	FALSE
NC_010528	2926250	2926399	49	42.68	29	TRUE	FALSE	Burkholderiales	Burkholderiaceae	Cupriavidus	FALSE
NC_010943	416748	416620	42	39.45	29	TRUE	FALSE	Xanthomonadales	Xanthomonadaceae	Stenotrophomonas	FALSE
NC_010530	658938	658789	49	42.68	29	TRUE	FALSE	Burkholderiales	Burkholderiaceae	Cupriavidus	FALSE
NC_010528	1623149	1623000	49	42.68	29	TRUE	FALSE	Burkholderiales	Burkholderiaceae	Cupriavidus	FALSE
NC_010120	1890595	1890744	49	47.15	29	TRUE	FALSE	Neisseriales	Neisseriaceae	Neisseria	FALSE
NC_010943	422412	422284	42	39.45	29	TRUE	FALSE	Xanthomonadales	Xanthomonadaceae	Stenotrophomonas	FALSE
NC_010120	182923	182774	49	47.15	29	TRUE	FALSE	Neisseriales	Neisseriaceae	Neisseria	FALSE
NC_010943	4792310	4792438	42	39.45	29	TRUE	FALSE	Xanthomonadales	Xanthomonadaceae	Stenotrophomonas	FALSE
NC_008752	739621	739908	95	42.13	30	FALSE	FALSE	Burkholderiales	Comamonadaceae	Acidovorax	FALSE
NC_010084	2531865	2532155	96	46.65	30	FALSE	FALSE	Burkholderiales	Burkholderiaceae	Burkholderia	FALSE
NC_003295	2820152	2819865	95	40.39	30	FALSE	FALSE	Burkholderiales	Burkholderiaceae	Ralstonia	FALSE

NC_003295	2814637	2814906	89	38.23	27	FALSE	FALSE	Burkholderiales	Burkholderiaceae	Ralstonia	FALSE
NC_010002	3000613	3000945	110	43.11	27	FALSE	FALSE	Burkholderiales	Comamonadaceae	Delftia	TRUE
NC_007973	3285590	3285922	110	43.11	27	FALSE	FALSE	Burkholderiales	Burkholderiaceae	Cupriavidus	TRUE
NC_010170	2263881	2263609	90	44.98	27	FALSE	FALSE	Burkholderiales	Alcaligenaceae	Bordetella	FALSE
NC_007973	1601725	1601453	90	43.65	27	FALSE	FALSE	Burkholderiales	Burkholderiaceae	Cupriavidus	FALSE
NC_010556	174099	173782	105	42.47	94	TRUE	FALSE	Bacillales	Bacillales Family XII. Incertae Sedis	Exiguobacterium	FALSE
NC_002570	355699	355295	134	42.47	94	TRUE	FALSE	Bacillales	Bacillaceae	Bacillus	FALSE
NC_008782	2819584	2819417	55	24.36	310	FALSE	FALSE	Burkholderiales	Comamonadaceae	Acidovorax	FALSE
NC_007973	1373570	1373385	61	60.51	310	FALSE	FALSE	Burkholderiales	Burkholderiaceae	Cupriavidus	FALSE
NC_010080	1588640	1588969	109	41.67	352	FALSE	FALSE	Lactobacillales	Lactobacillaceae	Lactobacillus	TRUE
NC_010556	173709	173386	107	41.67	352	FALSE	FALSE	Bacillales	Bacillales Family XII. Incertae Sedis	Exiguobacterium	TRUE
NC_010002	4544647	4544516	43	41.48	120	FALSE	FALSE	Burkholderiales	Comamonadaceae	Delftia	FALSE
NC_010170	4563669	4563800	43	41.48	120	FALSE	FALSE	Burkholderiales	Alcaligenaceae	Bordetella	FALSE
NC_009253	405681	405460	73	45.11	355	TRUE	TRUE	Clostridiales	Peptococcaceae	Desulfotomaculum	TRUE
NC_009253	2335442	2335221	73	43.7	355	TRUE	TRUE	Clostridiales	Peptococcaceae	Desulfotomaculum	FALSE
NC_008346	1518206	1517997	69	35.24	355	TRUE	TRUE	Synergistetes	Syntrophomonadaceae	Syntrophomonas	TRUE
NC_010170	2263930	2263739	63	41.62	28	FALSE	FALSE	Burkholderiales	Alcaligenaceae	Bordetella	FALSE
NC_007973	1601774	1601583	63	42.65	28	FALSE	FALSE	Burkholderiales	Burkholderiaceae	Cupriavidus	FALSE
NC_008782	3110520	3110711	63	41.07	28	FALSE	FALSE	Burkholderiales	Comamonadaceae	Acidovorax	FALSE
NC_010943	1354309	1354500	63	38.45	28	FALSE	FALSE	Xanthomonadales	Xanthomonadaceae	Stenotrophomonas	FALSE
NC_008711	2649260	2649069	63	25.29	375	TRUE	FALSE	Micrococcineae	Micrococcaceae	Arthrobacter	FALSE
NC_009142	1744462	1744680	72	56.55	375	TRUE	FALSE	Pseudonocardineae	Pseudonocardiaceae	Saccharopolyspora	TRUE
NC_008312	7701054	7700941	37	40.41	194	TRUE	FALSE	Cyanobacteria	Oscillatoriales	Trichodesmium	FALSE
NC_008312	1129490	1129377	37	40.41	194	TRUE	FALSE	Cyanobacteria	Oscillatoriales	Trichodesmium	FALSE
NC_007410	262286	262399	37	42.03	194	TRUE	FALSE	Nostocales	Nostocaceae	Anabaena	FALSE
NC_008312	1234912	1235025	37	40.41	194	TRUE	FALSE	Cyanobacteria	Oscillatoriales	Trichodesmium	FALSE
NC_010943	4791512	4791667	51	46.85	6	TRUE	FALSE	Xanthomonadales	Xanthomonadaceae	Stenotrophomonas	FALSE
NC_010102	2814335	2814490	51	45.77	6	TRUE	FALSE	Enterobacteriales	Enterobacteriaceae	Salmonella	FALSE
NC_010067	4153090	4152935	51	45.77	6	TRUE	FALSE	Enterobacteriales	Enterobacteriaceae	Salmonella	FALSE
NC_009053	2066789	2066634	51	43.12	6	TRUE	FALSE	Pasteurellales	Pasteurellaceae	Actinobacillus	FALSE
NC_010999	259989	259834	51	29.81	6	TRUE	FALSE	Lactobacillales	Lactobacillaceae	Lactobacillus	FALSE
NC_010999	1990349	1990504	51	29.81	6	TRUE	FALSE	Lactobacillales	Lactobacillaceae	Lactobacillus	FALSE
NC_010943	423210	423055	51	46.85	6	TRUE	FALSE	Xanthomonadales	Xanthomonadaceae	Stenotrophomonas	FALSE
NC_010939	1786250	1786405	51	43.12	6	TRUE	FALSE	Pasteurellales	Pasteurellaceae	Actinobacillus	FALSE
NC_010999	2643493	2643648	51	29.81	6	TRUE	FALSE	Lactobacillales	Lactobacillaceae	Lactobacillus	FALSE
NC_009053	279839	279684	51	43.12	6	TRUE	FALSE	Pasteurellales	Pasteurellaceae	Actinobacillus	FALSE
NC_010939	1898465	1898620	51	43.12	6	TRUE	FALSE	Pasteurellales	Pasteurellaceae	Actinobacillus	FALSE
NC_010067	3600418	3600573	51	40.96	6	TRUE	FALSE	Enterobacteriales	Enterobacteriaceae	Salmonella	FALSE
NC_010102	4142596	4142441	51	45.77	6	TRUE	FALSE	Enterobacteriales	Enterobacteriaceae	Salmonella	FALSE
NC_010999	909186	909031	51	29.81	6	TRUE	FALSE	Lactobacillales	Lactobacillaceae	Lactobacillus	FALSE
NC_010943	4785848	4786003	51	46.85	6	TRUE	FALSE	Xanthomonadales	Xanthomonadaceae	Stenotrophomonas	FALSE
NC_010939	1592111	1592266	51	43.12	6	TRUE	FALSE	Pasteurellales	Pasteurellaceae	Actinobacillus	FALSE
NC_010943	417546	417391	51	46.85	6	TRUE	FALSE	Xanthomonadales	Xanthomonadaceae	Stenotrophomonas	FALSE
NC_010999	887508	887353	51	29.81	6	TRUE	FALSE	Lactobacillales	Lactobacillaceae	Lactobacillus	FALSE
NC_009053	1761960	1762115	51	43.12	6	TRUE	FALSE	Pasteurellales	Pasteurellaceae	Actinobacillus	FALSE
NC_010510	545666	545472	64	51.94	13	TRUE	TRUE	Rhizobiales	Methylobacteriaceae	Methylobacterium	FALSE
NC_010505	4686528	4686343	61	28.53	13	TRUE	TRUE	Rhizobiales	Methylobacteriaceae	Methylobacterium	TRUE
NC_009668	1601245	1601415	56	43.18	13	TRUE	TRUE	Rhizobiales	Brucellaceae	Ochrobactrum	FALSE
NC_010505	3700168	3700350	60	28.57	13	TRUE	TRUE	Rhizobiales	Methylobacteriaceae	Methylobacterium	FALSE
NC_010161	1853938	1854141	67	47.44	13	TRUE	TRUE	Rhizobiales	Bartonellaceae	Bartonella	TRUE
NC_010161	2060335	2060538	67	47.44	13	TRUE	TRUE	Rhizobiales	Bartonellaceae	Bartonella	TRUE
NC_009667	1086342	1086172	56	42.95	13	TRUE	TRUE	Rhizobiales	Brucellaceae	Ochrobactrum	FALSE
NC_009668	459147	458977	56	43.18	13	TRUE	TRUE	Rhizobiales	Brucellaceae	Ochrobactrum	FALSE
NC_009667	1348565	1348395	56	42.95	13	TRUE	TRUE	Rhizobiales	Brucellaceae	Ochrobactrum	FALSE

NC_010505	1591610	1591428	60	28.57	13	TRUE	TRUE	Rhizobiales	Methylobacteriaceae	Methylobacterium	FALSE
NC_007973	3290327	3290545	72	40.29	289	TRUE	FALSE	Burkholderiales	Burkholderiaceae	Cupriavidus	FALSE
NC_010002	3005350	3005568	72	40.29	289	TRUE	FALSE	Burkholderiales	Comamonadaceae	Delftia	FALSE
NC_008786	5009081	5008896	61	59.02	103	FALSE	TRUE	Burkholderiales	Comamonadaceae	Verminephrobacter	FALSE
NC_003295	866813	866622	63	16.53	103	FALSE	TRUE	Burkholderiales	Burkholderiaceae	Ralstonia	FALSE
NC_003295	942844	942659	61	44	103	FALSE	TRUE	Burkholderiales	Burkholderiaceae	Ralstonia	FALSE
NC_003106	1296361	1296215	48	35.06	42	TRUE	FALSE	Sulfolobales	Sulfolobaceae	Sulfolobus	FALSE
NC_008698	478072	478236	54	42.82	42	TRUE	FALSE	Thermoproteales	Thermofilaceae	Thermofilum	FALSE
NC_009440	1692969	1692799	56	41.21	42	TRUE	FALSE	Sulfolobales	Sulfolobaceae	Metallosphaera	FALSE
NC_008577	2160177	2160410	77	39.39	348	FALSE	FALSE	Alteromonadales	Shewanellaceae	Shewanella	TRUE
NC_008321	2035250	2035483	77	39.39	348	FALSE	FALSE	Alteromonadales	Shewanellaceae	Shewanella	TRUE
NC_009719	3621855	3621589	88	40.88	189	FALSE	FALSE	Rhizobiales	Phyllobacteriaceae	Parvibaculum	TRUE
NC_009138	1989075	1988797	92	36.49	189	FALSE	FALSE	Burkholderiales	Oxalobacteraceae	Herminiimonas	TRUE
NC_009012	2220301	2220080	73	40.72	141	TRUE	FALSE	Clostridiales	Clostridiaceae	Clostridium	TRUE
NC_010382	2555084	2555281	65	33.45	141	TRUE	FALSE	Bacillales	Planococcaceae	Lysinibacillus	TRUE
NC_009012	1337583	1337353	76	41.39	141	TRUE	FALSE	Clostridiales	Clostridiaceae	Clostridium	TRUE
NC_009012	1011345	1011686	113	35.85	142	FALSE	FALSE	Clostridiales	Clostridiaceae	Clostridium	FALSE
NC_010424	1075027	1074740	95	41.09	142	FALSE	FALSE	Clostridiales	Peptococcaceae	Candidatus Desulforudis	FALSE
NC_006513	329733	329870	45	36.79	173	FALSE	FALSE	Rhodocyclales	Rhodocyclaceae	Azoarcus	FALSE
NC_009228	69384	69518	44	38.74	173	FALSE	FALSE	Burkholderiales	Burkholderiaceae	Burkholderia	FALSE
NC_009228	37982	38116	44	38.74	173	FALSE	FALSE	Burkholderiales	Burkholderiaceae	Burkholderia	FALSE
NC_007508	2485181	2485378	65	37.16	216	FALSE	TRUE	Xanthomonadales	Xanthomonadaceae	Xanthomonas	FALSE
NC_008463	1316835	1317059	74	38.93	216	FALSE	TRUE	Pseudomonadales	Pseudomonadaceae	Pseudomonas	FALSE
NC_009901	4848184	4847909	91	38.01	339	FALSE	TRUE	Alteromonadales	Shewanellaceae	Shewanella	TRUE
NC_008228	3615836	3615564	90	37.57	339	FALSE	TRUE	Alteromonadales	Pseudoalteromonadaceae	Pseudoalteromonas	FALSE
NC_008554	849236	849087	49	37.07	364	FALSE	FALSE	Syntrophobacterales	Syntrophobacteraceae	Syntrophobacter	FALSE
NC_008554	3186648	3186797	49	37.07	364	FALSE	FALSE	Syntrophobacterales	Syntrophobacteraceae	Syntrophobacter	FALSE
NC_009943	683262	683113	49	38.15	364	FALSE	FALSE	Desulfobacterales	Desulfobacteraceae	Desulfococcus	FALSE
NC_010120	1595633	1595794	53	37.38	149	FALSE	FALSE	Neisseriales	Neisseriaceae	Neisseria	FALSE
NC_010943	4786457	4786618	53	37.38	149	FALSE	FALSE	Xanthomonadales	Xanthomonadaceae	Stenotrophomonas	FALSE
NC_010943	422601	422440	53	37.38	149	FALSE	FALSE	Xanthomonadales	Xanthomonadaceae	Stenotrophomonas	FALSE
NC_010120	183112	182951	53	37.38	149	FALSE	FALSE	Neisseriales	Neisseriaceae	Neisseria	FALSE
NC_010943	416937	416776	53	37.38	149	FALSE	FALSE	Xanthomonadales	Xanthomonadaceae	Stenotrophomonas	FALSE
NC_010120	1890406	1890567	53	37.38	149	FALSE	FALSE	Neisseriales	Neisseriaceae	Neisseria	FALSE
NC_010120	57026	56865	53	37.38	149	FALSE	FALSE	Neisseriales	Neisseriaceae	Neisseria	FALSE
NC_010943	4792121	4792282	53	37.38	149	FALSE	FALSE	Xanthomonadales	Xanthomonadaceae	Stenotrophomonas	FALSE
NC_007644	393410	393583	57	37.34	230	TRUE	FALSE	Thermoanaerobacterales	Thermoanaerobacteraceae	Moorella	FALSE
NC_007907	2058645	2058442	67	37.34	230	TRUE	FALSE	Clostridiales	Peptococcaceae	Desulfitobacterium	TRUE
NC_009454	934175	933999	58	23.21	368	FALSE	TRUE	Clostridiales	Peptococcaceae	Pelotomaculum	FALSE
NC_008593	1690402	1690548	48	51.4	368	FALSE	TRUE	Clostridiales	Clostridiaceae	Clostridium	FALSE
NC_008752	730848	730708	46	38.31	379	FALSE	FALSE	Burkholderiales	Comamonadaceae	Acidovorax	FALSE
NC_003295	2825804	2825944	46	35.75	379	FALSE	FALSE	Burkholderiales	Burkholderiaceae	Ralstonia	FALSE
NC_003888	1045895	1046065	56	52.45	119	FALSE	FALSE	Streptomycineae	Streptomycetaceae	Streptomyces	FALSE
NC_008596	6690147	6689992	51	21.1	119	FALSE	FALSE	Corynebacterineae	Mycobacteriaceae	Mycobacterium	FALSE
NC_008766	26767	26639	42	36.75	2	FALSE	FALSE	Burkholderiales	Comamonadaceae	Acidovorax	TRUE
NC_008712	239675	239547	42	36.75	2	FALSE	FALSE	Micrococcineae	Micrococcaceae	Arthrobacter	FALSE
NC_007973	1665101	1664973	42	36.75	2	FALSE	FALSE	Burkholderiales	Burkholderiaceae	Cupriavidus	FALSE
NC_008712	230898	230770	42	36.75	2	FALSE	FALSE	Micrococcineae	Micrococcaceae	Arthrobacter	FALSE
NC_008385	27073	26945	42	36.75	2	FALSE	FALSE	Burkholderiales	Burkholderiaceae	Burkholderia	TRUE
NC_008782	3171483	3171355	42	36.75	2	FALSE	FALSE	Burkholderiales	Comamonadaceae	Acidovorax	FALSE
NC_007972	160747	160619	42	36.75	2	FALSE	FALSE	Burkholderiales	Burkholderiaceae	Cupriavidus	FALSE
NC_007953	1259909	1260037	42	36.75	2	FALSE	FALSE	Burkholderiales	Burkholderiaceae	Burkholderia	FALSE
NC_007973	1611193	1611065	42	36.75	2	FALSE	FALSE	Burkholderiales	Burkholderiaceae	Cupriavidus	FALSE
NC_008765	56795	56667	42	36.75	2	FALSE	FALSE	Burkholderiales	Comamonadaceae	Acidovorax	FALSE

NC_008782	2375617	2375745	42	36.75	2	FALSE	FALSE	Burkholderiales	Comamonadaceae	Acidovorax	FALSE
NC_008554	3922770	3922615	51	34.48	365	FALSE	FALSE	Syntrophobacterales	Syntrophobacteraceae	Syntrophobacter	TRUE
NC_008576	1073066	1072911	51	36.86	365	FALSE	FALSE	Bacteria	Proteobacteria	Magnetococcus	FALSE
NC_008576	1069475	1069320	51	38.05	365	FALSE	FALSE	Bacteria	Proteobacteria	Magnetococcus	FALSE
NC_008341	49482	49162	106	36.57	226	FALSE	FALSE	Nitrosomonadales	Nitrosomonadaceae	Nitrosomonas	TRUE
NC_007643	423685	424083	132	35.72	226	FALSE	FALSE	Rhodospirillales	Rhodospirillaceae	Rhodospirillum	TRUE
NC_009075	24922	24650	90	35.63	188	FALSE	FALSE	Burkholderiales	Burkholderiaceae	Burkholderia	FALSE
NC_008750	2541203	2540919	94	35.63	188	FALSE	FALSE	Alteromonadales	Shewanellaceae	Shewanella	TRUE
NC_009831	5413088	5412828	86	35.06	376	TRUE	TRUE	Alteromonadales	Shewanellaceae	Shewanella	TRUE
NC_008740	1131982	1132257	91	35.06	376	TRUE	TRUE	Alteromonadales	Alteromonadaceae	Marinobacter	TRUE
NC_008321	4102293	4102069	74	34.67	45	FALSE	TRUE	Alteromonadales	Shewanellaceae	Shewanella	TRUE
NC_008322	598478	598702	74	34.67	45	FALSE	TRUE	Alteromonadales	Shewanellaceae	Shewanella	TRUE
NC_004342	1795976	1796206	76	34.67	45	FALSE	TRUE	Spirochaetales	Leptospiraceae	Leptospira	FALSE
NC_010002	4547566	4547429	45	34.21	167	FALSE	TRUE	Burkholderiales	Comamonadaceae	Delftia	FALSE
NC_010170	4560751	4560888	45	34.21	167	FALSE	TRUE	Burkholderiales	Alcaligenaceae	Bordetella	FALSE
NC_005945	1510489	1510641	50	11.2	1	FALSE	FALSE	Bacillales	Bacillaceae	Bacillus	FALSE
NC_007530	1510538	1510690	50	11.2	1	FALSE	FALSE	Bacillales	Bacillaceae	Bacillus	FALSE
NC_009674	1422406	1422558	50	9.22	1	FALSE	FALSE	Bacillales	Bacillaceae	Bacillus	FALSE
NC_010520	234820	234975	51	63.37	1	FALSE	FALSE	Clostridiales	Clostridiaceae	Clostridium	FALSE
NC_009698	233743	233898	51	63.37	1	FALSE	FALSE	Clostridiales	Clostridiaceae	Clostridium	FALSE
NC_005957	1536683	1536835	50	11.2	1	FALSE	FALSE	Bacillales	Bacillaceae	Bacillus	FALSE
NC_008600	1569768	1569920	50	11.2	1	FALSE	FALSE	Bacillales	Bacillaceae	Bacillus	FALSE
NC_003997	1510415	1510567	50	11.2	1	FALSE	FALSE	Bacillales	Bacillaceae	Bacillus	FALSE
NC_010184	1549171	1549323	50	11.2	1	FALSE	FALSE	Bacillales	Bacillaceae	Bacillus	FALSE
NC_006274	1545205	1545357	50	11.2	1	FALSE	FALSE	Bacillales	Bacillaceae	Bacillus	FALSE
NC_009495	240923	241078	51	62.86	1	FALSE	FALSE	Clostridiales	Clostridiaceae	Clostridium	FALSE
NC_009617	3911129	3911257	42	65.71	1	FALSE	FALSE	Clostridiales	Clostridiaceae	Clostridium	FALSE
NC_003909	1660606	1660758	50	11.2	1	FALSE	FALSE	Bacillales	Bacillaceae	Bacillus	FALSE
NC_010516	244561	244716	51	63.37	1	FALSE	FALSE	Clostridiales	Clostridiaceae	Clostridium	FALSE
NC_009697	233742	233897	51	63.37	1	FALSE	FALSE	Clostridiales	Clostridiaceae	Clostridium	FALSE
NC_009699	236313	236468	51	63.37	1	FALSE	FALSE	Clostridiales	Clostridiaceae	Clostridium	FALSE
NC_010320	2358578	2358790	70	11.27	186	TRUE	TRUE	Thermoanaerobacterales	Thermoanaerobacteraceae	Thermoanaerobacter	FALSE
NC_010321	963561	963361	66	55.2	186	TRUE	TRUE	Thermoanaerobacterales	Thermoanaerobacteraceae	Thermoanaerobacter	FALSE
NC_008435	864063	863797	88	33.85	359	TRUE	FALSE	Rhizobiales	Bradyrhizobiaceae	Rhodopseudomonas	FALSE
NC_010581	3931134	3931376	80	32.32	359	TRUE	FALSE	Rhizobiales	Beijerinckiaceae	Beijerinckia	FALSE
NC_010581	683025	682783	80	32.32	359	TRUE	FALSE	Rhizobiales	Beijerinckiaceae	Beijerinckia	FALSE
NC_010170	2264397	2264561	54	33.51	332	TRUE	FALSE	Burkholderiales	Alcaligenaceae	Bordetella	FALSE
NC_007973	1602241	1602405	54	31.17	332	TRUE	FALSE	Burkholderiales	Burkholderiaceae	Cupriavidus	FALSE
NC_010688	2405313	2405471	52	29.22	9	FALSE	FALSE	Xanthomonadales	Xanthomonadaceae	Xanthomonas	FALSE
NC_003902	2496822	2496664	52	29.22	9	FALSE	FALSE	Xanthomonadales	Xanthomonadaceae	Xanthomonas	FALSE
NC_009719	3712086	3711922	54	35.06	9	FALSE	FALSE	Rhizobiales	Phyllobacteriaceae	Parvibaculum	FALSE
NC_007973	2618173	2618015	52	35.27	9	FALSE	FALSE	Burkholderiales	Burkholderiaceae	Cupriavidus	FALSE
NC_007508	2593543	2593707	54	30.25	9	FALSE	FALSE	Xanthomonadales	Xanthomonadaceae	Xanthomonas	FALSE
NC_002946	561316	560969	115	31.57	100	TRUE	TRUE	Neisseriales	Neisseriaceae	Neisseria	FALSE
NC_010519	1883576	1883253	107	31.57	100	TRUE	TRUE	Pasteurellales	Pasteurellaceae	Histophilus	TRUE
NC_010067	4153298	4153173	41	30.47	185	FALSE	FALSE	Enterobacteriales	Enterobacteriaceae	Salmonella	FALSE
NC_010102	4046042	4045917	41	31.59	185	FALSE	FALSE	Enterobacteriales	Enterobacteriaceae	Salmonella	FALSE
NC_010067	3414113	3414238	41	30.47	185	FALSE	FALSE	Enterobacteriales	Enterobacteriaceae	Salmonella	FALSE
NC_010102	4142804	4142679	41	31.59	185	FALSE	FALSE	Enterobacteriales	Enterobacteriaceae	Salmonella	FALSE
NC_010102	2814127	2814252	41	31.59	185	FALSE	FALSE	Enterobacteriales	Enterobacteriaceae	Salmonella	FALSE
NC_010102	4293838	4293713	41	31.59	185	FALSE	FALSE	Enterobacteriales	Enterobacteriaceae	Salmonella	FALSE
NC_010102	291396	291271	41	31.59	185	FALSE	FALSE	Enterobacteriales	Enterobacteriaceae	Salmonella	FALSE
NC_010102	3529323	3529448	41	31.59	185	FALSE	FALSE	Enterobacteriales	Enterobacteriaceae	Salmonella	FALSE
NC_010067	3693716	3693841	41	30.47	185	FALSE	FALSE	Enterobacteriales	Enterobacteriaceae	Salmonella	FALSE

NC_010067	2663045	2663170	41	30.47	185	FALSE	FALSE	Enterobacteriales	Enterobacteriaceae	Salmonella	FALSE
NC_010067	3600210	3600335	41	30.47	185	FALSE	FALSE	Enterobacteriales	Enterobacteriaceae	Salmonella	FALSE
NC_010067	261516	261391	41	30.47	185	FALSE	FALSE	Enterobacteriales	Enterobacteriaceae	Salmonella	FALSE
NC_010067	3460069	3460194	41	30.47	185	FALSE	FALSE	Enterobacteriales	Enterobacteriaceae	Salmonella	FALSE
NC_010102	4336852	4336727	41	31.59	185	FALSE	FALSE	Enterobacteriales	Enterobacteriaceae	Salmonella	FALSE
NC_009012	3788567	3788064	167	30.6	362	TRUE	FALSE	Clostridiales	Clostridiaceae	Clostridium	FALSE
NC_008553	676366	675884	160	31.21	362	TRUE	FALSE	Methanosarcinales	Methanosaetaceae	Methanosaeta	FALSE
NC_006177	727209	727003	68	18.73	168	FALSE	FALSE	Clostridiales	Clostridiales Family XVIII. Incertae Sedis	Symbiobacterium	FALSE
NC_009464	1749635	1749838	67	42.97	168	FALSE	FALSE	Archaea	Euryarchaeota	environmental	FALSE
NC_011027	1715682	1716092	136	28.74	193	FALSE	FALSE	Chlorobiales	Chlorobiaceae	Chlorobaculum	TRUE
NC_007406	898749	899201	150	28.74	193	FALSE	FALSE	Rhizobiales	Bradyrhizobiaceae	Nitrobacter	TRUE
NC_010999	262657	262523	44	28.41	165	TRUE	TRUE	Lactobacillales	Lactobacillaceae	Lactobacillus	FALSE
NC_010999	911854	911720	44	28.41	165	TRUE	TRUE	Lactobacillales	Lactobacillaceae	Lactobacillus	FALSE
NC_010999	2640617	2640751	44	28.41	165	TRUE	TRUE	Lactobacillales	Lactobacillaceae	Lactobacillus	FALSE
NC_010999	1987473	1987607	44	28.41	165	TRUE	TRUE	Lactobacillales	Lactobacillaceae	Lactobacillus	FALSE
NC_005957	290797	290663	44	30.3	165	TRUE	TRUE	Bacillales	Bacillaceae	Bacillus	FALSE
NC_010999	890176	890042	44	28.41	165	TRUE	TRUE	Lactobacillales	Lactobacillaceae	Lactobacillus	FALSE
NC_010102	4142266	4142078	62	27.92	184	TRUE	FALSE	Enterobacteriales	Enterobacteriaceae	Salmonella	FALSE
NC_010102	4045504	4045316	62	27.92	184	TRUE	FALSE	Enterobacteriales	Enterobacteriaceae	Salmonella	FALSE
NC_010067	2663583	2663771	62	27.92	184	TRUE	FALSE	Enterobacteriales	Enterobacteriaceae	Salmonella	FALSE
NC_010067	4152760	4152572	62	27.92	184	TRUE	FALSE	Enterobacteriales	Enterobacteriaceae	Salmonella	FALSE
NC_010102	2814665	2814853	62	27.92	184	TRUE	FALSE	Enterobacteriales	Enterobacteriaceae	Salmonella	FALSE
NC_010102	3529861	3530049	62	27.92	184	TRUE	FALSE	Enterobacteriales	Enterobacteriaceae	Salmonella	FALSE
NC_010102	4293300	4293112	62	27.92	184	TRUE	FALSE	Enterobacteriales	Enterobacteriaceae	Salmonella	FALSE
NC_010102	4336314	4336126	62	27.92	184	TRUE	FALSE	Enterobacteriales	Enterobacteriaceae	Salmonella	FALSE
NC_010067	3600748	3600936	62	27.92	184	TRUE	FALSE	Enterobacteriales	Enterobacteriaceae	Salmonella	FALSE
NC_010067	3414651	3414839	62	27.92	184	TRUE	FALSE	Enterobacteriales	Enterobacteriaceae	Salmonella	FALSE
NC_010102	290858	290670	62	27.92	184	TRUE	FALSE	Enterobacteriales	Enterobacteriaceae	Salmonella	FALSE
NC_010067	3460607	3460795	62	27.92	184	TRUE	FALSE	Enterobacteriales	Enterobacteriaceae	Salmonella	FALSE
NC_010067	260978	260790	62	27.92	184	TRUE	FALSE	Enterobacteriales	Enterobacteriaceae	Salmonella	FALSE
NC_010067	3694254	3694442	62	27.92	184	TRUE	FALSE	Enterobacteriales	Enterobacteriaceae	Salmonella	FALSE
NC_010002	4548737	4548522	71	26	334	TRUE	FALSE	Burkholderiales	Comamonadaceae	Delftia	FALSE
NC_010170	4559580	4559795	71	26	334	TRUE	FALSE	Burkholderiales	Alcaligenaceae	Bordetella	FALSE
NC_008463	2713931	2714185	84	25.37	314	TRUE	FALSE	Pseudomonadales	Pseudomonadaceae	Pseudomonas	FALSE
NC_007973	1367540	1367286	84	25.37	314	TRUE	FALSE	Burkholderiales	Burkholderiaceae	Cupriavidus	FALSE
NC_004459	2494322	2494855	177	25.35	48	TRUE	FALSE	Vibrionales	Vibrionaceae	Vibrio	TRUE
NC_008060	1326233	1326769	178	25.35	48	TRUE	FALSE	Burkholderiales	Burkholderiaceae	Burkholderia	TRUE
NC_008542	1869554	1870090	178	25.35	48	TRUE	FALSE	Burkholderiales	Burkholderiaceae	Burkholderia	TRUE
NC_010410	3648544	3648699	51	23.05	224	TRUE	FALSE	Pseudomonadales	Moraxellaceae	Acinetobacter	FALSE
NC_009656	5512659	5512504	51	23.05	224	TRUE	FALSE	Pseudomonadales	Pseudomonadaceae	Pseudomonas	FALSE
NC_009668	1601250	1601408	52	22.9	109	TRUE	FALSE	Rhizobiales	Brucellaceae	Ochrobactrum	FALSE
NC_009668	459142	458984	52	22.9	109	TRUE	FALSE	Rhizobiales	Brucellaceae	Ochrobactrum	FALSE
NC_009667	1348560	1348402	52	23.08	109	TRUE	FALSE	Rhizobiales	Brucellaceae	Ochrobactrum	FALSE
NC_009667	1086337	1086179	52	23.08	109	TRUE	FALSE	Rhizobiales	Brucellaceae	Ochrobactrum	FALSE
NC_007759	1704204	1704079	41	22.06	231	FALSE	TRUE	Syntrophobacterales	Syntrophaceae	Syntrophus	FALSE
NC_009446	208345	208220	41	23.48	231	FALSE	TRUE	Cardiobacteriales	Cardiobacteriaceae	Dichelobacter	FALSE
NC_004460	1626177	1626052	41	22.81	47	FALSE	TRUE	Vibrionales	Vibrionaceae	Vibrio	FALSE
NC_004459	2565912	2566040	42	18.35	47	FALSE	TRUE	Vibrionales	Vibrionaceae	Vibrio	FALSE
NC_005139	1890857	1890741	38	26.85	47	FALSE	TRUE	Vibrionales	Vibrionaceae	Vibrio	FALSE
NC_007907	5170700	5170948	82	18.39	234	TRUE	FALSE	Clostridiales	Peptococcaceae	Desulfitobacterium	FALSE
NC_008346	1177140	1176865	91	20.06	234	TRUE	FALSE	Synergistetes	Syntrophomonadaceae	Syntrophomonas	FALSE
NC_008346	1444653	1444928	91	20.06	234	TRUE	FALSE	Synergistetes	Syntrophomonadaceae	Syntrophomonas	FALSE
NC_008346	2255827	2256102	91	20.06	234	TRUE	FALSE	Synergistetes	Syntrophomonadaceae	Syntrophomonas	FALSE
NC_008741	32086	31922	54	19.68	354	FALSE	FALSE	Desulfovibrionales	Desulfovibrionaceae	Desulfovibrio	FALSE

NC_008346	566956	567123	55	18.84	354	FALSE	FALSE	Synergistetes	Syntrophomonadaceae	Syntrophomonas	FALSE
NC_008254	2953089	2952844	81	15.87	41	FALSE	FALSE	Rhizobiales	Phyllobacteriaceae	Mesorhizobium	FALSE
NC_009636	2892570	2892836	88	17	41	FALSE	FALSE	Rhizobiales	Rhizobiaceae	Sinorhizobium	TRUE
NC_003047	3105124	3105372	82	11.82	41	FALSE	FALSE	Rhizobiales	Rhizobiaceae	Sinorhizobium	TRUE
NC_007164	775605	775778	57	15.39	183	FALSE	TRUE	Corynebacterineae	Corynebacteriaceae	Corynebacterium	FALSE
NC_010545	2290546	2290722	58	16.62	183	FALSE	TRUE	Corynebacterineae	Corynebacteriaceae	Corynebacterium	FALSE
NC_007164	774702	774887	61	11.06	183	FALSE	TRUE	Corynebacterineae	Corynebacteriaceae	Corynebacterium	FALSE
NC_002939	813780	813409	123	18.37	98	FALSE	FALSE	Desulfuromonadales	Geobacteraceae	Geobacter	FALSE
NC_006677	2011606	2011247	119	9.63	98	FALSE	FALSE	Rhodospirillales	Acetobacteraceae	Gluconobacter	FALSE
NC_008346	170613	170239	124	7.95	228	FALSE	FALSE	Synergistetes	Syntrophomonadaceae	Syntrophomonas	FALSE
NC_008346	2795227	2795601	124	7.95	228	FALSE	FALSE	Synergistetes	Syntrophomonadaceae	Syntrophomonas	FALSE
NC_007644	2325419	2325042	125	40.96	228	FALSE	FALSE	Thermoanaerobacterales	Thermoanaerobacteraceae	Moorella	TRUE
NC_008346	221042	220668	124	7.95	228	FALSE	FALSE	Synergistetes	Syntrophomonadaceae	Syntrophomonas	FALSE
NC_008346	2171557	2171931	124	7.95	228	FALSE	FALSE	Synergistetes	Syntrophomonadaceae	Syntrophomonas	FALSE
NC_008346	362816	362442	124	7.95	228	FALSE	FALSE	Synergistetes	Syntrophomonadaceae	Syntrophomonas	FALSE
NC_008346	1944102	1944476	124	7.95	228	FALSE	FALSE	Synergistetes	Syntrophomonadaceae	Syntrophomonas	FALSE
NC_008346	177989	177615	124	7.14	228	FALSE	FALSE	Synergistetes	Syntrophomonadaceae	Syntrophomonas	FALSE
NC_008346	196991	196617	124	7.95	228	FALSE	FALSE	Synergistetes	Syntrophomonadaceae	Syntrophomonas	FALSE
NC_009085	3065055	3065363	102	14.98	367	TRUE	FALSE	Pseudomonadales	Moraxellaceae	Acinetobacter	TRUE
NC_008577	2614818	2614522	98	3.26	367	TRUE	FALSE	Alteromonadales	Shewanellaceae	Shewanella	TRUE
