# Supplementary material for: Missing genes in the annotation of prokaryotic genomes
Source: BMC Bioinformatics. 2010 Mar 15;11:131. doi: 10.1186/1471-2105-11-131 (PMC3098052; doi:10.1186/1471-2105-11-131)
Supplement: Additional file 3 — Table S3. Details for all missing gene groups. Includes cluster id, average α-score, average length, average percent identity, whether the representative sequence had a hit against nr-aa, whether the sequence had a domain result from interproscan, the e-value of the hit to nr-aa, the percent identity of hit to nr-aa, the number of replicons in the group, number of chromsomes, number of plasmids, average MUMi value between families in the group, and whether the multiple alignment of the group indicated a region of ultra-conservation. [file 1471-2105-11-131-S3.PDF]

Cluster ID	Avg Score	Avg Len	Avg Ident	Has NR	IntPro	NR Evalue	NR Ident	# Rep.	# Genes	Chrom	Plasmid	MUMi	Ultra Cons.
7	100	39	100	FALSE	TRUE	NA	NA	6	6	3	3	0.99	TRUE
25	100	58	100	TRUE	TRUE	1.20E-028	100	4	5	4	1	0.99	TRUE
24	100	37	100	FALSE	TRUE	NA	NA	4	5	4	1	0.99	TRUE
53	100	68	100	FALSE	FALSE	NA	NA	3	4	4	0	0.99	TRUE
51	100	44	100	FALSE	FALSE	NA	NA	3	4	4	0	0.99	TRUE
54	100	43	100	TRUE	FALSE	3.50E-013	100	3	4	4	0	0.99	TRUE
49	100	41	100	TRUE	TRUE	2.90E-007	100	3	3	2	1	0.99	TRUE
52	100	39	100	FALSE	FALSE	NA	NA	3	4	4	0	0.99	TRUE
57	100	37	100	FALSE	FALSE	NA	NA	3	4	4	0	0.99	FALSE
60	100	37	100	FALSE	FALSE	NA	NA	3	4	4	0	0.99	TRUE
55	100	35	100	FALSE	TRUE	NA	NA	3	4	4	0	0.99	TRUE
67	100	34	100	FALSE	FALSE	NA	NA	3	3	1	2	0.75	TRUE
50	100	33	100	FALSE	FALSE	NA	NA	3	4	4	0	0.99	TRUE
257	100	99	100	FALSE	TRUE	NA	NA	2	2	2	0	0.92	TRUE
278	100	88	100	FALSE	FALSE	NA	NA	2	2	2	0	0.97	TRUE
329	100	87	100	FALSE	FALSE	NA	NA	2	2	2	0	0.87	TRUE
337	100	81	100	FALSE	FALSE	NA	NA	2	2	2	0	0.84	TRUE
274	100	76	100	FALSE	FALSE	NA	NA	2	2	1	1	0.99	TRUE
273	100	71	100	FALSE	FALSE	NA	NA	2	2	1	1	0.99	TRUE
315	100	67	100	FALSE	FALSE	NA	NA	2	2	2	0	0.87	TRUE
210	100	66	100	FALSE	FALSE	NA	NA	2	2	2	0	0.92	TRUE
282	100	65	100	FALSE	FALSE	NA	NA	2	2	2	0	0.87	TRUE
254	100	63	100	TRUE	TRUE	1.50E-021	100	2	2	2	0	0.92	TRUE
171	100	62	100	FALSE	FALSE	NA	NA	2	2	2	0	0.84	TRUE
342	100	58	100	FALSE	FALSE	NA	NA	2	2	2	0	0.98	TRUE
318	100	58	100	FALSE	TRUE	NA	NA	2	2	2	0	0.84	TRUE
255	100	58	100	FALSE	FALSE	NA	NA	2	2	2	0	0.92	TRUE
114	100	55	100	FALSE	FALSE	NA	NA	2	2	1	1	1	TRUE
205	100	54	100	FALSE	FALSE	NA	NA	2	2	2	0	0.92	TRUE
248	100	54	100	FALSE	FALSE	NA	NA	2	2	2	0	0.92	TRUE
303	100	54	100	FALSE	TRUE	NA	NA	2	2	2	0	0.87	TRUE
272	100	52	100	FALSE	FALSE	NA	NA	2	2	1	1	0.99	FALSE
204	100	52	100	FALSE	FALSE	NA	NA	2	2	2	0	0.92	TRUE
261	100	52	100	FALSE	FALSE	NA	NA	2	2	2	0	0.92	TRUE
137	100	50	100	FALSE	FALSE	NA	NA	2	2	1	1	0.19	TRUE
133	100	47	100	FALSE	FALSE	NA	NA	2	2	1	1	0.19	TRUE
154	100	47	100	FALSE	FALSE	NA	NA	2	2	2	0	0.99	FALSE
259	100	46	100	FALSE	FALSE	NA	NA	2	2	2	0	0.92	TRUE
309	100	46	100	FALSE	FALSE	NA	NA	2	2	2	0	0.87	TRUE
180	100	46	100	FALSE	TRUE	NA	NA	2	2	2	0	0.84	TRUE
312	100	46	100	FALSE	FALSE	NA	NA	2	2	2	0	0.97	TRUE
174	100	45	100	FALSE	FALSE	NA	NA	2	2	2	0	0.84	TRUE
240	100	45	100	FALSE	TRUE	NA	NA	2	2	2	0	0.92	TRUE
201	100	45	100	FALSE	FALSE	NA	NA	2	2	2	0	0.92	TRUE
268	100	45	100	FALSE	FALSE	NA	NA	2	2	1	1	0.99	TRUE
157	100	45	100	FALSE	FALSE	NA	NA	2	2	0	2	0.71	TRUE
117	100	45	100	FALSE	FALSE	NA	NA	2	2	2	0	0.85	FALSE
320	100	44	100	FALSE	FALSE	NA	NA	2	2	2	0	0.84	TRUE
139	100	44	100	FALSE	FALSE	NA	NA	2	2	1	1	0.19	TRUE
277	100	44	100	FALSE	FALSE	NA	NA	2	2	2	0	0.97	TRUE
280	100	43	100	FALSE	TRUE	NA	NA	2	2	2	0	0.97	TRUE
198	100	43	100	FALSE	FALSE	NA	NA	2	2	2	0	0.92	TRUE
243	100	43	100	FALSE	FALSE	NA	NA	2	2	2	0	0.92	TRUE
324	100	42	100	FALSE	FALSE	NA	NA	2	2	2	0	0.84	TRUE
343	100	42	100	FALSE	FALSE	NA	NA	2	2	2	0	0.98	TRUE
213	100	41	100	FALSE	TRUE	NA	NA	2	2	2	0	0.92	TRUE
218	100	41	100	FALSE	FALSE	NA	NA	2	2	2	0	0.96	TRUE
306	100	41	100	FALSE	TRUE	NA	NA	2	2	2	0	0.87	TRUE
182	100	41	100	FALSE	FALSE	NA	NA	2	2	2	0	0.84	TRUE
262	100	41	100	FALSE	FALSE	NA	NA	2	2	2	0	0.92	FALSE
166	100	41	100	FALSE	FALSE	NA	NA	2	2	2	0	0.84	TRUE
271	100	40	100	FALSE	FALSE	NA	NA	2	2	1	1	0.99	TRUE
242	100	40	100	FALSE	FALSE	NA	NA	2	2	2	0	0.92	TRUE
115	100	40	100	FALSE	FALSE	NA	NA	2	3	3	0	0.9	TRUE
241	100	40	100	FALSE	FALSE	NA	NA	2	2	2	0	0.92	FALSE
298	100	39	100	FALSE	FALSE	NA	NA	2	2	2	0	0.87	TRUE
326	100	39	100	FALSE	FALSE	NA	NA	2	2	2	0	0.87	TRUE
265	100	39	100	FALSE	FALSE	NA	NA	2	2	2	0	0.92	TRUE

219	100	39	100	FALSE	FALSE	NA	NA	2	2	2	0	0.92	TRUE
267	100	38	100	FALSE	FALSE	NA	NA	2	2	2	0	0.92	TRUE
288	100	38	100	FALSE	FALSE	NA	NA	2	2	2	0	0.97	TRUE
169	100	38	100	FALSE	FALSE	NA	NA	2	2	2	0	0.84	TRUE
321	100	38	100	FALSE	FALSE	NA	NA	2	2	2	0	0.84	TRUE
212	100	38	100	FALSE	FALSE	NA	NA	2	2	2	0	0.92	TRUE
346	100	37	100	FALSE	FALSE	NA	NA	2	2	2	0	0.98	TRUE
215	100	37	100	FALSE	FALSE	NA	NA	2	2	2	0	0.92	TRUE
327	100	37	100	FALSE	FALSE	NA	NA	2	2	2	0	0.87	TRUE
138	100	37	100	FALSE	FALSE	NA	NA	2	2	1	1	0.19	TRUE
113	100	37	100	FALSE	FALSE	NA	NA	2	2	1	1	1	FALSE
211	100	36	100	FALSE	FALSE	NA	NA	2	3	3	0	0.94	TRUE
333	100	36	100	FALSE	FALSE	NA	NA	2	2	2	0	0.97	TRUE
363	100	36	100	FALSE	TRUE	NA	NA	2	2	1	1	0.66	TRUE
290	100	36	100	FALSE	FALSE	NA	NA	2	2	2	0	0.87	TRUE
281	100	35	100	FALSE	FALSE	NA	NA	2	2	2	0	0.87	FALSE
330	100	35	100	FALSE	FALSE	NA	NA	2	2	2	0	0.87	TRUE
264	100	35	100	FALSE	TRUE	NA	NA	2	2	2	0	0.92	TRUE
345	100	35	100	FALSE	TRUE	NA	NA	2	2	2	0	0.98	TRUE
307	100	35	100	FALSE	FALSE	NA	NA	2	2	2	0	0.87	FALSE
246	100	35	100	FALSE	FALSE	NA	NA	2	2	2	0	0.92	TRUE
208	100	34	100	FALSE	FALSE	NA	NA	2	2	2	0	0.92	TRUE
297	100	34	100	FALSE	FALSE	NA	NA	2	2	2	0	0.87	TRUE
136	100	34	100	FALSE	TRUE	NA	NA	2	2	1	1	0.19	TRUE
260	100	34	100	FALSE	FALSE	NA	NA	2	2	2	0	0.92	TRUE
293	100	34	100	FALSE	FALSE	NA	NA	2	2	2	0	0.87	TRUE
286	100	34	100	FALSE	FALSE	NA	NA	2	2	2	0	0.9	TRUE
263	100	33	100	FALSE	TRUE	NA	NA	2	2	2	0	0.92	TRUE
181	100	33	100	FALSE	TRUE	NA	NA	2	2	2	0	0.97	TRUE
328	100	33	100	FALSE	FALSE	NA	NA	2	2	2	0	0.87	TRUE
313	100	33	100	FALSE	FALSE	NA	NA	2	2	2	0	0.87	TRUE
156	100	33	100	FALSE	FALSE	NA	NA	2	2	2	0	0.99	FALSE
279	100	33	100	FALSE	TRUE	NA	NA	2	2	2	0	0.97	TRUE
287	100	33	100	FALSE	FALSE	NA	NA	2	2	2	0	0.9	TRUE
225	100	33	100	FALSE	FALSE	NA	NA	2	2	2	0	0.99	TRUE
202	99.02	50.33	99.35	FALSE	FALSE	NA	NA	2	3	3	0	0.94	FALSE
155	98.95	95	98.95	FALSE	FALSE	NA	NA	2	2	2	0	0.99	FALSE
302	98.77	81	98.77	FALSE	FALSE	NA	NA	2	2	2	0	0.9	FALSE
3	98.27	33	98.27	FALSE	FALSE	NA	NA	7	7	7	0	0.99	FALSE
63	98.15	54	98.15	FALSE	FALSE	NA	NA	3	3	3	0	0.99	FALSE
102	98.08	52	98.08	FALSE	FALSE	NA	NA	2	2	2	0	0.85	FALSE
116	98	50	98	FALSE	FALSE	NA	NA	2	2	2	0	0.85	FALSE
199	98	50	98	FALSE	FALSE	NA	NA	2	2	2	0	0.92	FALSE
33	97.67	43	97.67	FALSE	FALSE	NA	NA	3	3	3	0	0.94	FALSE
34	97.67	43	97.67	FALSE	FALSE	NA	NA	3	3	3	0	0.94	FALSE
127	97.67	43	97.67	FALSE	FALSE	NA	NA	2	2	2	0	0.98	FALSE
112	97.67	43	97.67	FALSE	FALSE	NA	NA	2	2	2	0	0.85	FALSE
64	97.64	35	98.99	FALSE	FALSE	NA	NA	3	3	3	0	0.9	FALSE
70	97.3	37	97.3	FALSE	FALSE	NA	NA	3	3	0	3	0.02	FALSE
190	97.14	35	97.14	FALSE	FALSE	NA	NA	2	2	0	2	0.42	FALSE
134	96.97	66	96.97	FALSE	FALSE	NA	NA	2	2	1	1	0.19	FALSE
340	96.97	33	96.97	FALSE	TRUE	NA	NA	2	2	2	0	0.98	FALSE
4	96.95	42	97.22	FALSE	FALSE	NA	NA	6	7	7	0	0.95	FALSE
152	96.72	61	96.72	FALSE	FALSE	NA	NA	2	2	2	0	0.99	FALSE
217	96.61	59	96.61	FALSE	FALSE	NA	NA	2	2	2	0	0.92	FALSE
104	96.61	59	96.61	FALSE	FALSE	NA	NA	2	3	3	0	0.9	FALSE
82	96.6	49	96.6	FALSE	FALSE	NA	NA	3	3	3	0	0.9	FALSE
150	96.43	56	96.43	FALSE	FALSE	NA	NA	2	2	2	0	0.99	FALSE
61	96.11	60	96.11	FALSE	FALSE	NA	NA	3	3	3	0	0.99	FALSE
78	95.74	47	95.74	TRUE	FALSE	3.80E-012	74	3	3	3	0	0.9	FALSE
291	95.61	38	95.61	FALSE	FALSE	NA	NA	2	3	3	0	0.87	FALSE
125	95.12	41	95.12	TRUE	FALSE	6.30E-015	100	2	3	3	0	0.98	FALSE
361	95	40	95	FALSE	TRUE	NA	NA	2	2	0	2	0.95	FALSE
344	95	38	95	FALSE	FALSE	NA	NA	2	2	2	0	0.98	FALSE
294	94.74	144	94.74	FALSE	FALSE	NA	NA	2	2	2	0	0.9	FALSE
220	94.59	37	94.59	FALSE	FALSE	NA	NA	2	2	2	0	0.99	FALSE
130	93.94	33	93.94	FALSE	FALSE	NA	NA	2	3	3	0	0.98	FALSE
79	93.67	75	93.67	FALSE	FALSE	NA	NA	3	4	4	0	0.91	FALSE
128	92.4	65.67	91.45	TRUE	TRUE	1.30E-030	96	2	3	3	0	0.98	FALSE
76	92.32	43.5	92.59	FALSE	FALSE	NA	NA	3	4	4	0	0.93	FALSE

341	92.31	39	92.31	FALSE	FALSE	NA	NA	2	2	2	0	0.98	FALSE
147	92.16	51	92.16	FALSE	FALSE	NA	NA	2	2	2	0	0.99	FALSE
84	92.03	38.33	89.62	FALSE	FALSE	NA	NA	3	3	3	0	0.94	FALSE
86	91.67	39	91.67	FALSE	FALSE	NA	NA	3	4	4	0	0.9	FALSE
378	91.54	42.5	91.54	FALSE	FALSE	NA	NA	2	2	2	0	0.98	FALSE
269	91.45	37	91.45	FALSE	FALSE	NA	NA	2	2	2	0	0.98	FALSE
10	91.36	44	91.36	FALSE	FALSE	NA	NA	5	5	5	0	0.99	FALSE
11	91.19	60.4	91.2	FALSE	FALSE	NA	NA	5	5	2	3	0.27	FALSE
304	91.18	34	91.18	FALSE	TRUE	NA	NA	2	2	2	0	0.92	FALSE
80	90.99	40.67	90.32	FALSE	FALSE	NA	NA	3	3	3	0	0.88	FALSE
237	90.91	44	90.91	FALSE	FALSE	NA	NA	2	2	2	0	0.98	FALSE
20	90.44	34	90.44	FALSE	FALSE	NA	NA	4	4	4	0	0.97	FALSE
87	90.33	42	90.33	FALSE	FALSE	NA	NA	3	3	3	0	0.93	FALSE
145	90.3	67	90.3	FALSE	FALSE	NA	NA	2	2	2	0	0.99	FALSE
207	89.86	69	89.86	FALSE	FALSE	NA	NA	2	2	2	0	0.92	FALSE
77	89.86	46	89.86	FALSE	FALSE	NA	NA	3	3	3	0	0.9	FALSE
66	89.74	39	89.74	FALSE	FALSE	NA	NA	3	3	3	0	0.9	FALSE
31	89.11	50.33	88.89	FALSE	FALSE	NA	NA	3	3	3	0	0.92	FALSE
46	88.89	39	88.89	FALSE	FALSE	NA	NA	3	3	3	0	0.99	FALSE
250	88.89	99	88.89	FALSE	TRUE	NA	NA	2	2	2	0	0.92	FALSE
105	88.89	66	88.89	FALSE	FALSE	NA	NA	2	3	3	0	0.9	FALSE
88	88.6	38	88.6	FALSE	TRUE	NA	NA	3	3	3	0	0.99	FALSE
75	88.46	39	88.46	FALSE	FALSE	NA	NA	3	4	4	0	0.93	FALSE
191	88.46	52	88.46	FALSE	FALSE	NA	NA	2	2	0	2	0.42	FALSE
32	88.27	50.33	89.36	FALSE	FALSE	NA	NA	3	3	3	0	0.92	FALSE
101	88.24	51	88.24	FALSE	FALSE	NA	NA	2	9	9	0	0.96	FALSE
107	88.12	34	88.12	FALSE	FALSE	NA	NA	2	2	2	0	0.97	FALSE
311	87.6	36.33	87.24	FALSE	FALSE	NA	NA	2	3	3	0	0.98	FALSE
39	87.14	56	87.14	FALSE	FALSE	NA	NA	3	10	10	0	0.96	FALSE
111	86.23	138	86.23	FALSE	FALSE	NA	NA	2	3	3	0	0.9	FALSE
65	86.08	42	85.16	FALSE	FALSE	NA	NA	3	3	3	0	0.9	FALSE
256	86.05	86	86.05	FALSE	FALSE	NA	NA	2	2	2	0	0.92	FALSE
159	85.46	79.5	85.44	FALSE	FALSE	NA	NA	2	2	1	1	0.71	FALSE
131	85.37	33.67	86.09	FALSE	FALSE	NA	NA	2	3	3	0	0.98	FALSE
353	85.15	34	85.15	FALSE	FALSE	NA	NA	2	2	2	0	1	FALSE
71	84.67	55.33	85.71	FALSE	FALSE	NA	NA	3	3	3	0	0.93	FALSE
158	84.42	33	84.42	FALSE	TRUE	NA	NA	2	2	2	0	0.98	FALSE
81	84.34	36	86.47	FALSE	FALSE	NA	NA	3	3	3	0	0.88	FALSE
366	84.17	83	84.17	FALSE	TRUE	NA	NA	2	2	1	1	0.66	FALSE
18	84.14	43.25	83.97	FALSE	FALSE	NA	NA	4	4	4	0	0.97	FALSE
336	84.03	37	84.03	FALSE	FALSE	NA	NA	2	2	2	0	0.97	FALSE
175	82.93	41	82.93	FALSE	FALSE	NA	NA	2	2	2	0	1	FALSE
38	82.73	38.44	81.93	FALSE	FALSE	NA	NA	3	9	9	0	0.96	FALSE
253	82.67	75	82.67	FALSE	FALSE	NA	NA	2	2	2	0	0.92	FALSE
299	82.56	86	82.56	FALSE	FALSE	NA	NA	2	2	2	0	0.87	FALSE
19	80.95	42	80.95	FALSE	FALSE	NA	NA	4	4	3	1	1	FALSE
140	80.43	46	80.43	FALSE	TRUE	NA	NA	2	2	1	1	0.19	FALSE
124	79.55	62	79.55	FALSE	FALSE	NA	NA	2	2	2	0	0.96	FALSE
153	79.45	73	79.45	FALSE	FALSE	NA	NA	2	2	2	0	0.99	FALSE
308	79.27	35.5	79.27	TRUE	FALSE	1.20E-010	83	2	2	2	0	0.98	FALSE
118	78.85	52	78.85	FALSE	FALSE	NA	NA	2	2	2	0	0.99	FALSE
164	78.56	48.5	78.56	FALSE	FALSE	NA	NA	2	2	2	0	1	FALSE
322	78.18	55	78.18	FALSE	FALSE	NA	NA	2	2	2	0	0.97	FALSE
295	77.14	35	77.14	FALSE	TRUE	NA	NA	2	2	2	0	0.87	FALSE
222	76.79	38	76.79	FALSE	FALSE	NA	NA	2	2	1	1	1	FALSE
305	76.74	43	76.74	FALSE	FALSE	NA	NA	2	2	2	0	0.87	FALSE
62	76.06	47	75.3	FALSE	FALSE	NA	NA	3	3	1	2	1	FALSE
73	75.82	40	76.85	FALSE	FALSE	NA	NA	3	3	3	0	0.94	FALSE
360	75.61	41	75.61	FALSE	FALSE	NA	NA	2	2	2	0	0.92	FALSE
59	75.34	38	76.32	FALSE	FALSE	NA	NA	3	3	3	0	1	FALSE
110	75.27	51	74.54	FALSE	FALSE	NA	NA	2	2	2	0	0.84	FALSE
178	75.06	36	75.06	FALSE	FALSE	NA	NA	2	2	2	0	1	FALSE
300	75	48	75	FALSE	FALSE	NA	NA	2	2	2	0	0.87	FALSE
135	74.42	43	74.42	TRUE	FALSE	2.30E-009	74	2	2	1	1	0.19	FALSE
258	73.67	78	100	TRUE	FALSE	4.00E-006	60	2	2	2	0	0.92	FALSE
170	73.33	45	73.33	FALSE	FALSE	NA	NA	2	3	3	0	0.99	FALSE
270	72.73	44	72.73	FALSE	FALSE	NA	NA	2	2	1	1	0.99	FALSE
123	72.73	36	72.73	TRUE	FALSE	1.40E-009	90	2	2	2	0	0.99	FALSE
301	72.3	41.5	72.3	FALSE	TRUE	NA	NA	2	2	2	0	0.96	FALSE
317	72.09	43	72.09	FALSE	FALSE	NA	NA	2	2	2	0	0.93	FALSE

285	72	50	72	FALSE	FALSE	NA	NA	2	2	2	0	0.9	FALSE
163	71.66	35	71.66	FALSE	FALSE	NA	NA	2	2	2	0	0.99	FALSE
200	71.25	62	100	TRUE	FALSE	2.00E-006	59	2	2	2	0	0.92	FALSE
176	71.25	36.5	71.25	FALSE	FALSE	NA	NA	2	2	2	0	1	FALSE
43	71.24	51	71.24	FALSE	FALSE	NA	NA	3	3	3	0	0.84	FALSE
247	71.24	62	100	FALSE	FALSE	NA	NA	2	2	2	0	0.92	FALSE
251	71.19	59	71.19	FALSE	FALSE	NA	NA	2	2	2	0	0.92	FALSE
283	71.15	52	71.15	FALSE	TRUE	NA	NA	2	2	2	0	0.97	FALSE
203	71.11	45	71.11	FALSE	FALSE	NA	NA	2	3	3	0	0.94	FALSE
89	70.93	46.67	70	FALSE	FALSE	NA	NA	3	3	3	0	0.92	FALSE
266	70.45	44	70.45	FALSE	FALSE	NA	NA	2	2	2	0	0.92	FALSE
37	69.37	37	69.37	FALSE	FALSE	NA	NA	3	3	3	0	0.83	FALSE
350	69.3	72	69.3	FALSE	FALSE	NA	NA	2	3	3	0	0.99	FALSE
284	69.23	39	69.23	FALSE	FALSE	NA	NA	2	2	2	0	0.97	FALSE
323	69.09	55	69.09	FALSE	FALSE	NA	NA	2	2	2	0	0.84	FALSE
8	68.44	40	92.92	FALSE	FALSE	NA	NA	5	6	6	0	0.95	FALSE
22	68.2	37.75	66.38	FALSE	FALSE	NA	NA	4	4	3	1	1	FALSE
373	68.09	47	68.09	FALSE	FALSE	NA	NA	2	2	2	0	0.96	FALSE
179	67.9	51	74.51	FALSE	FALSE	NA	NA	2	2	2	0	0.95	FALSE
44	67.62	35.33	67.94	FALSE	TRUE	NA	NA	3	3	1	2	1	FALSE
121	67.38	47.5	67.38	FALSE	FALSE	NA	NA	2	2	2	0	0.95	FALSE
172	66.87	36	66.87	FALSE	FALSE	NA	NA	2	2	2	0	0.98	FALSE
74	66.74	37	66.74	FALSE	FALSE	NA	NA	3	5	5	0	0.99	FALSE
196	66.05	77	66.06	FALSE	FALSE	NA	NA	2	2	2	0	0.98	FALSE
151	65.68	53	65.68	FALSE	FALSE	NA	NA	2	2	2	0	0.99	FALSE
108	65.65	112	100	FALSE	TRUE	NA	NA	2	2	2	0	0.9	FALSE
40	65.27	65.33	65.11	FALSE	FALSE	NA	NA	3	3	3	0	0.9	FALSE
5	65.18	60	74.86	FALSE	FALSE	NA	NA	6	7	7	0	0.95	FALSE
56	64.73	71	64.73	FALSE	TRUE	NA	NA	3	4	4	0	0.99	FALSE
227	64.37	78	64.37	FALSE	FALSE	NA	NA	2	2	2	0	0.98	FALSE
192	64.08	51.5	64.08	FALSE	FALSE	NA	NA	2	2	2	0	0.94	FALSE
15	63.95	46.2	64.5	FALSE	FALSE	NA	NA	5	5	5	0	0.94	FALSE
17	63.93	61.63	62.78	FALSE	FALSE	NA	NA	4	8	8	0	0.98	FALSE
223	63.56	53.5	63.45	TRUE	FALSE	2.20E-009	55	2	2	2	0	0.99	FALSE
146	62.96	54	62.96	FALSE	FALSE	NA	NA	2	2	2	0	0.99	FALSE
97	62.73	59	62.73	FALSE	FALSE	NA	NA	2	2	2	0	0.93	FALSE
356	62.61	51.5	62.61	FALSE	FALSE	NA	NA	2	2	2	0	0.96	FALSE
214	62.16	37	62.16	FALSE	FALSE	NA	NA	2	2	2	0	0.92	FALSE
197	62.07	58	62.07	FALSE	FALSE	NA	NA	2	2	2	0	0.92	FALSE
95	61.94	43.5	61.94	FALSE	FALSE	NA	NA	2	2	2	0	0.99	FALSE
335	61.57	77	100	FALSE	FALSE	NA	NA	2	2	2	0	0.84	FALSE
148	61.17	98	94.9	TRUE	FALSE	5.60E-017	79	2	2	2	0	0.99	FALSE
331	60.8	69	72.46	FALSE	FALSE	NA	NA	2	2	2	0	0.92	FALSE
21	60.78	54	63.23	FALSE	FALSE	NA	NA	4	4	4	0	0.96	FALSE
351	60.76	80	61.2	FALSE	FALSE	NA	NA	2	2	2	0	0.99	FALSE
358	60.72	72.5	60.72	FALSE	FALSE	NA	NA	2	2	2	0	0.97	FALSE
221	60.39	51	60.39	TRUE	FALSE	2.80E-007	60	2	2	1	1	0.66	FALSE
232	60.09	46	62.03	TRUE	TRUE	3.90E-009	71	2	2	0	2	0.87	FALSE
99	59.95	48.5	59.95	FALSE	FALSE	NA	NA	2	2	2	0	0.95	FALSE
276	59.59	75	100	FALSE	TRUE	NA	NA	2	2	2	0	0.97	FALSE
160	59.56	42	59.56	TRUE	FALSE	1.30E-007	60	2	2	1	1	1	FALSE
347	59.46	37	59.46	FALSE	FALSE	NA	NA	2	2	2	0	0.96	FALSE
72	59.26	70.33	59.42	FALSE	FALSE	NA	NA	3	3	3	0	0.97	FALSE
106	59.04	112	83.93	FALSE	FALSE	NA	NA	2	2	2	0	0.9	FALSE
26	59.02	45	81.08	FALSE	FALSE	NA	NA	4	4	4	0	0.98	FALSE
338	59	50	59	FALSE	TRUE	NA	NA	2	2	2	0	0.99	FALSE
370	58.59	39	97.44	FALSE	FALSE	NA	NA	2	2	2	0	0.85	FALSE
177	58.24	79	58.24	FALSE	FALSE	NA	NA	2	2	2	0	1	FALSE
325	58.18	110	100	FALSE	FALSE	NA	NA	2	2	2	0	0.97	FALSE
68	58.02	54	58.02	FALSE	FALSE	NA	NA	3	3	3	0	0.98	FALSE
122	57.48	102	56.91	FALSE	TRUE	NA	NA	2	3	1	2	1	FALSE
93	56.91	60	100	FALSE	FALSE	NA	NA	2	2	2	0	0.86	FALSE
129	56.78	63.5	56.78	TRUE	FALSE	5.40E-024	89	2	2	2	0	0.98	FALSE
372	56.29	62.5	56.29	FALSE	FALSE	NA	NA	2	2	1	1	0.97	FALSE
316	55.89	66	77.72	FALSE	FALSE	NA	NA	2	2	2	0	0.93	FALSE
275	55.8	45	55.8	FALSE	FALSE	NA	NA	2	2	2	0	1	FALSE
126	55.61	54.5	55.61	FALSE	FALSE	NA	NA	2	2	2	0	1	FALSE
229	55.51	49	100	FALSE	FALSE	NA	NA	2	2	2	0	0.98	FALSE
239	55.37	49	100	FALSE	FALSE	NA	NA	2	2	2	0	0.92	FALSE
85	55.06	82.33	98	FALSE	FALSE	NA	NA	3	3	3	0	0.97	FALSE

377	54.97	65.5	54.97	TRUE	TRUE	6.50E-014	60	2	2	2	0	1	FALSE
319	54.78	116	87.07	FALSE	FALSE	NA	NA	2	2	2	0	0.84	FALSE
58	53.44	67	100	TRUE	FALSE	1.20E-015	73	3	4	4	0	0.99	FALSE
0	53.42	50.34	53.25	FALSE	FALSE	NA	NA	16	87	82	5	0.99	FALSE
369	53.31	66	96.97	FALSE	FALSE	NA	NA	2	2	2	0	0.85	FALSE
235	52.38	84	52.38	TRUE	FALSE	8.80E-013	62	2	4	4	0	0.99	FALSE
96	51.85	54	51.85	FALSE	FALSE	NA	NA	2	2	2	0	0.99	FALSE
349	51.43	70	51.43	FALSE	FALSE	NA	NA	2	2	2	0	0.98	FALSE
35	51.17	53.33	49.58	FALSE	FALSE	NA	NA	3	6	6	0	0.99	FALSE
374	51.13	66.5	51.13	TRUE	FALSE	2.00E-015	56	2	2	2	0	0.99	FALSE
83	50.64	41.67	91.46	FALSE	FALSE	NA	NA	3	3	3	0	0.93	FALSE
206	50.37	58	50.37	FALSE	FALSE	NA	NA	2	2	2	0	0.97	FALSE
236	50.1	59.5	50.09	TRUE	FALSE	5.20E-027	100	2	2	2	0	0.99	FALSE
162	49.71	41	100	FALSE	FALSE	NA	NA	2	2	2	0	0.84	FALSE
23	49.65	70.25	49.82	TRUE	FALSE	1.20E-007	49	4	4	4	0	0.99	FALSE
187	49.21	63	49.21	TRUE	FALSE	1.20E-007	60	2	2	2	0	1	FALSE
144	48.87	66.5	48.84	FALSE	TRUE	NA	NA	2	2	2	0	0.98	FALSE
357	48.83	45.5	48.83	FALSE	FALSE	NA	NA	2	2	2	0	0.96	FALSE
292	48.78	60	100	FALSE	FALSE	NA	NA	2	2	2	0	0.87	FALSE
143	48.72	76	48.72	TRUE	FALSE	4.70E-030	100	2	2	2	0	0.99	FALSE
249	48.67	47	100	FALSE	FALSE	NA	NA	2	2	2	0	0.92	FALSE
296	48.52	51	98.04	FALSE	FALSE	NA	NA	2	2	2	0	0.87	FALSE
132	48.06	58	98.28	TRUE	FALSE	1.20E-026	98	2	2	2	0	0.98	FALSE
244	47.98	96	100	TRUE	FALSE	7.90E-011	77	2	2	2	0	0.92	FALSE
90	47.97	84.5	48.06	TRUE	TRUE	7.50E-020	60	2	2	2	0	0.99	FALSE
69	47.45	136.67	47.56	TRUE	FALSE	3.40E-079	100	3	3	3	0	1	FALSE
16	47.27	34.4	97.37	TRUE	FALSE	6.50E-007	75	5	5	5	0	0.95	FALSE
92	46.9	79	46.9	FALSE	FALSE	NA	NA	2	2	2	0	0.92	FALSE
161	46.73	35	85.71	TRUE	FALSE	9.00E-006	75	2	6	6	0	1	FALSE
36	46.44	75	47.72	FALSE	FALSE	NA	NA	3	3	3	0	0.98	FALSE
209	46.27	114	100	FALSE	FALSE	NA	NA	2	2	2	0	0.92	FALSE
195	45.76	50.5	45.59	TRUE	FALSE	2.90E-012	60	2	2	2	0	0.98	FALSE
12	45.76	40.4	84	FALSE	FALSE	NA	NA	5	5	5	0	0.9	FALSE
371	45.23	80.5	45.42	TRUE	FALSE	1.90E-007	45	2	2	2	0	0.74	FALSE
245	45	60	81.67	FALSE	FALSE	NA	NA	2	2	2	0	0.92	FALSE
91	44.87	64.5	44.87	TRUE	TRUE	9.60E-010	51	2	2	2	0	0.9	FALSE
238	44.12	39	100	FALSE	FALSE	NA	NA	2	2	2	0	0.92	FALSE
14	43.64	49	99.4	FALSE	FALSE	NA	NA	5	5	5	0	0.96	FALSE
233	43.48	80.5	43.48	TRUE	FALSE	3.50E-041	98	2	2	2	0	0.99	FALSE
252	43.46	41	100	FALSE	FALSE	NA	NA	2	2	2	0	0.92	FALSE
29	43.13	46.45	79.22	TRUE	FALSE	5.00E-012	80	4	11	11	0	0.98	FALSE
30	43.06	95.33	90.37	FALSE	FALSE	NA	NA	3	3	3	0	0.9	FALSE
27	42.62	97.8	89.92	FALSE	FALSE	NA	NA	4	5	5	0	0.87	FALSE
94	42.47	119.5	42.47	TRUE	FALSE	4.00E-006	44	2	2	2	0	0.99	FALSE
310	42.43	58	60.51	FALSE	FALSE	NA	NA	2	2	2	0	0.9	FALSE
352	41.67	108	41.67	FALSE	FALSE	NA	NA	2	2	2	0	1	FALSE
120	41.48	43	100	FALSE	FALSE	NA	NA	2	2	2	0	0.84	FALSE
355	41.35	71.67	40.84	TRUE	TRUE	8.50E-011	54	2	3	3	0	0.99	FALSE
28	40.95	63	73.41	FALSE	FALSE	NA	NA	4	4	4	0	0.92	FALSE
375	40.92	67.5	58.13	TRUE	FALSE	5.40E-008	58	2	2	2	0	0.96	FALSE
194	40.81	37	89.19	TRUE	FALSE	1.80E-009	88	2	4	3	1	0.99	FALSE
6	40.71	51	84.83	TRUE	FALSE	1.10E-008	66	6	19	19	0	1	FALSE
13	40.48	60.3	70.21	TRUE	TRUE	2.80E-009	64	5	10	9	1	0.99	FALSE
289	40.29	72	80.56	TRUE	FALSE	4.50E-012	62	2	2	2	0	0.87	FALSE
103	39.85	61.67	52.13	FALSE	TRUE	NA	NA	2	3	3	0	0.89	FALSE
42	39.7	52.67	38.48	TRUE	FALSE	2.10E-007	51	3	3	3	0	1	FALSE
348	39.39	77	36.36	FALSE	FALSE	NA	NA	2	2	2	0	0.99	FALSE
189	38.68	90	61.95	FALSE	FALSE	NA	NA	2	2	2	0	0.98	FALSE
141	38.52	71.33	37.3	TRUE	FALSE	4.00E-008	46	2	3	3	0	0.99	FALSE
142	38.47	104	37.65	FALSE	FALSE	NA	NA	2	2	2	0	1	FALSE
173	38.09	44.33	85.72	FALSE	FALSE	NA	NA	2	3	1	2	0.98	FALSE
216	38.05	69.5	85.25	FALSE	TRUE	NA	NA	2	2	2	0	0.92	FALSE
339	37.79	90.5	37.79	FALSE	TRUE	NA	NA	2	2	2	0	0.99	FALSE
364	37.43	49	71.43	FALSE	FALSE	NA	NA	2	3	3	0	0.99	FALSE
149	37.38	53	81.13	FALSE	FALSE	NA	NA	2	8	8	0	0.99	FALSE
230	37.34	62	37.34	TRUE	FALSE	1.60E-028	100	2	2	2	0	0.99	FALSE
368	37.31	53	51.4	FALSE	TRUE	NA	NA	2	2	2	0	1	FALSE
379	37.03	46	89.07	FALSE	FALSE	NA	NA	2	2	2	0	0.86	FALSE
119	36.78	53.5	52.45	FALSE	FALSE	NA	NA	2	2	2	0	0.91	FALSE
2	36.75	42	100	FALSE	FALSE	NA	NA	8	11	5	6	0.97	FALSE

365	36.46	51	72.55	FALSE	FALSE	NA	NA	2	3	3	0	1	FALSE
226	36.15	119	36.19	FALSE	FALSE	NA	NA	2	2	1	1	1	FALSE
188	35.63	92	35.63	FALSE	FALSE	NA	NA	2	2	2	0	1	FALSE
376	35.06	88.5	35.06	TRUE	TRUE	4.40E-033	74	2	2	2	0	1	FALSE
45	34.67	74.67	34.83	FALSE	TRUE	NA	NA	3	3	3	0	1	FALSE
167	34.21	45	73.33	FALSE	TRUE	NA	NA	2	2	2	0	0.84	FALSE
1	34.02	49.88	63.84	FALSE	FALSE	NA	NA	16	16	16	0	0.98	FALSE
186	33.24	68	50.09	TRUE	TRUE	6.90E-008	54	2	2	2	0	1	FALSE
359	32.83	82.67	60.61	TRUE	FALSE	1.50E-007	68	2	3	3	0	0.97	FALSE
332	32.34	54	74.07	TRUE	FALSE	3.00E-006	85	2	2	2	0	0.92	FALSE
9	31.8	52.8	68.21	FALSE	FALSE	NA	NA	5	5	5	0	0.97	FALSE
100	31.57	111	31.57	TRUE	TRUE	7.70E-061	97	2	2	2	0	1	FALSE
185	31.03	41	64.63	FALSE	FALSE	NA	NA	2	14	14	0	0.99	FALSE
362	30.9	163.5	30.91	TRUE	FALSE	3.10E-013	64	2	2	2	0	1	FALSE
168	30.85	67.5	42.97	FALSE	FALSE	NA	NA	2	2	2	0	0.99	FALSE
193	28.74	143	28.74	FALSE	FALSE	NA	NA	2	2	2	0	0.99	FALSE
165	28.72	44	90.91	TRUE	TRUE	7.40E-016	88	2	6	6	0	1	FALSE
184	27.92	62	59.68	TRUE	FALSE	1.80E-008	54	2	14	14	0	1	FALSE
334	26	71	100	TRUE	FALSE	2.80E-030	88	2	2	2	0	0.84	FALSE
314	25.37	84	79.76	TRUE	FALSE	7.70E-009	68	2	2	2	0	0.96	FALSE
48	25.35	177.67	25.33	TRUE	FALSE	6.60E-026	36	3	3	3	0	1	FALSE
224	23.05	51	100	TRUE	FALSE	6.20E-007	65	2	2	2	0	1	FALSE
109	22.99	52	76.92	TRUE	FALSE	3.80E-009	75	2	4	4	0	0.96	FALSE
231	22.77	41	86.59	FALSE	TRUE	NA	NA	2	2	2	0	1	FALSE
47	22.67	40.33	78.84	FALSE	TRUE	NA	NA	3	3	3	0	0.99	FALSE
234	19.64	88.75	50.81	TRUE	FALSE	8.00E-006	68	2	4	4	0	0.99	FALSE
354	19.26	54.5	58.72	FALSE	FALSE	NA	NA	2	2	1	1	1	FALSE
41	14.89	83.67	53.02	FALSE	FALSE	NA	NA	3	3	3	0	0.95	FALSE
183	14.36	58.67	49.99	FALSE	TRUE	NA	NA	2	3	3	0	0.99	FALSE
98	14	121	36.37	FALSE	FALSE	NA	NA	2	2	2	0	0.99	FALSE
228	11.53	124.11	41	FALSE	FALSE	NA	NA	2	9	9	0	1	FALSE
367	9.12	100	55.67	TRUE	FALSE	2.10E-056	100	2	2	2	0	0.99	FALSE
