# Supplementary material for: Missing genes in the annotation of prokaryotic genomes
Source: BMC Bioinformatics. 2010 Mar 15;11:131. doi: 10.1186/1471-2105-11-131 (PMC3098052; doi:10.1186/1471-2105-11-131)
Supplement: Additional file 6 — InterPro domain results. InterProScan results for the representative amino acid sequence for each group. [file 1471-2105-11-131-S6.PDF]

Group ID	Scan Type	Result
100	tmhmm	transmembrane_regions
100	SignalP	signal-peptide
103	SignalP	signal-peptide
103	tmhmm	transmembrane_regions
108	SignalP	signal-peptide
122	tmhmm	transmembrane_regions
122	SignalP	signal-peptide
128	SignalP	signal-peptide
158	SignalP	signal-peptide
165	tmhmm	transmembrane_regions
167	SignalP	signal-peptide
167	tmhmm	transmembrane_regions
180	SignalP	signal-peptide
181	SignalP	signal-peptide
183	SignalP	signal-peptide
186	SignalP	signal-peptide
196	PS00028	ZINC_FINGER_C2H2_1
213	SignalP	signal-peptide
216	SignalP	signal-peptide
223	tmhmm	transmembrane_regions
231	SignalP	signal-peptide
232	SignalP	signal-peptide
232	PF09604	Potass_KdpF
232	tmhmm	transmembrane_regions
232	TIGR02115	potass_kdpF: K+ ATPase
240	SignalP	signal-peptide
250	SignalP	signal-peptide
250	tmhmm	transmembrane_regions
254	SignalP	signal-peptide
257	tmhmm	transmembrane_regions
25	SignalP	signal-peptide
263	SignalP	signal-peptide
264	SignalP	signal-peptide
276	SignalP	signal-peptide
279	SignalP	signal-peptide
280	SignalP	signal-peptide
283	tmhmm	transmembrane_regions
283	SignalP	signal-peptide
295	SignalP	signal-peptide
301	tmhmm	transmembrane_regions
301	SignalP	signal-peptide
303	tmhmm	transmembrane_regions
304	SignalP	signal-peptide

306	PS51300	NIRD
318	SignalP	signal-peptide
355	tmhmm	transmembrane_regions
355	PD019268	Q3ET64_BACTI_Q3ET64;
361	SignalP	signal-peptide
361	tmhmm	transmembrane_regions
363	SignalP	signal-peptide
366	tmhmm	transmembrane_regions
366	SignalP	signal-peptide
368	SignalP	signal-peptide
376	tmhmm	transmembrane_regions
376	tmhmm	transmembrane_regions
376	SignalP	signal-peptide
377	PF05930	Phage_AlpA
377	SSF46955	Putative DNA-binding
385	SignalP	signal-peptide
386	PS00615	C_TYPE_LECTIN_1
391	SignalP	signal-peptide
396	SignalP	signal-peptide
44	SignalP	signal-peptide
44	tmhmm	transmembrane_regions
45	PS01108	RIBOSOMAL_L24
47	SignalP	signal-peptide
47	tmhmm	transmembrane_regions
49	SignalP	signal-peptide
55	SignalP	signal-peptide
56	SignalP	signal-peptide
7	SignalP	signal-peptide
88	SignalP	signal-peptide
90	SignalP	signal-peptide
90	tmhmm	transmembrane_regions
90	tmhmm	transmembrane_regions
91	PF09926	DUF2158
